# Deciphering core phyllomicrobiome assemblage on rice genotypes grown in contrasting agroclimatic zones: implications for phyllomicrobiome engineering against blast disease

**DOI:** 10.1186/s40793-022-00421-5

**Published:** 2022-05-26

**Authors:** Kuleshwar Prasad Sahu, A. Kumar, K. Sakthivel, Bhaskar Reddy, Mukesh Kumar, Asharani Patel, Neelam Sheoran, Subbaiyan Gopalakrishnan, Ganesan Prakash, Rajeev Rathour, R. K. Gautam

**Affiliations:** 1 Division of Plant Pathology, ICAR - Indian Agricultural Research Institute, New Delhi, 110012 India; 2 Division of Field Crop Improvement and Protection, ICAR-Central Island Agricultural Research Institute, Port Blair, Andaman and Nicobar Islands 744101 India; 3 Division of Genetics, ICAR-Indian Agricultural Research Institute, New Delhi, 110012 India; 4 Department of Agricultural Biotechnology, CSK Himachal Pradesh Agricultural University, Palampur, Himachal Pradesh 176062 India

**Keywords:** Antibiosis, Bacterial volatiles, Blast, Core Microbiome, Defense genes, *Magnaporthe oryzae*, Phyllomicrobiome, Phyllosphere, Rice

## Abstract

**Background:**

With its adapted microbial diversity, the phyllosphere contributes microbial metagenome to the plant holobiont and modulates a host of ecological functions. Phyllosphere microbiome (hereafter termed phyllomicrobiome) structure and the consequent ecological functions are vulnerable to a host of biotic (Genotypes) and abiotic factors (Environment) which is further compounded by agronomic transactions. However, the ecological forces driving the phyllomicrobiome assemblage and functions are among the most understudied aspects of plant biology. Despite the reports on the occurrence of diverse prokaryotic phyla such as Proteobacteria, Firmicutes, Bacteroides, and Actinobacteria in phyllosphere habitat, the functional characterization leading to their utilization for agricultural sustainability is not yet explored.

Currently, the metabarcoding by Next-Generation-Sequencing (mNGS) technique is a widely practised strategy for microbiome investigations. However, the validation of mNGS annotations by culturomics methods is not integrated with the microbiome exploration program. In the present study, we combined the mNGS with culturomics to decipher the core functional phyllomicrobiome of rice genotypes varying for blast disease resistance planted in two agroclimatic zones in India. There is a growing consensus among the various stakeholder of rice farming for an ecofriendly method of disease management. Here, we proposed phyllomicrobiome assisted rice blast management as a novel strategy for rice farming in the future.

**Results:**

The tropical "Island Zone" displayed marginally more bacterial diversity than that of the temperate ‘Mountain Zone’ on the phyllosphere. Principal coordinate analysis indicated converging phyllomicrobiome profiles on rice genotypes sharing the same agroclimatic zone. Interestingly, the rice genotype grown in the contrasting zones displayed divergent phyllomicrobiomes suggestive of the role of environment on phyllomicrobiome assembly. The predominance of phyla such as Proteobacteria, Actinobacteria, and Firmicutes was observed in the phyllosphere irrespective of the genotypes and climatic zones. The core-microbiome analysis revealed an association of *Acidovorax, Arthrobacter, Bacillus, Clavibacter, Clostridium, Cronobacter, Curtobacterium, Deinococcus, Erwinia, Exiguobacterium, Hymenobacter, Kineococcus, Klebsiella, Methylobacterium, Methylocella, Microbacterium, Nocardioides, Pantoea, Pedobacter, Pseudomonas, Salmonella, Serratia, Sphingomonas* and *Streptomyces* on phyllosphere. The linear discriminant analysis (LDA) effect size (LEfSe) method revealed distinct bacterial genera in blast-resistant and susceptible genotypes, as well as mountain and island climate zones. SparCC based network analysis of phyllomicrobiome showed complex intra-microbial cooperative or competitive interactions on the rice genotypes. The culturomic validation of mNGS data confirmed the occurrence of *Acinetobacter, Aureimonas, Curtobacterium, Enterobacter, Exiguobacterium, Microbacterium, Pantoea, Pseudomonas,* and *Sphingomonas* in the phyllosphere. Strikingly, the contrasting agroclimatic zones showed genetically identical bacterial isolates suggestive of vertical microbiome transmission. The core-phyllobacterial communities showed secreted and volatile compound mediated antifungal activity on *M. oryzae.* Upon phyllobacterization (a term coined for spraying bacterial cells on the phyllosphere), *Acinetobacter, Aureimonas*, *Pantoea*, *and Pseudomonas* conferred immunocompetence against blast disease. Transcriptional analysis revealed activation of defense genes such as *OsPR1.1*, *OsNPR1, OsPDF2.2*, *OsFMO, OsPAD4, OsCEBiP*, and *OsCERK1* in phyllobacterized rice seedlings.

**Conclusions:**

PCoA indicated the key role of agro-climatic zones to drive phyllomicrobiome assembly on the rice genotypes. The mNGS and culturomic methods showed *Acinetobacter, Aureimonas, Curtobacterium, Enterobacter, Exiguobacterium, Microbacterium, Pantoea, Pseudomonas,* and *Sphingomonas* as core phyllomicrobiome of rice. Genetically identical *Pantoea* intercepted on the phyllosphere from the well-separated agroclimatic zones is suggestive of vertical transmission of phyllomicrobiome. The phyllobacterization showed potential for blast disease suppression by direct antibiosis and defense elicitation. Identification of functional core-bacterial communities on the phyllosphere and their co-occurrence dynamics presents an opportunity to devise novel strategies for rice blast management through phyllomicrobiome reengineering in the future.

**Graphical abstract:**

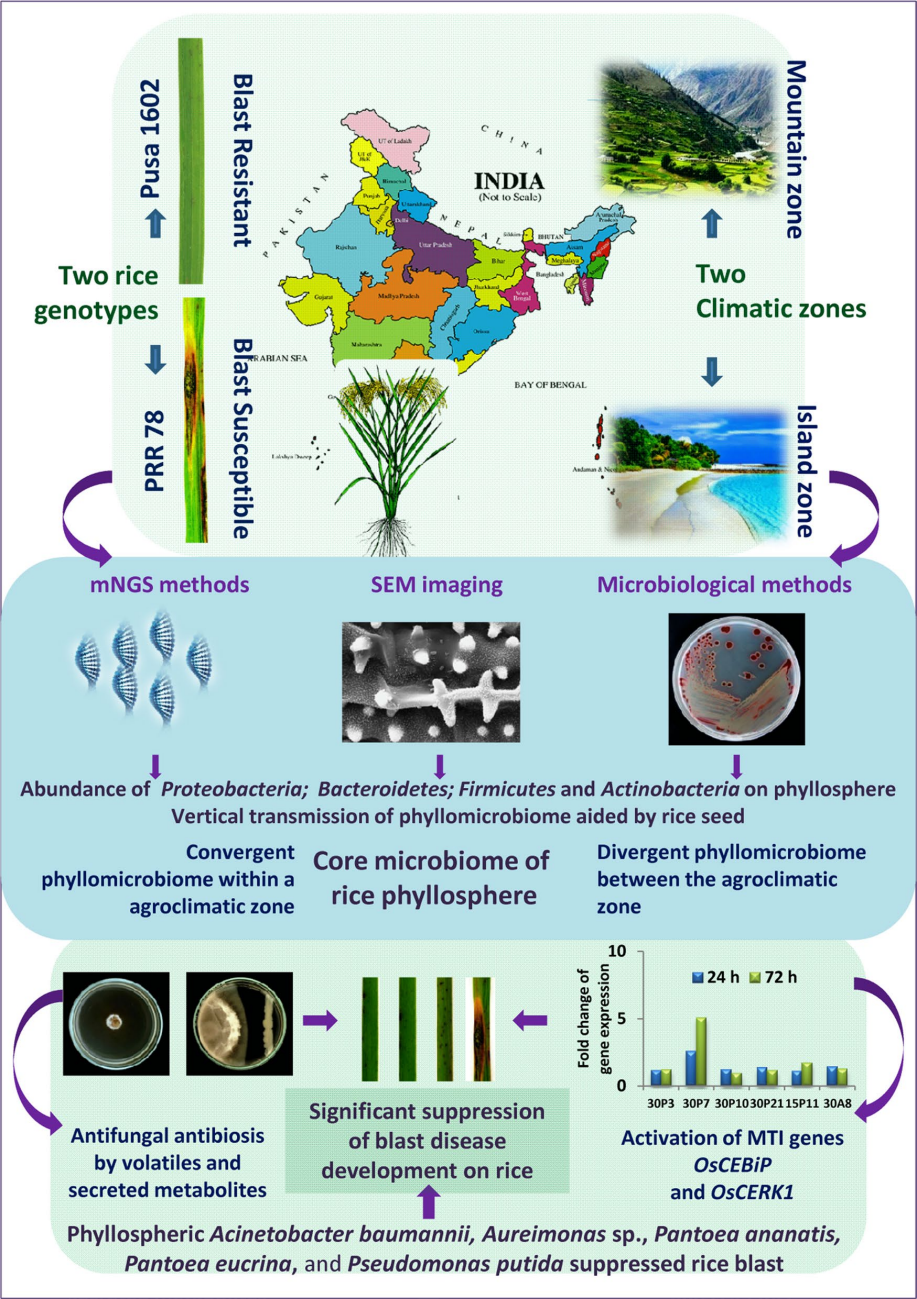

**Supplementary Information:**

The online version contains supplementary material available at 10.1186/s40793-022-00421-5.

## Background

Microbiota colonizing the plants termed plant microbiome is believed to confer metabolic flexibility and functionality to the plant genomes [1, 2]. Here the microbial communities interact dynamically among them as well as with the plant species displaying cooperative or competitive relationships with implications for the plant physiological and ecological functions.

The phyllosphere, a harsh habitat, is predicted to represent 10^9^ square kilometers harboring 10^26^ bacterial cells on a global scale [3]. The fundamental role of the phyllosphere habitat in shaping plant functional ecology is often underestimated. In the phyllosphere, the microbiome composition and function are impacted by a variety of intrinsic biotic and abiotic factors including micro and macro climatic events [4, 5]. Microbial association on phyllosphere and their complex interactions modulating plant growth, and defense against phytopathogens are reported. Furthermore, the prokaryotic diversity on the phyllosphere is large enough to play a pivotal role in plant survival [6–9], albeit neutral and commensal existence of certain microbiota [10]. Ecological factors shaping the microbiome function in the plant are reported in some cases [3, 11–13]. Nonetheless, the key drivers of phyllomicrobiome composition and their functions are not fully understood.

Qualitatively, the phyllomicrobiome is composed of non-pathogenic bacterial communities belonging to phyla such as *Proteobacteria, Firmicutes, Bacteroides,* and *Actinobacteria* [14, 15]. Bacterial genera such as *Acinetobacter, Bacillus, Citrobacter, Curtobacterium, Enterobacter, Erwinia, Frigoribacterium, Hymenobacter, Kineococcus, Methylobacterium, Pantoea, Pseudomonas,* and *Sphingomonas* are reported to colonize the phyllosphere niche [15–20]. Phyllosphere-adapted bacteria display adaptive traits such as dark pigmented cells, extracellular polymeric substances, biosurfactants, biofilms, and utilization of plant/microbial volatile compounds [21]. It is further presumed that the epiphytic bacterial communities survive on sugar photosynthates sourced from the leaf interior diffused through the cuticle [22, 23].

Phyllosphere is also a habitat for pathogenic microbes such as *Magnaporthe* and *Xanthomonas* that cause foliar diseases which are a threat to food security [24–27]. For instance, the rice blast accounts for nearly 30% loss which is enough to feed 60 million world’s human population if managed preemptively [28]. Currently, blast management depends on fungicides and host resistance; both are inadequate to combat the production losses during epidemics. Whereas the fungicides are not compatible with the environment and trade, the host resistance is non-durable owing to the emergence of new pathotypes [29]. It is further reported that the blast resistance conferred by resistance genes in rice varieties often breaks down within 3–5 years due to the preexisting virulence diversity of *M. oryzae* [30]. Therefore, there is a need for the development of a sustainable blast management strategy. Bespoke microbiome therapy is proposed as a NextGen-Crop-care strategy to ensure eco-friendly crop disease management [31]. Microbial strains with desired functions can be engineered to form synthetic microbiomes for agricultural applications [32]. However, the development of such synthetic microbiomes is often hampered by our limited understanding of the core functional microbiome. Harnessing the potential of phyllomicrobiome for the management of foliar disease like rice blast has not been attempted to date. Since the phyllosphere microbiomes have been reported to play a pivotal role in growth, development, and defense against biotic and abiotic stress, deciphering the phyllomicrobiome functions assumes significance.

With this background, the current investigation was conducted to identify the functional core-phyllomicrobiome for harnessing its potential as a bioinoculant against blast disease. We further attempted to decipher the driver(s) of phyllomicrobiome assembly using the mNGS and culturomic methods. For this purpose, phyllomicrobiome isolated from blast resistant and susceptible rice genotypes sourced from two contrasting agro-climatic zones were analyzed. The agroclimatic zones represented the mountain zone in the Himalayan region and the island zone on Andaman Island. The results indicated an association of complex microbial assemblages displaying diverse functions for microbiome-assisted rice cultivation in the future.

## Methods

### Experimental site and phyllosphere sampling

We analyzed rice phyllomicrobiome from two contrasting agroclimatic zones of India. The experimental sites were (i) blast endemic mountain-zone at Palampur, Himachal Pradesh, India [32°6′4.7"N, 76°32′39.79"E; altitude 1275 m above mean sea level (MSL); mean temperature 22–23 °C; mean rainfall 700–1000 mm; relative humidity (RH) 60.0%]; and (ii) blast non-endemic Island-zone in Port Blair, Andaman Island, India [11°38′07.0"N, 92°39′12.7"E); altitude 16 m above MSL, mean temperature 26–28 °C, mean rainfall 3060 mm; RH 80.0% (https://en.climate-data.org; www.worldweatheronline.com)]. Both experiments were conducted during cultivation seasons in August–September 2016 at Palampur and March–April 2017 at Port Blair. Blast disease susceptible PRR78 and its near-isogenic line Pusa1602 introgressed with *Pi2* gene [33] conferring complete resistance to blast were planted and grown in parallel rows with a spacing of 20 cm by following standard agronomic practices. Phyllomicrobiome were collected aseptically in sterilized falcon tubes 15 and 30 days post sowing. Thus collected samples in three replications from each location were transported to the laboratory in cool containers maintained at 4 °C ± 0.5 °C, and processed within 48 h.

### mNGS profiling of phyllomicrobiome

**Extraction of microbial community genomic DNA** Leaf (5.0 g) samples collected from the two rice genotypes in two replications were shaken with 50 ml of sterile phosphate buffer saline [PBS, g L^−1^ NaCl 8; KCl 0.2; Na_2_HPO_4_ 1.44; KH_2_PO_4_ 0.24; pH-7.4] amended with 0.1% Tween-20 (PBS-T) to dislodge the phyllomicrobiome. The phyllosphere samples were serially extracted six times in 50 ml of PBS buffer by vigorous shaking for 30 min at 250-rpm followed by vortexing for 10 s. This method is routinely practised in our lab and is efficient to dislodge all (or most) of the bacterial cells from the rice leaf surfaces. Thus obtained phyllomicrobiome suspension (300 mL) was collected aseptically in a pre-sterilized container and centrifuged at 12 K g force for 60 min at 4.0 ºC to collect the phyllomicrobiome pellets. The pellet was subjected to total microbial community DNA extraction by the CTAB method described by Moore et al. [34]. The quality and yield of microbial DNA were assessed electrophoretically, spectrophotometrically (NanoDrop 2000, Thermo Scientific, USA), and fluorometrically (Qubit dsDNA BR Assay; Thermo Fisher Scientific Inc., Qubit® 2.0).

**Preparation of mNGS libraries** The 16S rRNA gene amplicon libraries were prepared using Nextera XT Index Kit (Illumina Inc. San Diego, CA, USA). Primers (V3F: 5′-CCTACGGGNGGCWGCAG-3′ and V4R: 5′-GACTACHVGGGTATCTAATCC-3′) for the amplification of the 490-bp hyper-variable V3-V4 region of 16S rRNA gene of Eubacteria and Archaea were used. The target amplicons were generated using a fusion-primer consisting of adaptors and multiplex index sequence as per the manufacturer’s instructions (Illumina Inc. San Diego, CA, USA). The amplicon libraries were purified by 1X AMpureXP beads, checked on Agilent High Sensitivity (HS) chip on Bioanalyzer 2100, and quantified on fluorometer using Qubit dsDNA HS Assay kit (Life Technologies, California, USA). Quality passed libraries were equimolar-pooled, and then sequenced using 300 × 2 pair-end sequencing chemistry following the manufacturer’s protocols (Illumina, San Diego, CA, USA).

### Bioinformatic analysis

Initially, the sequenced raw forward-reads (R1) and reverse-reads (R2) from all samples were visualized using the FastQC version [35] to screen the quality of reads (https://www.bioinformatics.babraham.ac.uk/projects/fastqc/). The raw reads were, then, curated to remove poor quality reads using Trimmomatic v0.35 [36] with parameters to i) *remove adapter sequences,* and ii) *curate ambiguous reads (reads with unknown nucleotides “N” larger than 5%), low-quality sequences (reads with more than 10% quality threshold (QV)* < *20 Phred score)* (http://www.usadellab.org/cms/?page=trimmomatic). The final quality passed read pairs were joined using PEAR (Paired-End reAd mergeR) version 0.9.8 (https://cme.h-its.org/exelixis/web/software/pear/) with default parameters. The joined paired reads were processed for the downstream taxonomic classification; the unpaired reads were discarded. The taxonomic classification of the final high-quality reads was performed using MG-RAST v4.0 (https://www.mg-rast.org/), wherein 1) 16SrRNA featured reads were sorted using Sortme RNA, 2) sorted reads were clustered at ≥ 97% similarity using CD-HIT method, and then 3) clustered reads were taxonomically classified against SILVA SSU database (https://www.arb-silva.de/). The classified reads/taxon abundance downloaded > 100 bases and 90% similarity through best hit classification.

### Metagenome statistical analysis

Statistical Analysis of Metagenomic Profile (STAMP; V 2.9) (https://beikolab.cs.dal.ca/software/STAMP) was referred to determine microbial diversity and abundance in the phyllosphere. Welch-T-test and Post-Hoc Test at a confidence interval of ≥ 95% was followed. Further, Microbiome Analyst [37] was utilized for the determination of α-diversity, and β- diversity, as well as to identify core-phyllomicrobiome (https://www.microbiomeanalyst.ca/). For this, initially, reads were rarefied on minimum library size (18,000 reads, minimum classified read in a sample), and then total sum scaling (TSS) was applied for data normalization. α-diversity significance was calculated using the ANOVA test; Principal Coordinate Analysis (PCoA) was performed using Analysis of similarities (ANOSIM) based on the Bray–Curtis method. The biomarker features were determined through the linear discriminant analysis (LDA) combined with the effect size measurements (LDA-LEfSe) approach at significance *P* < 0.05 and LDA score > 2.0 (http://huttenhower.sph.harvard.edu/lefse/). The bacterial genera co-occurrence network was analyzed using the SparCC method with the significance of *P* < 0.05 and correlation coefficient R^2^ > 0.60 or < − 0.6 (http://github.com/scwatts/FastSpar).

### Microscopic visualization of phyllomicrobiome

**Scanning electron microscopy** Scanning electron microscopy (SEM) was adopted for visualization of rice phyllomicrobiome following the method of Bozzola [38]. For SEM, rice leaves were cut into small pieces (3 mm^2^) and fixed in 2.5% glutaraldehyde for 12 h at 4.0 °C, rinsed in phosphate buffer saline (PBS-0.1 M, pH 7.2) for 10 min. Leaves were then dehydrated through graded series of 70, 80, 90, 95, and 100% acetone and then dried with a chemical dryer.

The leaf preparations were, then, mounted on aluminium stubs using silver adhesive tape and sputter-coated with gold: palladium alloy (18 nm) for 30 min consisting of 10 cycles of three min each for uniform coating (SC 7620 Emitech sputter coater with a pressure of 10^−1^ mbar). Thus prepared leaf samples were examined and visualized under Scanning Electron Microscope (Zeiss EVO MA 10; Oxford Technologies) at 20.00 kV and magnifications ranging from 4KX to16KX. The entire leaf surface was scanned for the presence of bacterial cells and imaged.

### Culturomic analysis of phyllomicrobiome

**Isolation and characterization of the cultivable phyllomicrobiome** Another set of the leaf samples (500 mg) collected were subjected to culturomic analysis on nutrient agar [NA, gL^−1^ Peptone 5.0; Beef extract 3.0; NaCl 5.0; Agar 15.0; pH 7.0 ± 0.2] and M9 minimal media [2 mM MgSO_4_; 0.1 mM CaCl_2_; 0.3% Glucose; 1.5% Agar; 1 × M9 salts (5 × M9 salts gL^−1^ Na_2_HPO_4_.7H_2_O 64.0; KH_2_PO_4_ 15.0, NaCl 2.5; NH_4_Cl 5.0)]. Briefly, the leaf was shaken with 50 ml of sterile phosphate buffer saline amended with 0.1% tween-20 (PBS-T) for 30 min at 250 rpm followed by vortexing for 10 s. The aliquot, thus, obtained was serially diluted up to 10^–5^. Aliquots of 1.0 ml at 10^–3^, 10^–4^, and 10^–5^ from each sample were plated in nutrient agar, and M9 minimal media supplemented with redox dye 2, 3, 5 triphenyl tetrazolium chloride (50 mgL^−1^) for morphotyping of the bacterial communities. The plates in three biological and technical replication were incubated at 28 °C ± 2 °C for 72 h. The bacterial colonies that appeared were counted and morphotyped (by size, shape, color, texture, and margin). Later on, a representative colony of each morphotype was sub-cultured, purified, and frozen at − 80 °C and − 20 °C as glycerol stock (30% V/V). Species richness and the Shannon–Wiener diversity index (H) were determined.

### Molecular diversity and identification

**BOX-PCR DNA fingerprinting** Genomic DNA of the bacterial isolates was extracted by the CTAB method prescribed by Moore et al. [34]. Isolated and purified genomic DNA was quantitated and quality checked electrophoretically and spectrophotometrically (NanoDrop 2000, ThermoScientific, USA). Finally, the genomic DNA was reconstituted at 100 ng µl^−1^and used as a template in PCR. Box-PCR was performed for diversity analysis as well as to eliminate the duplicate isolates from the collection [39]. The BOX-PCR amplicon profiling specifically amplifies the non-coding conserved sequences in the bacterial genome and is considered a highly discriminatory DNA fingerprinting technique [40, 41]. Amplicons were resolved in 1.0% agarose gel at 30 V for 10–12 h and imaged (QuantityOne, BioRad Laboratories, USA). Isolates showing identical amplicon profiles were presumed to be duplicates and represented one BOX-Amplicon Group. One representative isolate from each BOX-Amplicon Group was eventually used in the downstream work.

**Species identification by 16S rRNA gene sequencing **Amplification of 16S rRNA gene was performed using primers 27F (27F: 5′-AGAGTTTGATCCTGGCTCAG-3′) and 1492R (1492R: 5′-GGTTACCTTGTTACGACTT-3′) to amplify the 1465 bp to establish identity [42, 43]. Then, the PCR amplicons resolved in 1.0% agarose gel were purified using a gel elution kit according to the manufacturer’s instructions (Promega Corporation, USA). The cycle sequencing reaction was performed using 20–30 ng of the amplicon using the ABI PRISM BigDye Terminators v3.1 cycle sequencing kit according to the manufacturer's instruction. (Applied Biosystems Foster City, CA, USA). The purified amplicons were sequenced bi-directionally to obtain maximum coverage of the sequences. The sequences were end trimmed, edited, and contig assembled using DNA-baser (http://www.dnabaser.com/). The curated sequences were, further, subjected to Basic Local Alignment Search Tool analysis (NCBI nucleotide BLAST) to establish their identity by closest match (https://www.ncbi.nlm.nih.gov/nucleotide/). All curated 16S rRNA gene sequences of bacterial species were submitted to the GenBank database and assigned accession numbers.

### Functional screening of phyllosphere bacterial communities

**Antifungal activity on *Magnaporthe oryzae*** Volatile and secretory metabolite mediated antagonistic assay of bacterial isolates was conducted on *M. oryzae* (isolate 1637) by dual culture confrontation method. The per cent inhibition of mycelial growth over mock was estimated by adopting the methods described by Sheoran et al. [42] and Munjal et al. [43]. Additionally, the fungicidal or fungistatic nature of the bacterial volatiles on *M. oryzae* was also determined. Briefly, bacterial isolates found completely inhibiting the growth of *M. oryzae* were further allowed to re-establish mycelial growth. Based on the re-growth of the mycelium, the bacterial volatile were either categorized as fungicidal or fungistatic.

The radial growth of the fungus was measured and per cent inhibition of growth over control was calculated with the help of the following formula

$$\mathrm{I}=\frac{\mathrm{C}-\mathrm{T}}{\mathrm{C}}\times 100$$

Where I = Per cent inhibition, C = Colony diameter in control, T = Colony diameter in treatment.

**Blast suppressive activity** The bacterial isolates showing antagonism to blast fungus was selected for this assay. Blast susceptible rice genotype, Pusa Basmati-1, was allowed to germinate in the presence of bacterial cells (2 × 10^7^ CFU mL^−1^) for five days. Upon germination, the transplants were, further, grown in a climate-controlled greenhouse set at a temperature of 28 °C ± 2 °C/ RH 90 ± 10% /Light/dark cycles 14/10 h. Seedlings were foliar sprayed with bacterial suspension (Phyllobacterization; 10^7^ CFU mL^−1^) and challenged with a conidial suspension of *M. oryzae *1637 (2 × 10^5^ conidia mL^−1^) three weeks post sowing according to the protocols of Rajashekara et al. [44]. Blast disease index was determined seven days post-inoculation using a 0–5 disease rating scale where 0 = no evidence of infection; 1.0 = brown specks smaller than 0.5 mm in diameter; 2.0 = brown specks of 0.5–1.0 mm in diameter; 3.0 = roundish to elliptical lesions of about 1.0–3.0 mm in diameter; 4.0 = typical spindle-shaped blast lesion, 3 mm or longer with little or no coalescence of the lesion; 5.0 = same as 4.0 but half or more leaves killed by coalescence of lesions. Plants scored 0.0–2.0 were considered resistant, 3.0 as moderately susceptible, and 4.0–5.0 were considered susceptible [45]. The disease severity was calculated using the following formula.

$$\mathrm{Disease\, severity}=\frac{\sum (\mathrm{scale}\times \mathrm{number\, of\, plants \, infected})\times 100}{\mathrm{total\, number\, of\, plants}\times \mathrm{maximum\, disease\, scale}}$$

Further, the per cent reduction in disease severity as compared to control was estimated using the following formula.

$$\mathrm{RDS}=\frac{\mathrm{C}-\mathrm{T}}{\mathrm{C}}\times 100$$

where RDS = Reduction in Disease Severity (%), C = Disease Severity in control, T = Disease Severity in treatment.

### Phyllosphere bacteria conferred immunocompetence in rice

Phyllosphere bacteria conferred immunocompetence in rice was assayed by qPCR-based transcriptional analysis. Six bacterial isolates such as *Pantoea ananatis* OsEp-Plm-30P3, *Pantoea ananatis* OsEp-Plm-30P21, *Pantoea ananatis* OsEp-AN-30A8, *Aureimonas* sp. OsEp-Plm-30P7, *Pantoea eucrina* OsEp-Plm-30P10, and *Pseudomonas putida* OsEp-Plm-15P11 showing significant blast suppression were selected for the study. Briefly, the seedlings of Pusa Basmati-1 bacterized with 2 × 10^7^ CFU mL^−1^ were sampled at 24, 48, and 72 hpi were immediately snap-frozen in liquid nitrogen (to arrest all the cellular activity) and stored instantly at -80 °C till further use.

Total RNA was isolated from the seedlings using the SV Tool RNA Isolation System according to the manufacturer's instructions (Promega, Madison, USA). The quality and quantity of RNA were assessed spectrophotometrically (NanoDrop 2000, ThermoScientific, USA) as well as in agarose gel electrophoresis. The experiment was repeated two times with three technical replications.

**Choice of defense genes** Putative defense genes, *OsCEBiP* [46], *OsCERK1* [47], *OsPAD4* [48], *OsEDS1* [49], *OsNPR1* [50], *OsPDF2.2* [51], *OsFMO1* [52, 53] and *OsPR1.1* [54] were chosen; PCR primers specific for the above defense genes are presented (Additional file 1: Tables S1–S2**)**. The qPCR experiment was conducted in Light Cycler 96 (Roche Life Science, Switzerland) using GoTaq® 1-Step RT-qPCR System; qPCR reaction conditions were as follows; one cycle of reverse transcription at 37 °C/15 min followed by reverse transcriptase inactivation step of 95 °C/10 min followed by 30 cycles of 95 °C/10 s, annealing at 58 °C/30 s and extension at 72 °C/30 s followed by three-step melting of 95 °C/10 s, 63 °C/60 s, and 97 °C/1.0 s and then final cooling at 37 °C/30 s**.** The expression levels of all eight defense genes were calculated with reference to the expression of a housekeeping gene, *OsActin*, for normalization. Then, the qPCR data were analyzed using LightCycler®96 Roche SW 1.1 software, and the mean Ct values were considered for calculation of 2^−ΔΔCT^ to estimate the fold changes in gene expression. The fold change data were interpreted as value 1.0 for no change, ≥ 1.0 for up-regulated, ≥ 2.0 represents significant up-regulation, ≤ 1.0 is down-regulation, and ≤ 0.5 for significant down-regulation.

### Statistical analyses

All the experimental data were analyzed using the data analysis tool available in MS Office Excel 2007. The data obtained were subjected to a test of significance by analysis of variance (ANOVA) at a *P* ≤ 0.05 level of significance. Further, various parameters like the standard error of the mean (SEm), standard error of the difference between two means (SEd), the critical difference (CD), and coefficient of variation (CV) were calculated. For figures and tables, the values are represented as the mean of all biological and technical replicates.

For the qPCR-data analysis, the fold change values determined for the defense genes were imported into the GraphPad Prism program (https://www.graphpad.com/scientific-software/prism) and two way ANOVA was performed using the Bonferroni Post-hoc test for determining the statistical significance at *P ≤ 0.05, **P = 0.001 and ***P = 0.0001.

## Results

### Metagenome read statistics and bacterial diversity indices

**Fig. 1:**
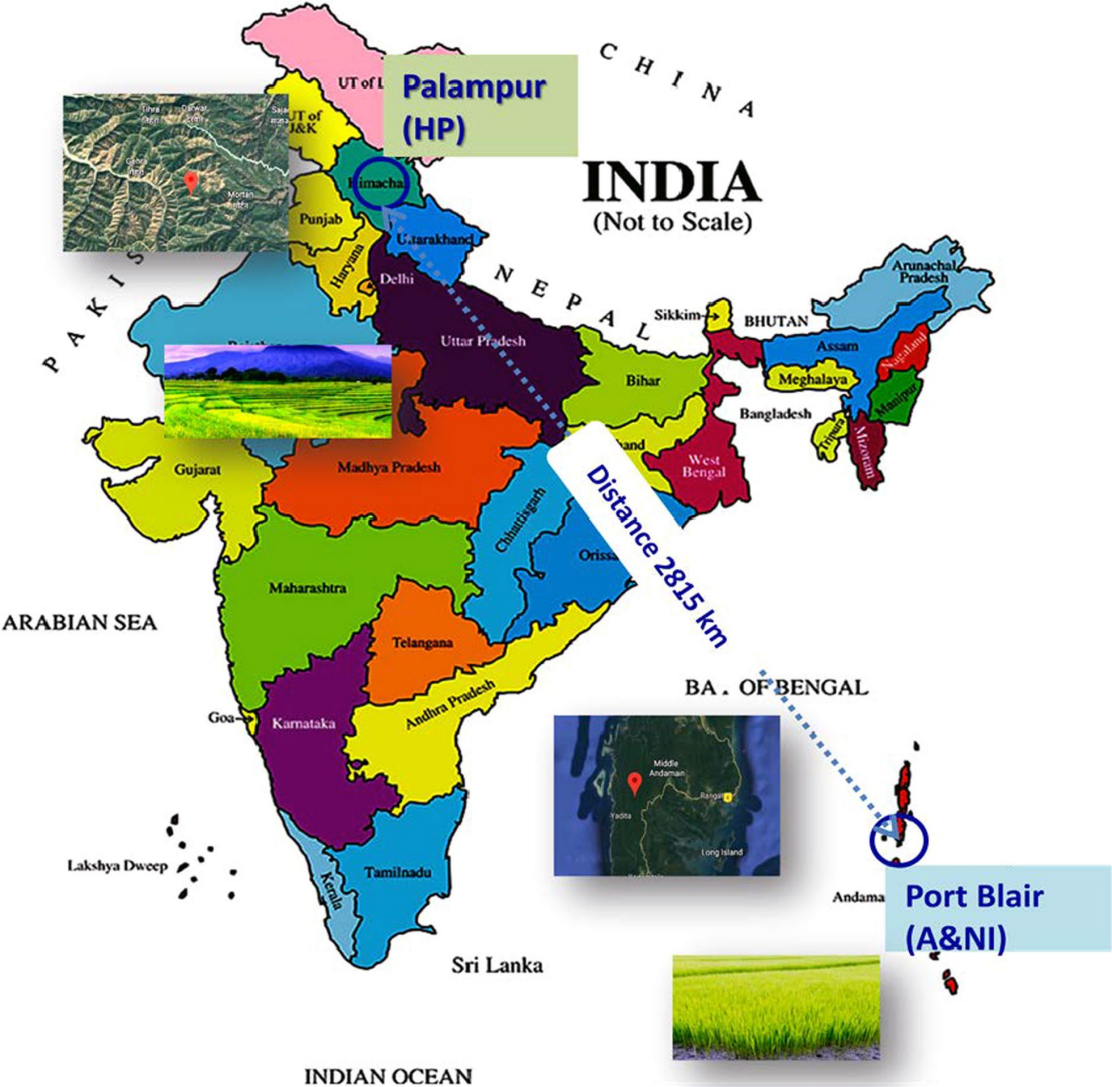
Experimental sites at Mountain and Island agroclimatic zones of India. Satellite images of experimental sites, Palampur in Himachal Pradesh, India, and Port Blair, Andaman & Nicobar Island are shown inserted. Experiments were conducted during the rice cultivation season in both locations

**Fig. 2:**
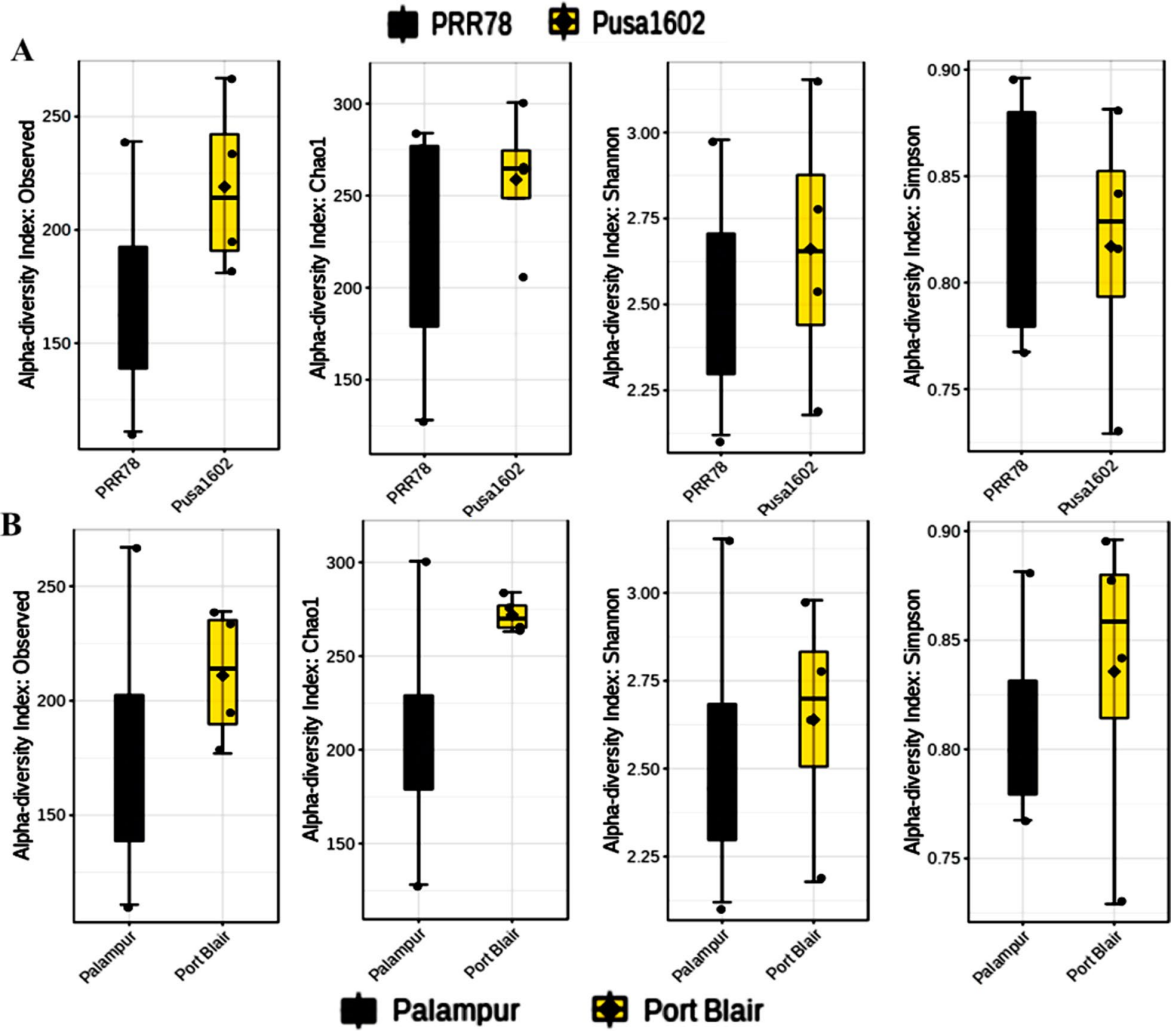
Alpha diversity Indices of rice phyllomicrobiome; Comparative diversity indices for **A** Two genotypes, PRR78 -a blast disease susceptible genotype, and Pusa1602 -a blast disease-resistant NIL genotype; **B** Two locations, Palampur –Mountain zone, and Port Blair –Island zone; ANOVA test was performed among the groups

Phyllomicrobiome profiles of PRR78 (Blast susceptible) and Pusa1602 (Blast resistant) grown in contrasting agro-climatic zones were analyzed by mNGS and culturomic methods (Fig. 1). A total of eight samples were generated and subjected to comparative microbiome analysis (Fig. 1; Additional file 1: Table S3). The alpha diversity indices of phyllosphere microbial diversity determined using the mNGS data are furnished in Table 1. While the Shannon diversity index ranged from 2.12–3.15, the Simpson and Chao1 are in the range of 0.729–0.896 and 128.11- 300.61, respectively. The observed species was in the range of 111.0–267.0. The maximum diversity and OTUs were observed in the Island zone rice phyllosphere (Fig. 2; Table 1).

**Table 1:** α-diversity of rice phyllomicrobiome representing contrasting agroclimatic zones

Location	Genotype	Samples	Shannon	Chao1	Simpson	Observed
			Value	Value	Value	Value
Island Zone	PRR78	PRR78-ANI1-R1	2.615	274.65	0.875	177
Island Zone	PRR78	PRR78-ANI1-R2	2.979	284.00	0.896	239
Mountain Zone	PRR78	PRR78-Plm1-R1	2.120	128.11	0.783	111
Mountain Zone	PRR78	PRR78-Plm2-R2	2.356	195.83	0.767	148
Island Zone	Pusa1602	Pusa1602-ANI1-R1	2.178	263.09	0.729	194
Island Zone	Pusa1602	Pusa1602-ANI2-R2	2.784	265.88	0.843	234
Mountain Zone	Pusa1602	Pusa1602-Plm1-R1	2.527	205.24	0.815	181
Mountain Zone	Pusa1602	Pusa1602-Plm2-R2	3.154	300.61	0.881	267

Microbiome Analyst [37] was utilized for the determination of α-diversity

### Principal component analysis (PCoA)

**Fig. 3:**
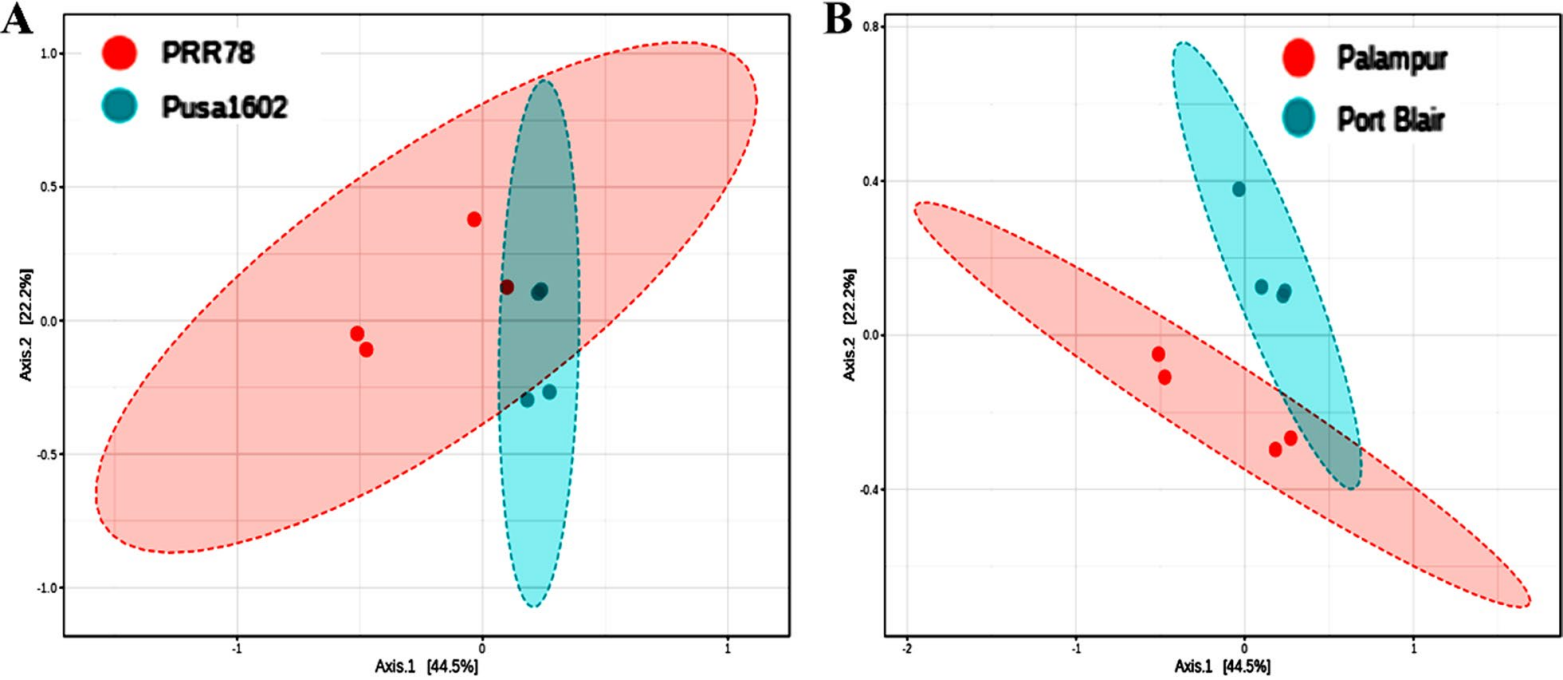
Principal Coordinate analysis (PCoA) based Bray–Curtis distance dissimilarity matrices with ANalysis of SIMilarity (ANOSIM) was applied for beta diversity analysis or rice phyllosphere microbiome between; **A** two genotypes, PRR78 and Pusa1602; **B** two locations, Palampur and Port Blair

PCoA of metagenome reads of rice genotypes, PRR78, and Pusa1602 by Bray–Curtis and ANOSIM revealed converging and shared microbiome assemblage on rice genotypes when grown in the same agroclimatic zone. The same genotype, either PRR78 or Pusa1602, showed diverging microbiome composition when grown in another agroclimatic zone, either Mountain or Island zone (Fig. 3).

### Linear discriminant analysis (LDA) effect size (LEfSe) analysis

The LDA-LEfSe score calculated at a 2.0 significance level revealed microbial biomarker profiles for rice genotypes and agroclimatic zones. The result revealed a total of 10 and 2 biomarkers for Pusa1602 and PRR78 respectively. While *Klebsiella* and *Exiguobacterium* were biomarkers for PRR78, genera such as *Methylobacterium, Janibacter, Frankia, Macrococcus, Leptolyngbya, Shigella, Pseudacidovorax, Anoxybacillus,* and *Cellulosimicrobium* were biomarkers for Pusa1602. For the geographical location, a total of 15 and 16 biomarkers for the mountain zone and the island zone were discovered, respectively. While the genera such as *Pantoea, Arthrobacter, Acidovorax, Erwinia, Microbacterium, Shewanella, Acinetobacter, Sphingobacterium, Pseudoalteromonas, Herbaspirillum, Psychrobacter, Candidatus-Koribacter, Mesorhizobium, Variovarax,* and *Roseateles* were found as biomarkers for mountain zone, *Lysinibacillus, Alkaliphilus, Cylindrospermum, Enterococcus, Bifidobacterium, Arthrospira, Leptolyngbya, Candidatus-Aquiluna, Agromyces, Lactobacillus, Leifsonia, Clostridium, Streptomyces, Bacillus,* and *Curtobacterium* were identified as a biomarker for the island zone (Additional file 2: Fig. S1).

### SparCC network of variety and location

Network analysis showed the positive (cooperative) and negative (competitive) interactions within the phyllomicrobiome members on the phyllosphere. In agroclimatic zones and rice genotypes, as many as 68 bacterial genera were predicted to display complex interactions among themselves on the phyllosphere (Additional file 1: Table S4; Additional file 2: Fig. S2). Network analysis showed 128 & 127 cooperative and 104 & 108 competitive interactions on the rice genotypes and climatic zones, respectively.

### Comparative microbiome analysis of rice genotypes

**Fig. 4:**
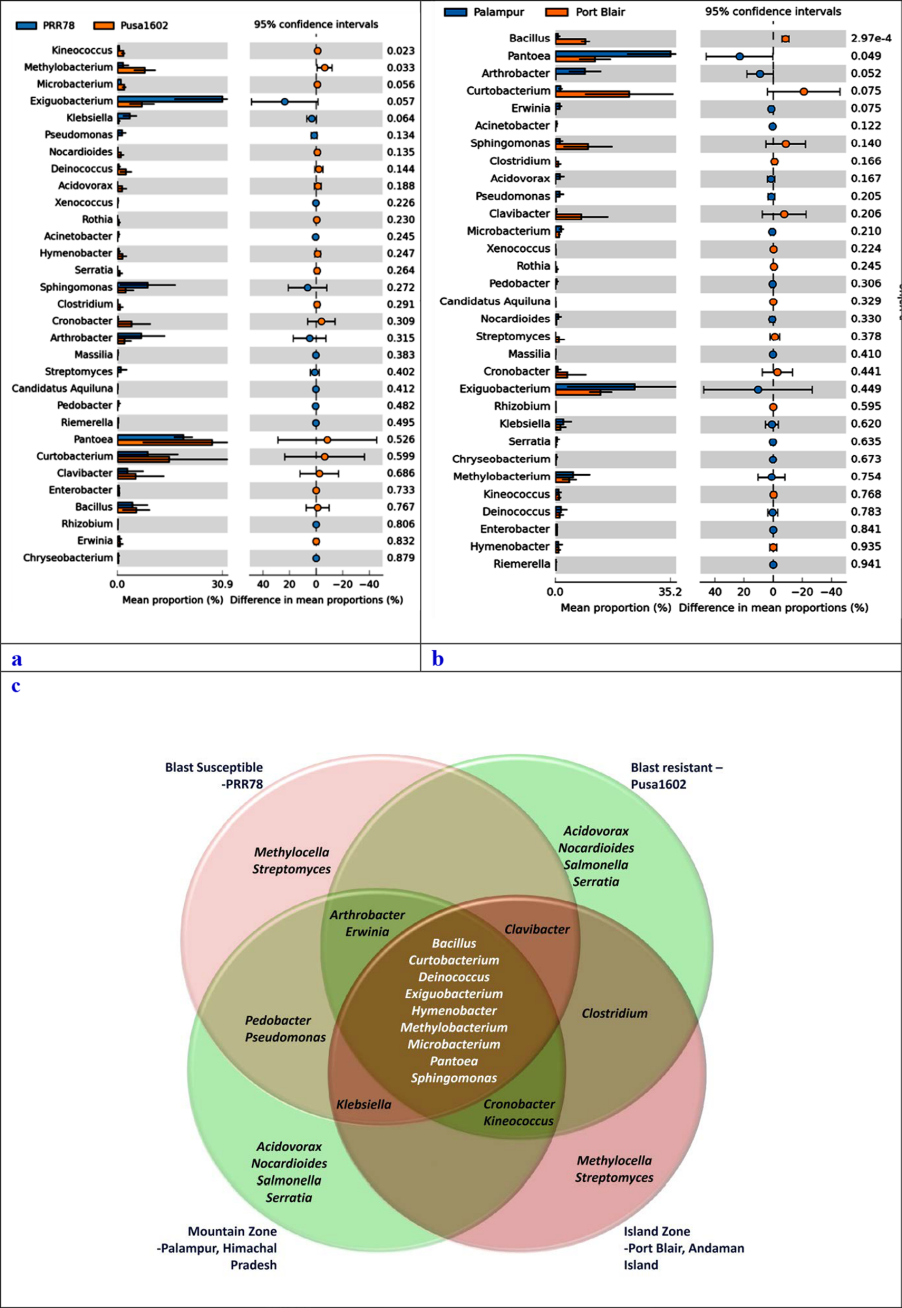
Extended error bar plots for the top 31 microbiota at the genus level; Extended error bar plots for the top 31 microbiota at the genus level using statistics Welch-*t*-test with two-sided at confidence intervals of ≥ 95%. **a** Extended error bar plots for the top microbiota at the Genus level for two genotypes; **b** Extended error bar plots for the top microbiota at the Genus level for two climatic zones; Note: Sorted by significance in ascending order, mean proportion and their differences for phyllosphere microbiota are shown; Genus *Exiguobacterium*, *Sphingomonas*, *Klebsiella, Pseudomonas*, and *Arthrobacter* in PRR78 were significantly higher in abundance than that in Pusa1602; Genus *Methylobacterium, Cronobacter, Pantoea, Curtobacterium,* and *Clavibacter* in Pusa1602 were significantly higher in abundance than that in PRR78. Genus *Pantoea, Arthrobacter, Exiguobacterium*, *Klebsiella,* and *Methylobacterium* in the Mountain zone at Palampur were significantly higher in abundance than that in the Island zone at Port Blair; Genus *Curtobacterium, Bacillus, Sphingomonas*, *Clavibacter,* and *Cronobacter* in the Island zone at Port Blair were significantly higher in abundance than that in the Mountain zone at Palampur; **c** Venn diagram showing the distribution pattern of bacterial genera on rice genotypes in two climatic zones; Note: *Bacillus, Curtobacterium, Deinococcus, Exiguobacterium, Hymenobacter, Methylobacterium, Microbacterium, Pantoea,* and *Sphingomonas *were found on both the genotypes in two agroclimatic zones

Comparative microbiome analysis of rice genotypes revealed the dominance of Proteobacteria, Firmicutes, and Actinobacteria in both the rice genotypes. A total of 11 phyla such as Deinococcus-Thermus, Aquificae, Gemmantimonadetes, Chloroflexi, Acidobacteria, Planctomycetes, Verucomicrobia, Actinobacteria, Proteobacteria, Bacteroidetes, and Nitrospirae were found over-represented on Pusa1602. On the other hand, only three phyla such as Firmicutes, Fusobacteria, and Cyanobacteria were predominated on PRR78 (Additional file 2: Fig. S3). Genus level annotations showed *Pantoea* followed by *Curtobacterium, Methylobacterium, Exiguobacterium*, and *Bacillus* on Pusa1602; PRR78 showed the dominance of *Exiguobacterium* followed by *Pantoea*, *Sphingomonas*, *Curtobacterium*, and *Arthrobacter* (Table 2; Fig. 4a–c; Additional file 2: Fig. S3.).

**Table 2:**
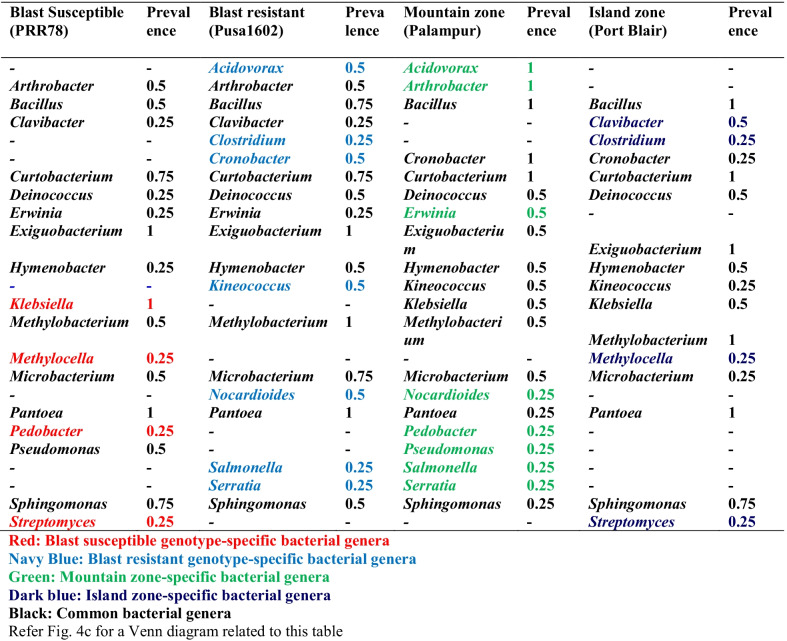
Genotype or climate zone-specific and common bacterial communities associated with phyllosphere of rice

### Comparative microbiome analysis of agroclimatic zones

**Fig. 5:**
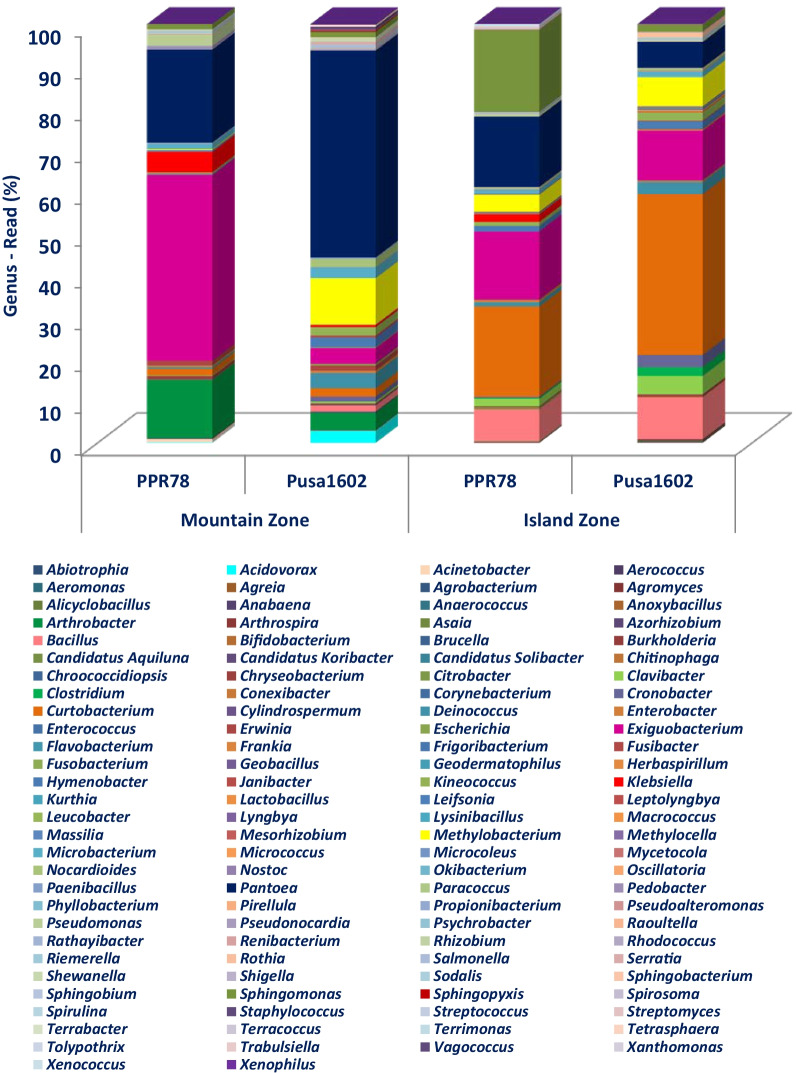
Relative abundance of bacterial communities on rice phyllosphere in two contrasting agroclimatic zones of India

Comparative phyllomicrobiome analysis of rice genotypes grown in mountain and island zones revealed the dominance of Proteobacteria, Firmicutes, and Actinobacteria (Additional file 2: Figs. S4, S5). While Actinobacteria, Aquificae, Chloroflexi, Cyanobacteria, Nitrospirae, Planctomycetes, and Verucomicrobia were found in the island zone, the mountain zone revealed the dominance of Acidobacteria, Bacteroidetes Deinococcus-Thermus, Gemmantimonadetes, Firmicutes, Fusobacteria, and Proteobacteria (Additional file 2: Figs. S4, S5). Bacterial communities observed on the phyllosphere at various taxonomic hierarchies such as class, order, and family are presented in Additional file 2: Fig. S4 and S5. At the genera level *Bacillus, Curtobacterium, Exiguobacterium, Pantoea,* & *Sphingomonas* on the Island zone, and *Arthrobacter, Exiguobacterium, Methylobacterium, & Pantoea* in the mountain zone were recorded (Table 2; Figs. 4a–c, 5; Additional file 2: Fig. S6).

### Core microbiome analysis

The bacterial taxa can be considered a member of "core microbiota" if it is "consistently" associated with all genotypes of a particular species. All other bacterial species may belong to "satellite microbiota" members. Core microbiome analysis of rice phyllosphere showed several bacterial genera with a maximum prevalence of *Pantoea, Klebsiella, Methylobacterium,* and *Exiguobacterium*. For agroclimatic zones, the core phyllomicrobiome showed a high representation of *Pantoea, Microbacterium, Exiguobacterium,* and *Arthrobacter* in the mountain zone; the island zone showed a core microbiome consisting of *Pantoea, Methylobacterium, Exiguobacterium, Curtobacterium*, and *Bacillus* (Table 3).

**Table 3:** Core phyllomicrobiome of rice genotypes grown in two contrasting climate zones

Core phyllomicrobiome	Prevalence
*Acidovorax*	0.25
*Arthrobacter*	0.5
*Bacillus*	0.625
*Clavibacter*	0.25
*Clostridium*	0.125
*Cronobacter*	0.25
*Curtobacterium*	0.75
*Deinococcus*	0.375
*Erwinia*	0.25
*Exiguobacterium*	1.0
*Hymenobacter*	0.375
*Kineococcus*	0.25
*Klebsiella*	0.5
*Methylobacterium*	0.75
*Methylocella*	0.125
*Microbacterium*	0.625
*Nocardioides*	0.25
*Pantoea*	1.0
*Pedobacter*	0.125
*Pseudomonas*	0.25
*Salmonella*	0.125
*Serratia*	0.125
*Sphingomonas*	0.625
*Streptomyces*	0.125

Microbiome Analyst [37] was utilized for the determination of core phyllomicrobiome

### SEM imaging and culturomic analysis of phyllomicrobiome

**Fig. 6:**
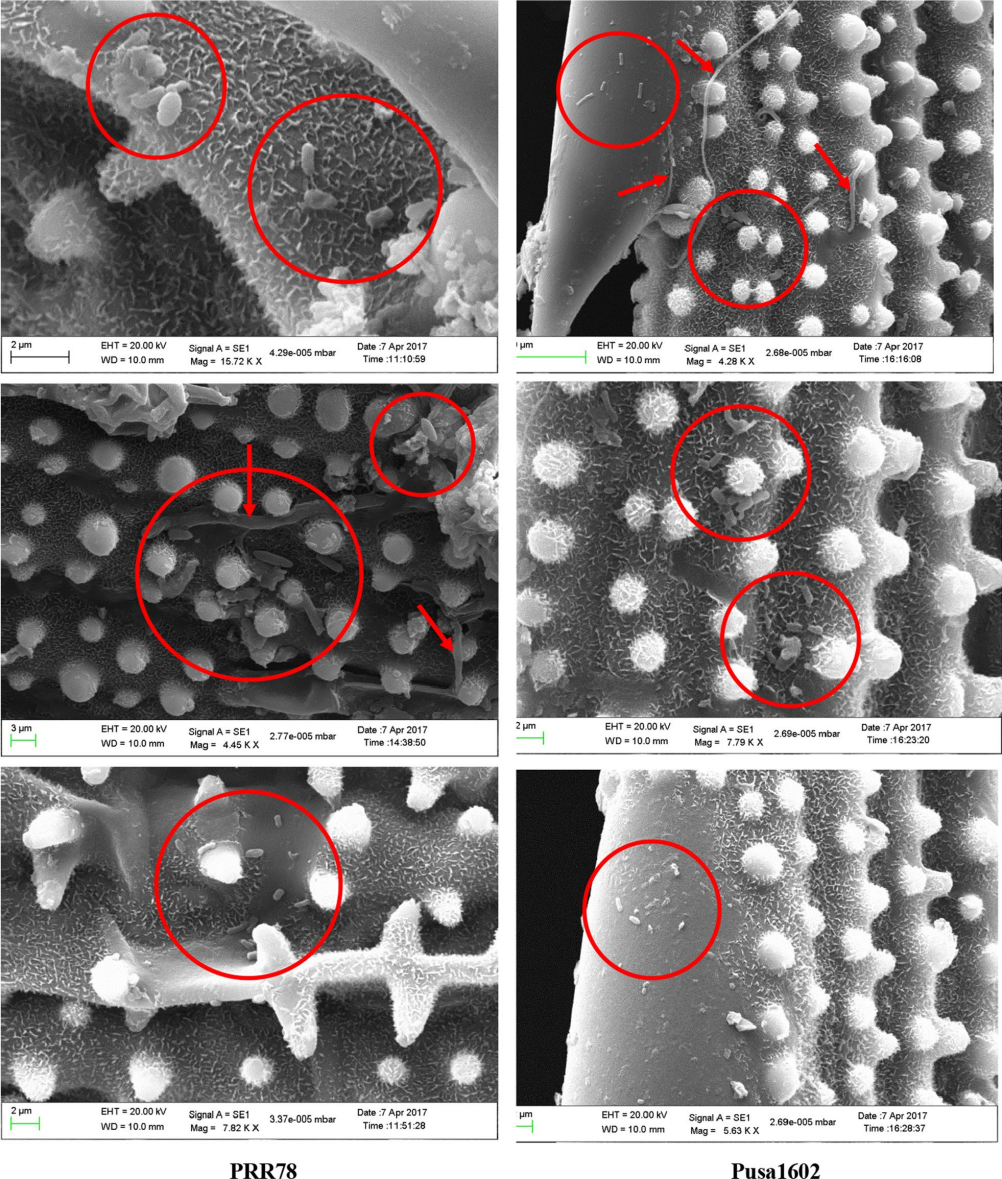
SEM images of rice phyllosphere with bacterial and fungal cells/mycelium on the surface. Red circles are indicating the bacterial cells/aggregates. The red arrow mark is indicating the presence of eukaryotic fungal hyphae

The SEM imaging of the rice leaf surface revealed the physical presence of bacterial cell aggregates of 5–8 cells, and unevenly distributed solitary bacterial cells on the phyllosphere of rice genotypes. The Eukaryotic cells and hyphal fragments were also found scattered among the prokaryotic cells (Fig. 6). The blast susceptible genotype (3.127–4.313 CFU g^−1^) recorded a marginally more epiphytic bacterial population as compared to the resistant genotype (2.945–3.317 CFU g^−1^) in both locations (Additional file 1: Tables S5, S6). Similarly, a relatively more bacterial count and diversity were observed on 30 days old phyllosphere (45 morphotypes) when compared to 15 days (33 morphotypes) (Table 4). BOX-PCR amplicon profiling of all 78 morphotypes was clustered into 59 distinct BOX Amplicon Groups. Isolates such as OsEp-Plm-15P4, OsEp-Plm-15P8, OsEp-Plm-15P9, OsEp-Plm-15P10, OsEp-Plm-15P13, and OsEp-Plm-15P15 from mountain zone, and OsEp-AN-15A10, OsEp-AN-15A11, OsEp-AN-15A17, and OsEp-AN-15A18 shared all intergenic amplicons (Additional file 2: Fig. S7). Isolates sharing all amplicon profiles were considered genetically identical duplicates.

**Table 4:** Diversity indices of cultured-phyllosphere bacterial communities representing three geographical locations

Rice phyllosphere	Parameters	*Age of plantlets			
		15		30	
		Pusa1602	PRR78	Pusa1602	PRR78
Palampur	Shannon Wiener diversity index	1.30	1.80	1.80	1.58
	Species richness	10.0	10.0	15.0	17.0
Port Blair	Shannon Wiener diversity index	1.12	1.40	1.40	1.34
	Species richness	12.0	9.0	17.0	17.0

^*^Days post transplanting

Species identification by 16S rRNA gene sequencing showed the high-frequency occurrence of *Acidovorax* (3), *Acinetobacter* (6), *Aureimonas* (2), *Curtobacterium* (5)*, Enterobacter* (6)*, Exiguobacterium* (4)*, Microbacterium* (2)*, Pantoea* (16)*, Pseudomonas* (5) and *Sphingomonas* (7) on rice phyllosphere (Additional file 2: Figure S8; Additional file 1: Table S7). Six bacterial isolates from the mountain zone and four from the island zone (represented by OsEp-Plm-15P9 for the mountain and OsEp-AN-15A10 for the island) shared all intergenic amplicons (genetically identical isolates) were identified as *Pantoea ananatis.*

### Culturomic validation of mNGS classification

A total of 59 bacterial species belonging to 14 bacterial genera such as *Acidovorax, Acinetobacter, Agrobacterium, Aureimonas, Curtobacterium, Enterobacter, Enterococcus, Erwinia, Exiguobacterium, Microbacterium, Micrococcus, Pantoea, Pseudomonas,* and *Sphingomonas* were cultured, isolated, and preserved (Additional file 2: Fig. S9a–m). The cultured bacterial genera were all found among the mapped reads in the mNGS data. Further, comparative analysis confirmed the occurrence of *Acinetobacter, Curtobacterium, Enterobacter, Exiguobacterium, Pantoea, Pseudomonas,* and *Sphingomonas* in Mountain and Island agroclimatic zones in both the mNGS and culturomic approaches (Data not shown). Co-occurrence of *Acinetobacter, Curtobacterium, Enterobacter, Exiguobacterium, Pantoea, Pseudomonas,* and *Sphingomonas* on both the rice genotypes in the contrasting climate zone was also observed (data not shown).

## Activity screening for identification of functional core phyllomicrobiome

**Screening for antifungal activity** Among the 59 bacteria evaluated for antifungal activity, 14 isolates (23.7%) representing *Acinetobacter, Erwinia, Exiguobacterium, Pantoea,* and *Pseudomonas* showed over 40.0% inhibition of mycelial growth by their secreted metabolites (Table 5; Additional file 2: Fig. S10). A total of 15 isolates (25.4%) representing *Acinetobacter, Aureimonas, Pantoea,* and *Pseudomonas* inhibited the growth of *M. oryzae* by volatile organic compounds (Table 5; Additional file 2: Fig. S11). Further, the BVCs of five bacterial isolates were found to show fungicidal activity while the remaining ten were fungistatic on *M. oryzae* (Additional file 1: Table S6; Additional file 2: Fig. S12).

**Table 5:** Antifungal antagonistic activity displayed by bacterial communities associated with phyllomicrobiome on *Magnaporthe oryzae*

Genus	Bacterial isolate (*Closet Match)	*Sequence length (bp)	*GenBank Accession	Geographical Zone	Mycelial Inhibition (%)	
					BVC	SC
***Acidovorax***	*Acidovorax avenae* OsEp-Plm-30P1	1433	MT367817	Mountain zone	34.3	3.7
	*Acidovorax avenae* OsEp-Plm-30P23	1378	MT367833	Mountain zone	27.9	12.0
	*Acidovorax avenae* OsEp-Plm-30P6	1396	MT367820	Mountain zone	29.3	4.6
***Acinetobacter***	*Acinetobacter baumannii* OsEp-Plm-30P11	1430	MT367824	Mountain zone	100.0	39.8
	*Acinetobacter baumannii* OsEp-Plm-30P17	1401	MT367827	Mountain zone	100.0	50.9
	*Acinetobacter junii* OsEp-AN-30A17	1386	MT367859	Island zone	52.9	7.4
	*Acinetobacter soli* OsEp-Plm-30P2	1419	MT394056	Mountain zone	32.9	39.8
	*Acinetobacter soli* OsEp-Plm-30P4	1429	MT367819	Mountain zone	100.0	42.6
	*Acinetobacter soli*OsEp-Plm-30P22	1417	MT367832	Mountain zone	32.1	34.3
***Agrobacterium***	*Agrobacterium larrymoorei* OsEp-Plm-30P19	1359	MT367829	Mountain zone	46.4	5.6
***Aureimonas***	*Aureimonas phyllosphaerae* OsEp-AN-30A11	1390	MT367855	Island zone	33.6	6.5
	*Aureimonas* sp.OsEp-Plm-30P7	1369	MT367821	Mountain zone	100.0	4.6
***Curtobacterium***	*Curtobacterium albidum* OsEp-Plm-15P1	1391	MT367807	Mountain zone	32.1	1.9
	*Curtobacterium albidum* OsEp-Plm-30P20	1401	MT367830	Mountain zone	57.9	7.4
	*Curtobacterium citreum* OsEp-AN-30A1	1395	MT367846	Island zone	40.0	10.2
	*Curtobacterium luteum* OsEp-Plm-30P9	1393	MT367822	Mountain zone	39.3	13.9
	*Curtobacterium luteum* OsEp-Plm-15P7	1390	MT367812	Mountain zone	60.0	4.6
***Enterobacter***	*Enterobacter asburiae* OsEp-AN-30A22	1406	MT367864	Island zone	23.6	6.5
	*Enterobacter asburiae* OsEp-Plm-30P16	1410	MT367826	Mountain zone	41.4	35.2
	*Enterobacter cloacae* OsEp-AN-15A7	1409	MT367840	Island zone	0.0	7.4
	*Enterobacter cloacae* OsEp-Plm-30P18	1425	MT367828	Mountain zone	18.6	23.2
	*Enterobacter mori* OsEp-AN-30A20	1409	MT367862	Island zone	25.7	9.3
	*Enterobacter sichuanensis* OsEp-AN-15A12	1404	MT367844	Island zone	41.4	5.6
***Erwinia***	*Erwinia tasmaniensis* OsEp-AN-15A5	1412	MT367838	Island zone	56.4	54.6
***Exiguobacterium***	*Exiguobacterium acetylicum* OsEp-Plm-15P3	1438	MT367809	Mountain zone	54.3	1.9
	*Exiguobacterium indicum* OsEp-AN-30A4	1413	MT367849	Island zone	63.6	46.3
	*Exiguobacterium indicum* OsEp-AN-30A6	1430	MT367851	Island zone	32.1	14.8
	*Exiguobacterium indicum* OsEp-Plm-30P14	1431	MT367825	Mountain zone	24.3	3.7
***Microbacterium***	*Microbacterium* sp. OsEp-AN-15A2	1387	MT367835	Island zone	0.0	13.9
	*Microbacterium testaceum* OsEp-AN-30A2	1409	MT367847	Island zone	47.1	38.9
***Micrococcus***	*Micrococcus luteus* OsEp-AN-15A1	1400	MT367834	Island zone	0.0	12.0
***Pantoea***	*Pantoea agglomerans* OsEp-AN-15A8	1418	MT367841	Island zone	69.3	7.4
	*Pantoea agglomerans* OsEp-AN-30A14	1408	MT367857	Island zone	100.0	42.6
	*Pantoea agglomerans* OsEp-AN-30A21	1413	MT367863	Island zone	40.0	10.2
	*Pantoea ananatis* OsEp-AN-15A10	1401	MT367843	Island zone	81.4	50.0
	*Pantoea ananatis* OsEp-AN-30A19	1408	MT367861	Island zone	30.7	7.4
	*Pantoea ananatis* OsEp-AN-30A5	1402	MT367850	Island zone	100.0	4.6
	*Pantoea ananatis* OsEp-AN-30A8	1403	MT367852	Island zone	100.0	7.4
	*Pantoea ananatis* OsEp-Plm-15P9	1410	MT367813	Mountain zone	100.0	34.3
	*Pantoea ananatis* OsEp-Plm-30P21	1405	MT367831	Mountain zone	100.0	50.0
	*Pantoea ananatis* OsEp-Plm-30P3	1419	MT367818	Mountain zone	74.3	50.0
	*Pantoea dispersa* OsEp-AN-30A18	1412	MT367860	Island zone	100.0	48.2
	*Pantoea eucrina* OsEp-AN-15A4	1409	MT367837	Island zone	100.0	50.0
	*Pantoea eucrina* OsEp-Plm-15P14	1421	MT367816	Mountain zone	100.0	52.8
	*Pantoea* eucrina OsEp-Plm-30P10	1414	MT367823	Mountain zone	100.0	47.2
	*Pantoea* sp. OsEp-AN-15A15	1400	MT367845	Island zone	57.1	49.1
	*Pantoea* sp. OsEp-AN-15A9	1402	MT367842	Island zone	0.0	3.7
***Pseudomonas***	*Pseudomonas oryzihabitans* OsEp-Plm-15P6	1398	MT367811	Mountain zone	56.4	51.9
	*Pseudomonas parafulva* OsEp-Plm-15P12	1407	MT367815	Mountain zone	100.0	38.9
	*Pseudomonas psychrotolerans* OsEp-AN-15A6	1383	MT367839	Island zone	38.6	36.1
	*Pseudomonas psychrotolerans* OsEp-AN-30A13	1396	MT367856	Island zone	57.1	26.9
	*Pseudomonas putida* OsEp-Plm-15P11	1401	MT367814	Mountain zone	100.0	19.4
***Sphingomonas***	*Sphingomonas paucimobilis* OsEp-AN-15A3	1390	MT367836	Island zone	4.3	13.0
	*Sphingomonas paucimobilis* OsEp-AN-30A9	1377	MT367853	Island zone	61.4	22.2
	*Sphingomonas pseudosanguinis* OsEp-AN-30A10	1378	MT367854	Island zone	59.3	24.1
	*Sphingomonas pseudosanguinis* OsEp-Plm-15P2	1389	MT367808	Mountain zone	79.3	15.7
	*Sphingomonas* sp. OsEp-AN-30A15	1362	MT367858	Island zone	60.7	4.6
	*Sphingomonas* sp. OsEp-Plm-15P5	1378	MT367810	Mountain zone	58.6	3.7
	*Sphingomonas yabuuchiae* OsEp-AN-30A3	1362	MT367848	Island zone	35.7	6.5
**Mock**	**–**	**–**	**–**	Both zones	0.0	0.0
C.D					10.93	3.79
SE(m)					3.79	5.37
SE(d)					5.37	10.93
C.V. (%)					13.56	12.75
F (calc.)					110.82	110.82

^*^16S rRNA gene sequences as accessed in https://blast.ncbi.nlm.nih.gov/Blast.cgi

**Fig. 7:**
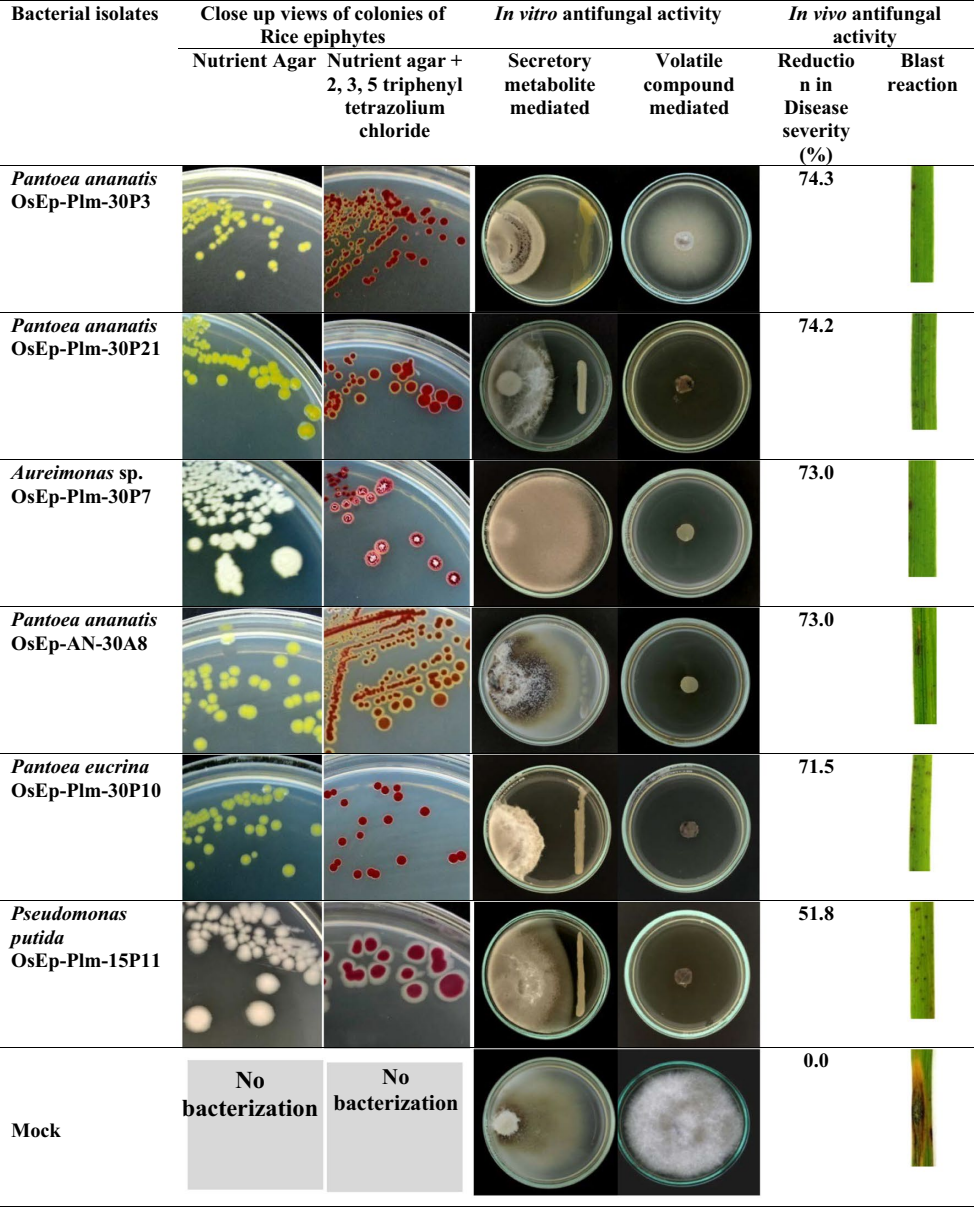
Secreted metabolite and volatile mediated antifungal activity of phyllomicrobiome bacterial communities on *Magnaporthe oryzae* and suppression of rice blast disease upon phyllobacterization. Note: Six bacterial isolates that displayed more than 50% blast suppression are shown here; refer to Additional file 2: Figs. S10–S12 for results of all bacterial isolates

**Screening for blast suppression** Twenty bacterial isolates *Pantoea* (12), *Pseudomonas* (2), *Acinetobacter* (3), *Aureimonas* (1), *Erwinia* (1), and *Exiguobacterium* (1) selected based on antifungal antibiosis were found to suppress rice blast disease. A significant reduction in blast severity was shown by *Pantoea ananatis* OsEp-Plm-30P3 (74.3%), *Pantoea ananatis* OsEp-Plm-30P21 (74.2%), *Pantoea ananatis* OsEp-AN-30A8 (73.0%.), *Aureimonas* sp.OsEp-Plm-30P7 (73.0%), *Pantoea eucrina* OsEp-Plm-30P10 (71.5%), *Pseudomonas putida* OsEp-Plm-15P11 (51.8%), *Pantoea ananatis* OsEp-Plm-15P9 (49.7%), and *Acinetobacter baumannii* OsEp-Plm-30P11 (47.3%) (Table 6; Fig. 7; Additional file 2: Fig. S13).

**Table 6:** Blast suppressive potential showed by phyllosphere bacterial genera on rice

Genus	Bacterial isolates	*Blast disease suppression		
		*Severity Score	*Severity Reduction (%)	
*Acinetobacter*	*Acinetobacter baumannii* OsEp-Plm-30P11	26.8		47.3
	*Acinetobacter baumannii* OsEp-Plm-30P17	28.6		43.7
	*Acinetobacter soli* OsEp-Plm-30P4	33.3		34.5
*Aureimonas*	*Aureimonas* sp. OsEp-Plm-30P7	13.7		73.0
*Erwinia*	*Erwinia tasmaniensis* OsEp-AN-15A5	33.5		34.2
*Exiguobacterium*	*Exiguobacterium indicum* OsEp-AN-30A4	33.0		35.0
*Pantoea*	*Pantoea agglomerans* OsEp-AN-30A14	29.7		41.5
	*Pantoea ananatis* OsEp-Plm-30P3	13.1		74.3
	*Pantoea ananatis* OsEp-Plm-30P21	13.1		74.2
	*Pantoea ananatis* OsEp-AN-30A8	13.7		73.0
	*Pantoea ananatis* OsEp-Plm-15P9	25.6		49.7
	*Pantoea ananatis* OsEp-AN-15A10	27.2		46.6
	*Pantoea ananatis* OsEp-AN-30A5	30.4		40.2
	*Pantoea dispersa* OsEp-AN-30A18	31.2		38.5
	*Pantoea eucrina* OsEp-Plm-30P10	14.5		71.5
	*Pantoea eucrina* OsEp-AN-15A4	27.1		46.7
	*Pantoea eucrina* OsEp-Plm-15P14	28.0		45.0
	*Pantoea* sp. OsEp-AN-15A15	27.2		46.5
*Pseudomonas*	*Pseudomonas parafulva* OsEp-Plm-15P12	32.5		36.2
	*Pseudomonas putida* OsEp-Plm-15P11	24.5		51.8
Pathogen-Check	Control	50.8		0.0
Fungicide-Check	Tricyclazole control	8.33		83.6

^*^Average of three repeat trials each with five replications

### Phyllosphere bacteria conferred immunocompetence in rice

**Fig. 8:**
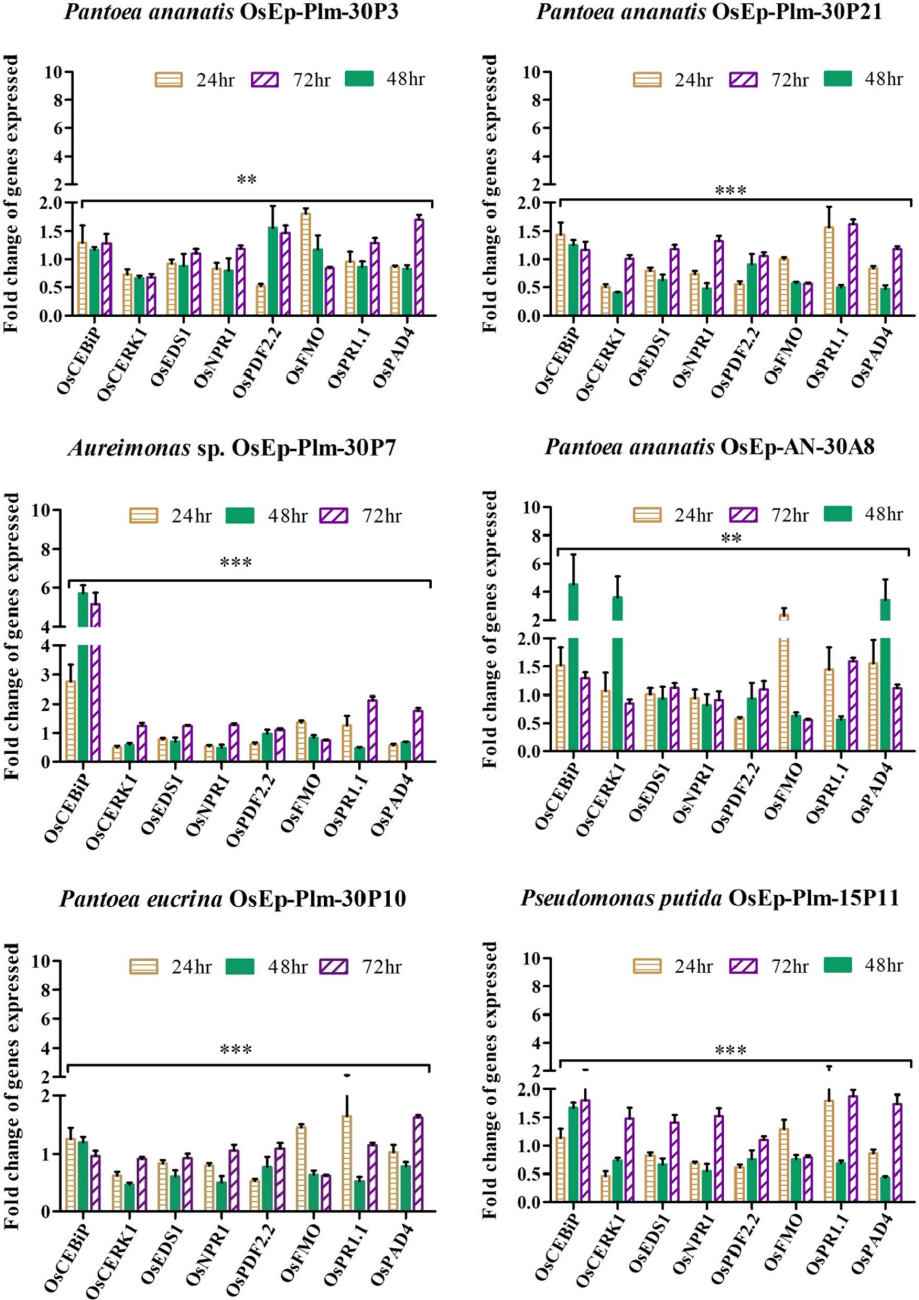
qPCR based transcriptional analysis of defense genes expression in rice seedlings upon phyllobacterization; The fold change values calculated for the defense genes expression were imported into the GraphPad Prism program (https://www.graphpad.com/scientific-software/prism) and two way ANOVA was conducted using Bonferroni Post-hoc test for determining the statistical significance at *P ≤ 0.05, **P = 0.001 and ***P = 0.0001. Note: Refer to Additional file 1: Table S9 for data pertaining to fold changes in gene expression

The phyllosphere bacteria-mediated activation of defense genes was more pronounced during early time points peaking at 48 hpi with a sharp drop at 72 h of bacterial interaction. Defense genes such as *OsCEBiP*, *OsCERK1*, *OsPAD4*, *OsNPR1*, *OsPDF2.2*, *OsFMO1*, and *OsPR1.1 *showed marginal to a high level of expression in phyllobacterized rice seedlings with reference to *OsActin*. All six phyllosphere bacterial species such as *Pantoea ananatis* OsEp-Plm-30P3, *Aureimonas* sp. OsEp-Plm-30P7, *Pantoea eucrina* OsEp-Plm-30P10, *Pantoea ananatis* OsEp-Plm-30P21, *Pseudomonas putida* OsEp-Plm-15P11, and *Pantoea ananatis* OsEp-AN-30A8 induced expression of *OsCEBiP* in rice seedlings. However, significant expression of *OsCEBiP, OsCERK1* and *OsPAD4* were observed in rice seedlings sprayed with *Pantoea* or *Aureimonas.* Strikingly, *Aureimonas* sp. OsEp-Plm-30P7 showed sustained over-expression of *OsCEBiP* in 24, 48, and 72 hpi (Fig. 8; Additional file 2: Fig. S14; Additional file 1: Table S9).

## Discussion

Plant microbiome explorations in the past have revealed highly complex microbial 'assemblages and networks' associated with plant species modulating plant physiological and ecological functions. Metagenomes, the total genomic contents of microbiota and that of the plant, are predicted to possess diverse metabolic capabilities usually not found in plants per se. The plant microbiome plays a versatile ecosystem function through its competitive and cooperative activities leading to nutrient cycling, plant growth, health, and survival [3, 55–59]. Mills et al. [59] proposed a concept of keystone microbial species which is central to the microbial community assemblage and the sustainability of the ecological niche. Microbial communities developing an intimate association with that of plant species during their co-evolution are termed core microbiome which is vertically transmitted across successive plant generations [60]. Nevertheless, microbiome composition and their functions in plant niches are influenced by biotic and abiotic factors as well as macro and microclimatic variables [61]. It is further reported that long-term seasonal patterns related to climatic variations serve a vital role in shaping the phyllosphere microbiome as compared to short-term weather fluctuations during the crop season [62].

The phyllosphere is one of the habitats for diverse microorganisms that are adapted to survive intra-day vagaries of weather. The major drivers of phyllosphere microbiome structure and composition are not adequately understood. Though speculated from the microbiome profiles of diverse genotypes, the core phyllomicrobiome of rice is not elucidated yet. Most of the phyllomicrobiome studies, till now, focused on microbiome profiling using mNGS methods alone. Integrated microbiome analysis by adopting metabarcoding and culturomic methods was performed on two rice genotypes differing in their reaction to blast disease planted in contrasting agroclimatic zones.

While the current blast mitigation strategy by R-genes is threatened by new pathotypes, the fungicide is environmentally unsafe and is no longer accepted in trade [30, 63]. Hence, there is a need for alternative approaches for blast disease management preferably through eco-friendly strategies. We integrated the culturomics with metabarcoding methods not only to validate the mNGS data but also for developing phyllomicrobiome based inoculants for blast management.

Members of phyla Proteobacteria, Firmicutes, Actinobacteria, and Bacteroidetes were found over represented in the phyllosphere of the resistant and susceptible rice genotypes planted in both the zones. Proteobacteria dominance in the phyllosphere of diverse plant species is reported by many workers [64–66]. Recently, in an exhaustive study Roman-Reyna et al. [67] observed a region-specific microbial hub representing diverse families on the rice phyllosphere. The rice genotypes, PRR78 and Pusa1602, planted in contrasting climatic zones showed co-occurrence of *Acinetobacter*, *Arthrobacter*, *Bacillus*, *Curtobacterium*, *Enterobacter*, *Exiguobacterium*, *Kineococcus*, *Methylobacterium*, *Microbacterium*, *Paenibacillus*, *Pantoea*, *Pseudoalteromonas*, *Pseudomonas*, *Rhodococcus*, and *Sphingomonas *that can be considered as core phyllomicrobiome. According to Eyre et al. [69], an ideal core microbiome is the microbial communities shared between genotypes grown in geographical areas that do not share common environmental conditions. Bacterial genera such as *Curtobacterium, Enterobacter, Methylobacterium, Microbacterium*, and *Sphingomonas* are frequently reported as the core microbiome of rice [68, 69]. Kim et al. [70] reported dominance of *Pantoea* (42.5%), *Methylobacterium* (11.8%), *Curtobacterium* (9.3%), *Pseudomonas* (8.7%), and *Sphingomonas* (8.6%) on rice spermosphere who further highlighted that the seed microbiome is highly stable and protected owing to their natural encapsulation in the seed coat that enables them to be inherited, known as vertical transmission. Coupled with the recent evidence from rice seed microbiomes, it is highly probable that the rice seeds played a carrier of the microbiome that enabled its spatiotemporal transmission.

The study further revealed genotype-specific association of *Actinomycetaceae, Aerococcaceae, Burkholderiaceae, Caulobacteraceae, Corynebacteriaceae, Dietziaceae, Sphingobacteriaceae,* and *Staphylococcaceae* in Pusa1602 and *Clostridiaceae, Intrasporangiaceae,* and *Oxalobacteraceae* in PPR78. The impact of R gene introgression on phyllomicrobiome composition and assemblage is reported [67]. From the results, it appears that the impact is highly variable and unpredictable. For instance, the rice line IR24 introgressed with bacterial blight resistance gene *Xa4* showed an increased abundance of Proteobacteria and Firmicutes and a reduced abundance of *Actinobacteria*. However, the rice line R711 + SAox showed a decreased abundance of *Firmicutes* and an increased Proteobacteria abundance. Nonetheless, a significant influence of plant genotype on rhizosphere and endosphere microbiome is also reported [71–73].

A total of 78 diverse bacterial isolates representing 13 genera and 26 species were isolated and characterized from the rice phyllosphere. The intergenic amplicon profiling by BOX PCR -one of the discriminatory molecular tools in bacteriology, indicated diverse bacterial communities [39, 74]. The most frequented bacterial species in the cultivated phyllomicrobiome belonged to *Acinetobacter, Acidovorax, Curtobacterium, Enterobacter, Pantoea, Pseudomonas,* and *Sphingomonas* which were also recorded in the mNGS data.

The four-week-old rice seedlings showed more phyllobacterial diversity and richness as compared to two weeks old seedlings suggestive of the expansion of microbial colonization upon plant ageing. Interestingly, as many as six bacterial isolates from the mountain zone and four from the island zone were found sharing all intergenic amplicons suggestive of their genetic similarity. A genetically identical bacterial isolate is identified as *Pantoea ananatis* from the two agroclimatic zones. Interception of genetically identical *Pantoea ananatis* representing the well-separated locations is indicative of vertical transmission. Recently Charishma [75] reported a high-frequency occurrence of *Pantoea ananatis* on rice spermosphere and phyllosphere of Pusa Basmati-1 and VLD85. Taken together, it is tempting to suggest that the spermosphere bacterial pool seems to have contributed to the phyllomicrobiome during seedling emergence and subsequent plant growth. Our data on seed transmission of phyllomicrobiome is in agreement with the report of Kim et al. [70].

The core bacterial genera *Acinetobacter* (pale brown), *Aeromonas* (dark brown)*, Aureimonas* (yellow), *Curtobacterium* (yellow; red), *Exiguobacterium* (yellow; orange), *Methylobacterium* (pink)*, Microbacterium* (yellow)*, Micrococcus* (yellow; red)*, Pantoea* (yellow)*,* and *Sphingomonas* (yellow) are well-known pigment producer. Dark pigmentation is touted as an adaptive trait of bacteria and other microbes in the phyllosphere [61, 76]. The pigmentation of many *Aeromonas* species is attributed to L-3, 4-dihydroxyphenylalanine (L-DOPA) based melanin [77]. Rice foliar niche is a well-cited habitat for pink pigmented–facultative methylotrophic (PPFM) bacteria and yellow-pigmented *Pantoea;* both are tolerant to harmful ɣ-ray radiation as well as nutritional and moisture stress [76]. Recently, Carvalho and Castillo [78] reported the significant role of sunlight in shaping the microbiome of the phyllosphere. The intimate association of *Pantoea ananatis* with the phyllosphere of many plants including rice as previously reported [79, 80]. *Microbacterium testaceum* is reported to degrade *N*-acyl-homoserine lactone on a potato leaf and is considered an aggressive plant colonizer involved in natural biocontrol against plant pathogens [81]. *Microbacterium* species are reported in the rice phyllosphere and spermosphere [68, 82, 83]. Phyllosphere acquires microbiome from insect pollinators and passive visitors. Interception of *Asaia* -a mosquito-associated bacteria on phyllosphere samples from Andaman Island that is endemic to malaria is a pointer [84].

Techniques such as fluorescent in situ hybridization (FISH) and SEM are among the methods to visualize native microbial cells as well as to analyze the spatial distribution of cells in the phyllosphere [85, 86]. Our SEM analysis revealed the presence of bacterial cell aggregates of 5–8 cells, and unevenly distributed solitary bacterial cells on the rice phyllosphere. The formation of aggregates by bacterial communities is one of the adaptive mechanisms in the phyllosphere [10, 87]. The cultured bacterial isolates showed antifungal activity on *M. oryzae.* Whereas *Acinetobacter, Pantoea,* and *Pseudomonas* inhibited *M. oryzae* by secreted and volatile metabolites, the *Aureimonas, Erwinia,* and *Exiguobacterium* showed secreted metabolite mediated antagonism. The biocontrol potential of *Acinetobacter baumannii* [88], *Pantoea ananatis* [89], *Pantoea agglomerans* [90], *Pseudomonas oryzihabitans* [91–93], *Pseudomonas putida* [42, 94] is reported*.* Among the foliar-adapted bacterial species, *Pantoea vagans* C9-1isolated from apple is registered as BlightBan C9-1 by Nufarms America Inc., Burr Ridge, IL, the USA for biocontrol of fire blight. Prophylactic phyllobacterization using *Pantoea, Aureimonas, Pseudomonas,* and *Acinetobacter* showed a significant reduction in rice blast. Rice blast suppression by bacterial species belonging to *Bacillus*, *Streptomyces*, *Pseudomonas*, *Pantoea*, *Paenibacillus*, *Burkholderia, Enterobacter*, and *Paraburkholderia* is reported [95–97]. Phyllobacterization conferred immunocompetence in rice leaf as evident from the over-expression of defense genes such as *OsCEBiP*, *OsCERK, OsPR1.1*, *OsNPR1, OsPDF2.2*, *OsFMO,* and *OsPAD4;* among them, significant induction was noted for *OsCEBiP, OsCERK1*, and *OsPAD4* when phyllobacterized with *Pantoea* or *Aureimonas. OsCEBiP* and *OsCERK1* are known to interact with chitin to activate MAMP Triggered Immune (MTI) responses in plants [46]. *OsCERK1* is a receptor-like kinase (RLK) believed to perceive fungal chitin and bacterial peptidoglycan [47]. *OsPAD4* and *OsEDS1* play a key role in jasmonic acid-mediated induced systemic resistance against blast by the accumulation of phytoalexin mamilactone-A [48, 49, 98]. Marginal induction of *OsNPR1*, *OsFMO*, *OsPDF2.2,* and *OsPR1.1* was observed in bacterized seedlings. *OsNPR1* is the central regulator of salicylic acid (SA) mediated defense signaling [50]. Similarly, *OsFMO1* is also an essential component for induced systemic acquired resistance [52, 53]. *OsPDF2.2* is a plant defensin responsible for the inhibition of fungal growth [51]. *OsPR1.1* is an acidic pathogenesis-related protein, and a marker for salicylic acid-mediated SAR [54].

Black pepper endophyte, *Pseudomonas putida* BP25 is recently reported to induce defense against rice blast [94]. Similarly, SA-mediated defense and growth promotion was found induced in arabidopsis by *P. putida* BP25 [99] and *Bacillus megaterium* BP17 [100]. Species belonging to *Microbacterium* and *Stenotrophomonas* have also been recently reported to elicit defense against rice blast disease [101]. Patel et al. [102] recently reported the antifungal and defense elicitation activity of pyrazine against the rice blast disease.

## Conclusion

The agroclimatic zone and the associated environmental factors appear to drive phyllomicrobiome structure and composition in the rice genotypes. We observed a converging phyllomicrobiome assemblage on the phyllosphere when the genotypes shared the same agroclimatic zone. Conversely, divergent phyllomicrobiome assemblage was observed in the rice phyllosphere when planted in contrasting climate zone. Our integrated microbiome interrogation by mNGS and culturomics approaches revealed *Acinetobacter, Aureimonas, Curtobacterium, Enterobacter, Exiguobacterium, Microbacterium, Pantoea, Pseudomonas,* and *Sphingomonas* as core phyllomicrobiome. Genetically identical *Pantoea ananatis* intercepted in the contrasting agroclimatic zone is suggestive of vertical seed-assisted transmission. The phyllobacterization by core-microbiome showed potential for blast suppression by direct antibiosis and defense activation. The identification of phyllosphere-adapted functional core bacterial communities and their co-occurrence dynamics presents an opportunity to devise novel strategies for blast management through phyllomicrobiome reengineering in the future.

## Supplementary Information

**Additional file 1.** **Table S1**. Rice defense genes used for the qPCR analysis and their function. **Table S2**. List of the PCR primers used in the gene expression studies.  **Table S3**. Metagenome read statistics of phyllomicrobiome of rice genotypes grown in two contrasting climatic zone. **Table S4**. Network analysis of rice phyllosphere microbiome using SparCC correlation coefficients. **Table S5**. Population size of epiphytic bacteria (Log CFU g^_1^) on phyllosphere of 15 and 30 days aged rice genotypes grown in Mountain zone. **Table S6**. Population size of epiphytic bacteria (Log CFU g^−1^) on phyllosphere of rice genotypes grown in Island zone. **Table S7**. Identification of bacterial isolates by 16S rRNA gene sequencing. **Table S8**. Analysis of nature of BVC mediated mycelial inhibition of *Magnaporthe oryzae.* **Table S9**. qPCR based transcriptional analysis of defense genes expression in rice seedlings upon phyllobacterization. i. *OsCEBiP* was found induced in all-time points by bacterization; significant induction by *Aureimonas* sp.OsEp-Plm-30P7 for all three-time points and *Pseudomonas putida* OsEp-Plm-15P11 or *Pantoea ananatis* OsEp-AN-30A848 hour post bacterization. ii. *OsPR1.1* was also found induced 72 h post bacterization with significant induction by *Aureimonas* sp.OsEp-Plm-30P7. iii. *OsNPR1* and *OsPDF2.2* showed induction at 72 h post-inoculation for all the bacterial treatments. iv. Other genes induced were *OsFMO* in *Pantoea ananatis* OsEp-AN-30A8, *OsCERK1,* and*OsPAD4* in *Pantoea ananatis* OsEp-AN-30A8.

**Additional file 2**. **Fig. S1**. Identification of biomarkers based on the linear discriminant analysis (LDA) and effect size (LEfSe) pipeline; (A) Two genotypes (PRR78 and Pusa1602); (B) Two locations (Palampur and Port Blair). **Fig. S2**. Network analysis of rice phyllosphere microbiome using SparCC correlation coefficients (Normal group). **Fig. S3**. Extended error bar plot at various taxonomic hierarchy levels for phyllomicrobiome of rice genotypes, PRR78 and Pusa1602.  **Fig. 4**. Extended error bar plot at various taxonomic hierarchy levels for phyllomicrobiome of rice grown in Palampur, Himachal Pradesh and Port Blair, Andaman Island. **Fig. 5**. Relative abundance of phyllosphere bacterial communities on rice genotypes grown in two agroclimatic zones of India. **Fig. S6**. Relative abundance of phyllosphere bacterial communities at genus level on two rice genotypes representing contrasting agroclimatic zones of India. **Fig. 7**. BOX PCR fingerprinting of cultured bacterial isolates of rice phyllosphere; M: DNA size marker; Lanes: Isolates of bacteria isolated from the phyllosphere of rice leaf. **Fig. 8**. Amplification of 16S rRNA of bacterial isolates of rice phyllosphere. **Fig. 9a**. Colonies of cultured *Acidovorax* species from rice phyllomicrobiome. **Fig. S9b**. Colonies of cultured *Acinetobacter* species from rice phyllomicrobiome. **Fig. 9c**. Colonies of cultured *Agrobacterium* species from rice phyllomicrobiome. **Fig. S9d**. Colonies of cultured *Aureimonas* species from rice phyllomicrobiome. **Fig. S9e**. Colonies of cultured *Curtobacterium* species from rice phyllomicrobiome. **Fig. 9f**. Colonies of cultured *Enterobacter* species from rice phyllomicrobiome. **Fig. S9g**. Colonies of cultured *Erwinia* species from rice phyllomicrobiome. **Fig. 9h**. Colonies of cultured *Exiguobacterium* species from rice phyllomicrobiome. **Fig. S9i**. Colonies of cultured *Microbacterium* species from rice phyllomicrobiome. **Fig. S9j**. Colonies of cultured *Micrococcus* species from rice phyllomicrobiome. **Fig. S9k**. Colonies of cultured *Pantoea* species from rice phyllomicrobiome. **Fig. S9l**. Colonies of cultured *Pseudomonas* species from rice phyllomicrobiome. **Fig. 9m**. Colonies of cultured *Sphingomonas* species from rice phyllomicrobiome. **Fig. S10**. Secreted metabolite mediated in vitro antifungal activity of rice phyllosphere bacterial isolates on *Magnaporthe oryzae.* **Fig. S11**. Volatile mediated in vitro antifungal activity of rice phyllosphere bacterial isolates on *Magnaporthe oryzae.* **Fig. S12**. Analysis of nature of BVC mediated growth inhibition of *Magnaporthe oryzae.* **Fig. S13**. Effect of phyllobacterization on rice blast disease incited by *Magnaporthe oryzae.*  **Fig. S14**. qPCR based transcriptional analysis of defense genes expression in rice seedlings upon phyllobacterization.

## Data Availability

Data sets were submitted to NCBI GenBank with BioProject ID PRJNA681302. The data sets were also uploaded in MG-RAST server under project ID mgp94842 with following sample name and deposition numbers; PRR78_Plm1 (mgm4895994.3); PRR78_Plm2 (mgm4895995.3); Pusa1602_Plm1 (mgm4895999.3); Pusa1602_Plm2 (mgm4896000.3); PRR78_ANI1 (mgm4895998.3); PRR78_ANI2 (mgm4896001.3); Pusa1602_ANI1 (mgm4895997.3); Pusa1602_ANI2 (mgm4895996.3). All bacterial cultures and fungal isolate are available in the Division of Plant Pathology, ICAR-IARI, New Delhi.

## Abbreviations

ANI: Andaman and Nicobar Islands
ANOSIM: ANalysis of SIMilarities
ANOVA: Analysis of variance
BVC: Bacterial volatile compounds
CD: Critical Difference
CFU: Colony Forming Units
CTAB: Cetyl Trimethyl Ammonium Bromide
CV: Coefficient of variation
Km: Kilometer
LDA-LEfSe: Linear discriminant analysis (LDA) effect size (LEfSe) method
MG-RAST: Metagenomic Rapid Annotations using Subsystems Technology
mNGS: Metagenomic Next-Generation Sequencing
NA: Nutrient agar
NextGen-Crop-care: Next-Generation technology for Crop health management
OTU: Operational Taxonomic Units
PBS: Phosphate Buffered Saline
PBS-T: Tween 20 amended Phosphate Buffered Saline
PCoA: Principal Coordinate analysis
PEAR: Paired-End reAd mergeR
Phyllobacterization: A term coined for spraying of bacterial cell suspension on phyllosphere
Phyllomicrobiome: Microbiome adapted on above-ground plant foliar parts including leaf
Phytosphere: Plant associated epi and endophytic niches
Plm: Palampur, India
qPCR: Quantitative Real-Time Polymerase Chain Reaction
RDS: Reduction in disease severity
SC: Secreted compounds of bacteria
SEd: Standard Error of the difference between two means
SEM: Scanning electron microscopy
SEm: Standard Error of the mean
STAMP: Statistical Analysis of Metagenomic Profile
TSS: Total Sum Scaling
