# Supplementary material for: Deciphering core phyllomicrobiome assemblage on rice genotypes grown in contrasting agroclimatic zones: implications for phyllomicrobiome engineering against blast disease
Source: Environ Microbiome. 2022 May 26;17:28. doi: 10.1186/s40793-022-00421-5 (PMC9134649; doi:10.1186/s40793-022-00421-5)
Supplement: Supplementary file 1 — Additional file 1. Table S1. Rice defense genes used for the qPCR analysis and their function. Table S2. List of the PCR primers used in the gene expression studies. Table S3. Metagenome read statistics of phyllomicrobiome of rice genotypes grown in two contrasting climatic zone. Table S4. Network analysis of rice phyllosphere microbiome using SparCC correlation coefficients. Table S5. Population size of epiphytic bacteria (Log CFU g_1) on phyllosphere of 15 and 30 days aged rice genotypes grown in Mountain zone. Table S6. Population size of epiphytic bacteria (Log CFU g−1) on phyllosphere of rice genotypes grown in Island zone. Table S7. Identification of bacterial isolates by 16S rRNA gene sequencing. Table S8. Analysis of nature of BVC mediated mycelial inhibition of Magnaporthe oryzae. Table S9. qPCR based transcriptional analysis of defense genes expression in rice seedlings upon phyllobacterization. i. OsCEBiP was found induced in all-time points by bacterization; significant induction by Aureimonas sp.OsEp-Plm-30P7 for all three-time points and Pseudomonas putida OsEp-Plm-15P11 or Pantoea ananatis OsEp-AN-30A848 hour post bacterization. ii. OsPR1.1 was also found induced 72 h post bacterization with significant induction by Aureimonas sp.OsEp-Plm-30P7. iii. OsNPR1 and OsPDF2.2 showed induction at 72 h post-inoculation for all the bacterial treatments. iv. Other genes induced were OsFMO in Pantoea ananatis OsEp-AN-30A8, OsCERK1, andOsPAD4 in Pantoea ananatis OsEp-AN-30A8. [file 40793_2022_421_MOESM1_ESM.docx]

**Supplementary Table 1. Rice defense genes used for the qPCR analysis and their function**

| **Gene** | **Characteristics and function** | **Reference** |
| --- | --- | --- |
| *OsCEBiP*  (Chitin Elicitor Binding Protein) | It is a Pattern Recognition Receptor (PRR) that detects the pathogen PAMP molecule chitin and activates plant defense system. It is a plasma membrane protein that forms a receptor complex essential for fungal chitin-driven immune responses in rice. | Akamatsu et al. 2013 |
| *OsCERK1*  (Chitin Elicitor Receptor Kinase) | It is a rice receptor-like kinase (RLK) that mediates the signal of a fungal cell wall component chitin. It indispensable for chitin perception and participates in innate immunity. It can mediate the signaling pathways of both fungal and bacterial PAMP molecules. | Kouzai et al. 2014 |
| *OsPAD4* (Phytoalexin deficient 4) | Phytoalexin deficient 4 (PAD4) induces JA-dependent induced systemic resistance. It also plays an important role in accumulation of JA and a terpenoid-type phytoalexin momilactone A (MOA). | Ke et al. 2014 |
| *OsEDS1*  (Enhanced disease susceptibility 1) | Enhanced disease susceptibility 1 (EDS1) induces JA-dependent induced systemic resistance. | Ke et al. 2019 |
| *OsNPR1* (Non Repressor of Pathogenesis related Protein) | A central regulator of salicylic-acid (SA)-mediated defense signaling  Reallocation of energy and resources during defense responses | Sugano et al. 2010 |
| *OsPDF2.2* (Plant Defensin-like protein 2) | Plant defensin inhibit the growth of a broad range of fungi | Thomma et al. 2002 |
| *OsFMO1* (Favin-dependent Monooxygenase 1) | An essential component for induced systemic acquired resistance (SAR) | Mishina et al 2006; Koch et al 2006 |
| *OsPR1.1* (Pathogenesis related protein 1) | Acidic pathogenesis related protein 1, among the most abundant antimicrobial protein. It is also a marker for salicylic acid mediated SAR | Breen et al 2017 |

**Supplementary Table 2. List of the PCR primers used in the gene expression studies**

| **SN** | **Gene** |  | **Primer sequence(5→3)** | **Number of bases** | **Product size** |
| --- | --- | --- | --- | --- | --- |
| 1 | *Os*PAD4 | Forward | TGCCGACTACCACCGAAAC | 19 | 61 |
|  |  | Reverse | CCGGCCATGGGTGATGTA | 18 |  |
| 2 | *Os*EDS1 | Forward | TTGAATTTTGTCGTGCCAGTAGA | 23 | 63 |
|  |  | Reverse | GGCAGATGCAAGCGGAGTAA | 20 |  |
| 3 | *Os*CEBiP | Forward | GTGCGGAGAAGTCTGGAAAG | 20 | 131 |
|  |  | Reverse | TCCTGATTTCGCTTGCTTTT | 20 |  |
| 4 | *Os*CERK1 | Forward | AAGAACTACCGGGCAAAGGT | 20 | 244 |
|  |  | Reverse | GCCCCTTTGAATCACTTGAA | 20 |  |
| 5 | *Os*PDF2.2 | Forward | CCACAGGTTCAAGGGCATGT | 20 | 63 |
|  |  | Reverse | CTCTCCGTCCTGCACACGTT | 20 |  |
| 6 | *Os*NPR1 | Forward | AAACAAAGGAGCAGCTGTATCACA | 24 | 66 |
|  |  | Reverse | CTCCGGCAGATACTCATTGCA | 21 |  |
| 7 | *Os*FMO1 | Forward | CAGTGGAGTGCCCAACATACC | 21 | 65 |
|  |  | Reverse | CCTGGCCATCAAATGCTTCT | 20 |  |
| 8 | *Os* PR1.1 | Forward | GGAGGCATCCAAGCTAGCAA | 20 | 80 |
|  |  | Reverse | GGGCATCGGAGCAGTGAA | 18 |  |
| 9 | *Os*Actin | Forward | CAGCCACACTGTCCCCATCTA | 21 | 67 |
|  |  | Reverse | AGCAAGGTCGAGACGAAGGA | 20 |  |

Primers designed using online platform Primer3Plus <http://www.bioinformatics.nl/cgi-bin/primer3plus/primer3plus.cgi>

**Supplementary Table 3. Metagenome read statistics of phyllomicrobiome of rice genotypes grown in two contrasting climatic zone**

| **Location** | **Sample Name** | ***MG-RAST accession No**  **(Project ID mgp94842)** | ****NCBI-Genbank accession No. (Bio Project ID PRJNA681302)** | **Number of base pairs (bp)** | **Total number of sequences** | **Mean Sequence Length (bp)** |
| --- | --- | --- | --- | --- | --- | --- |
| **Palampur (HP), 2016** | **PRR78_Plm1** | mgm4895994.3 | SRR13171790 | 20,127,006 | 43,818 | 459 ± 18 |
|  | **PRR78_Plm2** | mgm4895995.3 | SRR13355773 | 12,296,139 | 26,794 | 459 ± 17 |
|  | **Pusa1602_Plm1** | mgm4895999.3 | SRR13171789 | 17,306,479 | 38,369 | 451 ± 22 |
|  | **Pusa1602_Plm2** | mgm4896000.3 | SRR13355772 | 10,617,288 | 23,552 | 451 ± 21 |
| **Port Blair**  **Andaman Island, 2017** | **PRR78_ANI1** | mgm4895998.3 | SRR13171788 | 43,236,085 | 96,317 | 449 ± 23 |
|  | **PRR78_ANI2** | mgm4896001.3 | SRR13355771 | 8,316,792 | 18,933 | 439 ± 46 |
|  | **Pusa1602_ANI1** | mgm4895997.3 | SRR13171787 | 58,289,850 | 129,470 | 450 ± 22 |
|  | **Pusa1602_ANI2** | mgm4895996.3 | SRR13355770 | 11,173,095 | 25,340 | 441 ± 45 |

*<https://www.mg-rast.org/>

** <https://www.ncbi.nlm.nih.gov/>

**Supplementary Table 4. Network analysis of rice phyllospheric microbiome using SparCC correlation coefficients**

| **Genus** | ***Positively Correlated Genus*** | **Correlation** | ***Negatively Correlated Genus*** | **Correlation** |
| --- | --- | --- | --- | --- |
| **Acidovorax** | ***Macrococcus*** | 0.743 | ***Asaia*** | -0.739 |
|  | ***Sphingopyxis*** | 0.829 | ***Enterococcus*** | -0.774 |
|  | ***unclassified-(derived-from-Alphaproteobacteria)*** | 0.944 | ***Leptolyngbya*** | -0.652 |
|  |  |  | ***unclassified-(derived-from-Bacteria)*** | -1.000 |
|  |  |  | ***Xenococcus*** | -0.729 |
|  |  |  |  |  |
| **Acinetobacter** | ***Arthrobacter*** | 0.743 | ***Bacillus*** | -0.790 |
|  | ***Exiguobacterium*** | 0.781 | ***Citrobacter*** | -0.743 |
|  | ***Flavobacterium*** | 0.853 | ***Tetrasphaera*** | -0.798 |
|  | ***Pseudomonas*** | 0.805 |  |  |
|  | ***Riemerella*** | 0.811 |  |  |
|  |  |  |  |  |
| **Agrobacterium** | ***Leucobacter*** | 0.885 | ***Chryseobacterium*** | -0.696 |
|  |  |  |  |  |
| **Agromyces** | ***Clavibacter*** | 0.851 | ***Paenibacillus*** | -0.845 |
|  | ***Kineococcus*** | 1.000 | ***Sphingobacterium*** | -0.755 |
|  | ***Lactobacillus*** | 0.710 |  |  |
|  | ***Leifsonia*** | 0.903 |  |  |
|  | ***Okibacterium*** | 0.847 |  |  |
|  |  |  |  |  |
| **Arthrobacter** | ***Acinetobacter*** | 0.743 | ***Bacillus*** | -0.975 |
|  | ***Erwinia*** | 0.826 | ***Citrobacter*** | -0.714 |
|  | ***Pseudoalteromonas*** | 0.761 | ***Clostridium*** | -0.802 |
|  | ***Pseudomonas*** | 0.736 | ***Lactobacillus*** | -0.859 |
|  | ***Shewanella*** | 0.952 | ***unclassified-(derived-from-Oscillatoriales)*** | -0.753 |
|  | ***Sphingopyxis*** | 0.801 |  |  |
|  |  |  |  |  |
| **Arthrospira** | ***Clostridium*** | 0.644 | ***Shewanella*** | -0.647 |
|  | ***Kurthia*** | 0.819 |  |  |
|  | ***Lyngbya*** | 0.777 |  |  |
|  | ***Mycetocola*** | 0.770 |  |  |
|  |  |  |  |  |
| **Asaia** | ***Enterococcus*** | 0.713 | ***Acidovorax*** | -0.739 |
|  | ***Leptolyngbya*** | 0.732 | ***unclassified-(derived-from-Alphaproteobacteria)*** | -0.912 |
|  | ***unclassified-(derived-from-Bacteria)*** | 1.000 |  |  |
|  | ***Xanthomonas*** | 0.798 |  |  |
|  | ***Xenococcus*** | 0.901 |  |  |
|  |  |  |  |  |
| **Azorhizobium** |  |  | ***Rhizobium*** | -0.670 |
|  |  |  | ***Spirosoma*** | -0.691 |
|  |  |  |  |  |
| **Bacillus** | ***Bifidobacterium*** | 0.852 | ***Acinetobacter*** | -0.790 |
|  | ***Citrobacter*** | 0.747 | ***Arthrobacter*** | -0.975 |
|  | ***Clostridium*** | 0.952 | ***Erwinia*** | -0.943 |
|  | ***Lactobacillus*** | 0.864 | ***Flavobacterium*** | -0.749 |
|  | ***unclassified-(derived-from-Oscillatoriales)*** | 0.814 | ***Pseudoalteromonas*** | -0.756 |
|  |  |  | ***Shewanella*** | -0.889 |
|  |  |  | ***unclassified-(derived-from-unclassified-sequences)*** | -0.896 |
|  |  |  |  |  |
| **Bifidobacterium** | ***Bacillus*** | 0.852 | ***Erwinia*** | -0.812 |
|  | ***Clostridium*** | 0.835 | ***Pseudoalteromonas*** | -0.843 |
|  | ***Lactobacillus*** | 0.965 | ***Shewanella*** | -0.931 |
|  | ***unclassified-(derived-from-Oscillatoriales)*** | 0.948 | ***Sphingopyxis*** | -0.825 |
|  | ***Xenococcus*** | 0.672 |  |  |
|  |  |  |  |  |
| **Burkholderia** | ***Micrococcus*** | 0.878 |  |  |
|  |  |  |  |  |
| **Candidatus-Aquiluna** | ***Sphingomonas*** | 0.753 | ***Sphingobacterium*** | -0.696 |
|  | ***unclassified-(derived-from-Bacteria)*** | 1.000 | ***unclassified-(derived-from-Alphaproteobacteria)*** | -0.825 |
|  | ***unclassified-(derived-from-Oscillatoriales)*** | 0.702 |  |  |
|  | ***Xenococcus*** | 0.635 |  |  |
|  |  |  |  |  |
| **Chryseobacterium** | ***Pedobacter*** | 0.880 | ***Agrobacterium*** | -0.696 |
|  | ***Rhodococcus*** | 0.805 | ***Clavibacter*** | -0.842 |
|  | ***Spirosoma*** | 0.866 | ***Cronobacter*** | -0.841 |
|  |  |  | ***Leifsonia*** | -0.720 |
|  |  |  | ***Leucobacter*** | -0.863 |
|  |  |  | ***Okibacterium*** | -0.776 |
|  |  |  |  |  |
|  |  |  | ***unclassified-(derived-from-Enterobacteriaceae)*** | -0.802 |
| **Citrobacter** | ***Bacillus*** | 0.747 | ***Acinetobacter*** | -0.743 |
|  | ***Cronobacter*** | 0.751 | ***Arthrobacter*** | -0.714 |
|  | ***Staphylococcus*** | 0.694 | ***Exiguobacterium*** | -0.930 |
|  | ***Tetrasphaera*** | 0.701 | ***Flavobacterium*** | -0.876 |
|  |  |  | ***Pseudomonas*** | -0.820 |
|  |  |  |  |  |
| **Clavibacter** | ***Agromyces*** | 0.851 | ***Chryseobacterium*** | -0.842 |
|  | ***Cronobacter*** | 0.778 | ***Deinococcus*** | -0.873 |
|  | ***Kineococcus*** | 1.000 | ***Paenibacillus*** | -0.872 |
|  | ***Leifsonia*** | 0.943 | ***Sphingobacterium*** | -0.795 |
|  | ***Leucobacter*** | 0.944 | ***Spirosoma*** | -0.816 |
|  | ***Okibacterium*** | 0.790 |  |  |
|  |  |  |  |  |
| **Clostridium** | ***Arthrospira*** | 0.644 | ***Arthrobacter*** | -0.802 |
|  | ***Bacillus*** | 0.952 | ***Erwinia*** | -0.848 |
|  | ***Bifidobacterium*** | 0.835 | ***Pseudoalteromonas*** | -0.772 |
|  | ***Lactobacillus*** | 0.855 | ***Shewanella*** | -0.819 |
|  | ***Mycetocola*** | 0.754 |  |  |
|  | ***unclassified-(derived-from-Oscillatoriales)*** | 0.810 |  |  |
|  |  |  |  |  |
| **Cronobacter** | ***Citrobacter*** | 0.751 | ***Chryseobacterium*** | -0.841 |
|  | ***Clavibacter*** | 0.778 | ***Enterococcus*** | -0.754 |
|  | ***Escherichia*** | 0.735 | ***Flavobacterium*** | -0.623 |
|  | ***Leucobacter*** | 0.822 | ***Paenibacillus*** | -0.781 |
|  | ***Serratia*** | 0.775 | ***Pseudomonas*** | -0.767 |
|  |  |  |  |  |
| **Curtobacterium** | ***Rhizobium*** | 0.775 | ***Pseudoalteromonas*** | -0.714 |
|  | ***unclassified-(derived-from-Oscillatoriales)*** | 0.844 | ***Sphingopyxis*** | -0.791 |
|  |  |  |  |  |
| **Deinococcus** | ***Frankia*** | 0.772 | ***Clavibacter*** | -0.873 |
|  | ***Hymenobacter*** | 0.892 | ***Kineococcus*** | -1.000 |
|  | ***Rhodococcus*** | 0.846 | ***Leucobacter*** | -0.819 |
|  | ***Sphingobacterium*** | 0.716 | ***Methylocella*** | -0.802 |
|  | ***Spirosoma*** | 0.840 | ***Okibacterium*** | -0.799 |
|  |  |  |  |  |
| **Enterococcus** | ***Asaia*** | 0.713 | ***Acidovorax*** | -0.774 |
|  | ***Leptolyngbya*** | *0.771* | ***Cronobacter*** | *-0.754* |
|  | ***Xenococcus*** | 0.782 | ***Serratia*** | -0.741 |
|  |  |  |  |  |
| **Erwinia** | ***Arthrobacter*** | 0.826 | ***Bacillus*** | -0.943 |
|  | ***Pseudoalteromonas*** | 0.801 | ***Bifidobacterium*** | -0.812 |
|  | ***Shewanella*** | 0.858 | ***Clostridium*** | -0.848 |
|  | ***Sphingopyxis*** | 0.708 | ***Lactobacillus*** | -0.774 |
|  |  |  | ***Microcoleus*** | -0.664 |
|  |  |  | ***unclassified-(derived-from-Oscillatoriales)*** | -0.796 |
|  |  |  |  |  |
| **Escherichia** | ***Cronobacter*** | 0.735 |  |  |
|  | ***Serratia*** | 0.851 |  |  |
|  |  |  |  |  |
| **Exiguobacterium** | ***Acinetobacter*** | 0.781 | ***Citrobacter*** | -0.930 |
|  | ***Flavobacterium*** | 0.924 | ***Staphylococcus*** | -0.858 |
|  |  |  | ***Tetrasphaera*** | -0.829 |
|  |  |  |  |  |
| **Flavobacterium** | ***Acinetobacter*** | 0.853 | ***Bacillus*** | -0.749 |
|  | ***Exiguobacterium*** | 0.924 | ***Citrobacter*** | -0.876 |
|  | ***Pedobacter*** | 0.688 | ***Cronobacter*** | -0.623 |
|  | ***Pseudomonas*** | 0.710 | ***Staphylococcus*** | -0.741 |
|  |  |  | ***Tetrasphaera*** | -0.778 |
|  |  |  | ***unclassified-(derived-from-Bacteria)*** | -1.000 |
|  |  |  |  |  |
| **Frankia** | ***Deinococcus*** | 0.772 | ***Methylocella*** | -0.698 |
|  | ***Hymenobacter*** | 0.778 |  |  |
|  | ***Janibacter*** | 0.966 |  |  |
|  | ***Methylobacterium*** | 0.793 |  |  |
|  |  |  |  |  |
| **Hymenobacter** | ***Deinococcus*** | 0.892 | ***Okibacterium*** | -0.787 |
|  | ***Frankia*** | 0.778 |  |  |
|  | ***Kineococcus*** | 0.860 |  |  |
|  | ***Methylobacterium*** | 0.859 |  |  |
|  | ***Rhodococcus*** | 0.799 |  |  |
|  | ***Spirosoma*** | 0.802 |  |  |
|  |  |  |  |  |
| **Janibacter** | ***Frankia*** | 0.966 | ***Methylocella*** | -0.737 |
|  | ***Methylobacterium*** | 0.829 |  |  |
|  |  |  |  |  |
| **Kineococcus** | ***Agromyces*** | 1.000 | ***Deinococcus*** | -1.000 |
|  | ***Clavibacter*** | 1.000 | ***Rhodococcus*** | -1.000 |
|  | ***Hymenobacter*** | 0.860 | ***Sphingobacterium*** | -1.000 |
|  | ***Leifsonia*** | 1.000 | ***Spirosoma*** | -1.000 |
|  | ***Leucobacter*** | 1.000 |  |  |
|  | ***Okibacterium*** | 1.000 |  |  |
|  | ***Pedobacter*** | 0.882 |  |  |
|  |  |  |  |  |
| **Klebsiella** | ***Pseudomonas*** | 0.699 |  |  |
|  | ***Sphingomonas*** | 0.805 |  |  |
|  |  |  |  |  |
| **Kurthia** | ***Arthrospira*** | 0.819 | ***Renibacterium*** | -0.686 |
|  | ***Lactobacillus*** | 0.751 |  |  |
|  | ***Leifsonia*** | 0.819 |  |  |
|  | ***Mycetocola*** | 0.728 |  |  |
|  |  |  |  |  |
| **Lactobacillus** | ***Agromyces*** | 0.710 | ***Arthrobacter*** | -0.859 |
|  | ***Bacillus*** | 0.864 | ***Erwinia*** | -0.774 |
|  | ***Bifidobacterium*** | 0.965 | ***Macrococcus*** | -0.744 |
|  | ***Clostridium*** | 0.855 | ***Pseudoalteromonas*** | -0.848 |
|  | ***Kurthia*** | 0.751 | ***Shewanella*** | -0.927 |
|  | ***Leifsonia*** | 0.832 | ***Sphingopyxis*** | -0.789 |
|  | ***unclassified-(derived-from-Oscillatoriales)*** | 0.889 |  |  |
|  |  |  |  |  |
| **Leifsonia** | ***Agromyces*** | 0.903 | ***Chryseobacterium*** | -0.720 |
|  | ***Clavibacter*** | 0.943 | ***Paenibacillus*** | -0.873 |
|  | ***Kineococcus*** | 1.000 | ***Sphingobacterium*** | -0.822 |
|  | ***Kurthia*** | 0.819 |  |  |
|  | ***Lactobacillus*** | 0.832 |  |  |
|  | ***Leucobacter*** | 0.799 |  |  |
|  | ***unclassified-(derived-from-Bacteria)*** | 1.000 |  |  |
|  |  |  |  |  |
| **Leptolyngbya** | ***Asaia*** | 0.732 | ***Acidovorax*** | -0.652 |
|  | ***Enterococcus*** | 0.771 | ***Chryseobacterium*** | -0.863 |
|  | ***Rhizobium*** | 0.683 | ***Deinococcus*** | -0.819 |
|  | ***unclassified-(derived-from-Oscillatoriales)*** | 0.821 | ***Paenibacillus*** | -0.865 |
|  | ***Xenococcus*** | 0.923 | ***Sphingobacterium*** | -0.722 |
|  | ***Agrobacterium*** | 0.885 | ***Spirosoma*** | -0.754 |
|  | ***Clavibacter*** | 0.944 |  |  |
|  | ***Cronobacter*** | 0.822 |  |  |
|  | ***Kineococcus*** | 1.000 |  |  |
|  | ***Leifsonia*** | 0.799 |  |  |
|  | ***Okibacterium*** | 0.736 |  |  |
|  | ***Serratia*** | 0.717 |  |  |
|  |  |  |  |  |
| **Lyngbya** | ***Arthrospira*** | 0.777 |  |  |
|  | ***Rothia*** | 0.813 |  |  |
|  | ***unclassified-(derived-from-Alphaproteobacteria)*** | 0.783 |  |  |
|  |  |  |  |  |
| **Macrococcus** | ***Acidovorax*** | 0.743 | ***Lactobacillus*** | -0.744 |
|  | ***Nocardioides*** | 0.798 | ***unclassified-(derived-from-Oscillatoriales)*** | -0.717 |
|  | ***Pseudoalteromonas*** | 0.667 |  |  |
|  | ***Shewanella*** | 0.731 |  |  |
|  | ***Sphingobacterium*** | 0.651 |  |  |
|  | ***Sphingopyxis*** | 0.748 |  |  |
|  | ***Staphylococcus*** | 0.753 |  |  |
|  |  |  |  |  |
| **Massilia** |  |  | ***unclassified-(derived-from-Enterobacteriaceae)*** | -0.770 |
|  |  |  |  |  |
| **Methylobacterium** | ***Frankia*** | 0.793 | ***Methylocella*** | -0.745 |
|  | ***Hymenobacter*** | 0.859 | ***unclassified-(derived-from-unclassified-sequences)*** | -0.884 |
|  | ***Janibacter*** | 0.829 |  |  |
|  | ***Nocardioides*** | 0.827 |  |  |
|  | ***Staphylococcus*** | 0.784 |  |  |
|  |  |  |  |  |
| **Methylocella** |  |  | ***Deinococcus*** | -0.802 |
|  |  |  | ***Frankia*** | -0.698 |
|  |  |  | ***Janibacter*** | -0.737 |
|  |  |  | ***Methylobacterium*** | -0.745 |
|  |  |  | ***Sphingobacterium*** | -0.782 |
|  |  |  | ***unclassified-(derived-from-Bacteria)*** | -0.763 |
|  |  |  |  |  |
| **Micrococcus** | ***Burkholderia*** | 0.878 | ***Xenococcus*** | -0.646 |
|  |  |  |  |  |
| **Microcoleus** |  |  | ***Erwinia*** | -0.664 |
|  |  |  | ***unclassified-(derived-from-unclassified-sequences)*** | -0.950 |
|  |  |  |  |  |
| **Mycetocola** | ***Arthrospira*** | 0.770 | ***Pseudomonas*** | -0.671 |
|  | ***Clostridium*** | 0.754 |  |  |
|  | ***Kurthia*** | 0.728 |  |  |
|  |  |  |  |  |
| **Nocardioides** | ***Macrococcus*** | 0.798 |  |  |
|  | ***Methylobacterium*** | 0.827 |  |  |
|  | ***Renibacterium*** | 0.629 |  |  |
|  | ***Staphylococcus*** | 0.716 |  |  |
|  |  |  |  |  |
| **Okibacterium** | ***Agromyces*** | 0.847 | ***Chryseobacterium*** | -0.776 |
|  | ***Clavibacter*** | 0.790 | ***Deinococcus*** | -0.799 |
|  | ***Kineococcus*** | 1.000 | ***Hymenobacter*** | -0.787 |
|  | ***Leucobacter*** | 0.736 | ***Paenibacillus*** | -0.743 |
|  |  |  | ***Pedobacter*** | -0.672 |
|  |  |  | ***Rhodococcus*** | -0.767 |
|  |  |  | ***Spirosoma*** | -0.804 |
|  |  |  |  |  |
| **Oscillatoria** |  |  | ***Staphylococcus*** | -0.749 |
|  |  |  |  |  |
| **Paenibacillus** |  |  | ***Agromyces*** | -0.845 |
|  |  |  | ***Clavibacter*** | -0.872 |
|  |  |  | ***Cronobacter*** | -0.781 |
|  |  |  | ***Leifsonia*** | -0.873 |
|  |  |  | ***Leucobacter*** | -0.865 |
|  |  |  | ***Okibacterium*** | -0.743 |
|  |  |  | ***unclassified-(derived-from-Enterobacteriaceae)*** | -0.830 |
|  |  |  |  |  |
| **Pedobacter** | ***Chryseobacterium*** | 0.880 | ***Okibacterium*** | -0.672 |
|  | ***Flavobacterium*** | 0.688 | ***unclassified-(derived-from-Bacteria)*** | -1.000 |
|  | ***Kineococcus*** | 0.882 |  |  |
|  | ***Rhodococcus*** | 0.714 |  |  |
|  |  |  |  |  |
| **Pseudoalteromonas** | ***Arthrobacter*** | 0.761 | ***Bacillus*** | -0.756 |
|  | ***Macrococcus*** | 0.667 | ***Bifidobacterium*** | -0.843 |
|  | ***Shewanella*** | 0.861 | ***Clostridium*** | -0.772 |
|  | ***Sphingopyxis*** | 0.692 | ***Curtobacterium*** | -0.714 |
|  |  |  | ***Erwinia*** | 0.801 |
|  |  |  | ***Lactobacillus*** | -0.848 |
|  |  |  | ***unclassified-(derived-from-Oscillatoriales)*** | -0.821 |
| **Pseudomonas** | ***Acinetobacter*** | 0.805 | ***Citrobacter*** | -0.820 |
|  | ***Arthrobacter*** | 0.736 | ***Cronobacter*** | -0.767 |
|  | ***Flavobacterium*** | 0.710 | ***Mycetocola*** | -0.671 |
|  | ***Klebsiella*** | 0.699 |  |  |
|  |  |  |  |  |
| **Renibacterium** | ***Nocardioides*** | 0.629 | ***Kurthia*** | -0.686 |
|  | ***Sphingopyxis*** | 0.777 |  |  |
|  |  |  |  |  |
| **Rhizobium** | ***Curtobacterium*** | 0.775 | ***Azorhizobium*** | -0.670 |
|  | ***Leptolyngbya*** | 0.683 | ***Serratia*** | -0.777 |
|  | ***unclassified-(derived-from-Oscillatoriales)*** | 0.619 |  |  |
|  |  |  |  |  |
| **Rhodococcus** | ***Chryseobacterium*** | 0.805 | ***Kineococcus*** | -1.000 |
|  | ***Deinococcus*** | 0.846 | ***Okibacterium*** | -0.767 |
|  | ***Hymenobacter*** | 0.799 |  |  |
|  | ***Pedobacter*** | 0.714 |  |  |
|  | ***Spirosoma*** | 0.878 |  |  |
|  |  |  |  |  |
| **Riemerella** | ***Acinetobacter*** | 0.811 | ***Staphylococcus*** | -0.891 |
|  |  |  |  |  |
| **Rothia** | ***Lyngbya*** | 0.813 | ***Xanthomonas*** | -0.666 |
|  | ***unclassified-(derived-from-Alphaproteobacteria)*** | 0.833 |  |  |
|  |  |  |  |  |
| **Salmonella** | ***Staphylococcus*** | 0.746 |  |  |
|  |  |  |  |  |
| **Serratia** | ***Cronobacter*** | 0.775 | ***Enterococcus*** | -0.741 |
|  | ***Escherichia*** | 0.851 | ***Rhizobium*** | -0.777 |
|  | ***Leucobacter*** | 0.717 | ***Spirosoma*** | -0.786 |
|  |  |  |  |  |
| **Shewanella** | ***Arthrobacter*** | 0.952 | ***Arthrospira*** | -0.647 |
|  | ***Erwinia*** | 0.858 | ***Bacillus*** | -0.889 |
|  | ***Macrococcus*** | 0.731 | ***Bifidobacterium*** | -0.931 |
|  | ***Pseudoalteromonas*** | 0.861 | ***Clostridium*** | -0.819 |
|  | ***Sphingobacterium*** | 0.712 | ***Lactobacillus*** | -0.927 |
|  | ***Sphingopyxis*** | 0.840 | ***unclassified-(derived-from-Oscillatoriales)*** | -0.871 |
|  |  |  |  |  |
| **Sphingobacterium** | ***Deinococcus*** | 0.716 | ***Agromyces*** | -0.755 |
|  | ***Macrococcus*** | 0.651 | ***Candidatus-Aquiluna*** | -0.696 |
|  | ***Shewanella*** | 0.712 | ***Clavibacter*** | -0.795 |
|  |  |  | ***Kineococcus*** | -1.000 |
|  |  |  | ***Leifsonia*** | -0.822 |
|  |  |  | ***Leucobacter*** | -0.722 |
|  |  |  | ***Methylocella*** | -0.782 |
| **Sphingomonas** | ***Candidatus-Aquiluna*** | 0.753 |  |  |
|  | ***Klebsiella*** | 0.805 |  |  |
|  | ***unclassified-(derived-from-Alphaproteobacteria)*** | 1.000 |  |  |
|  | ***unclassified-(derived-from-Bacteria)*** | 1.000 |  |  |
|  |  |  |  |  |
| **Sphingopyxis** | ***Acidovorax*** | 0.829 | ***Bifidobacterium*** | -0.825 |
|  | ***Arthrobacter*** | 0.801 | ***Curtobacterium*** | -0.791 |
|  | ***Erwinia*** | 0.708 | ***Lactobacillus*** | -0.789 |
|  | ***Macrococcus*** | 0.748 | ***unclassified-(derived-from-Oscillatoriales)*** | -0.784 |
|  | ***Pseudoalteromonas*** | 0.692 |  |  |
|  | ***Renibacterium*** | 0.777 |  |  |
|  | ***Shewanella*** | 0.840 |  |  |
|  |  |  |  |  |
| **Spirosoma** | ***Chryseobacterium*** | 0.866 | ***Azorhizobium*** | -0.691 |
|  | ***Deinococcus*** | 0.840 | ***Clavibacter*** | -0.816 |
|  | ***Hymenobacter*** | 0.802 | ***Kineococcus*** | -1.000 |
|  | ***Rhodococcus*** | 0.878 | ***Leucobacter*** | -0.754 |
|  |  |  | ***Okibacterium*** | -0.804 |
|  |  |  | ***Serratia*** | -0.786 |
|  |  |  |  |  |
| **Staphylococcus** | ***Citrobacter*** | 0.694 | ***Exiguobacterium*** | -0.858 |
|  | ***Macrococcus*** | 0.753 | ***Flavobacterium*** | -0.741 |
|  | ***Methylobacterium*** | 0.784 | ***Oscillatoria*** | -0.749 |
|  | ***Nocardioides*** | 0.716 | ***Riemerella*** | -0.891 |
|  | ***Salmonella*** | 0.746 |  |  |
|  | ***Tetrasphaera*** | 0.894 |  |  |
|  |  |  |  |  |
| **Tetrasphaera** | ***Citrobacter*** | 0.701 | ***Acinetobacter*** | -0.798 |
|  |  |  | ***Exiguobacterium*** | -0.829 |
|  |  |  | ***Flavobacterium*** | -0.778 |
|  |  |  |  |  |
|  | ***Staphylococcus*** | 0.894 |  |  |
|  |  |  |  |  |
| **unclassified-(derived-from-Alphaproteobacteria)** | ***Acidovorax*** | 0.944 | ***Asaia*** | -0.912 |
|  | ***Lyngbya*** | 0.783 | ***Candidatus-Aquiluna*** | -0.825 |
|  | ***Rothia*** | 0.833 |  |  |
|  | ***Sphingomonas*** | 1.000 |  |  |
|  |  |  |  |  |
| **unclassified-(derived-from-Bacteria)** | ***Asaia*** | 1.000 | ***Acidovorax*** | -1.000 |
|  | ***Candidatus-Aquiluna*** | 1.000 | ***Flavobacterium*** | -1.000 |
|  | ***Leifsonia*** | 1.000 | ***Methylocella*** | -0.763 |
|  | ***Sphingomonas*** | 1.000 | ***Pedobacter*** | -1.000 |
|  |  |  | ***Chryseobacterium*** | -0.802 |
|  |  |  | ***Massilia*** | -0.770 |
|  |  |  | ***Paenibacillus*** | -0.830 |
|  |  |  |  |  |
| **unclassified-(derived-from-Oscillatoriales)** | ***Bacillus*** | 0.814 | ***Arthrobacter*** | -0.753 |
|  | ***Bifidobacterium*** | 0.948 | ***Erwinia*** | -0.796 |
|  | ***Candidatus-Aquiluna*** | 0.702 | ***Macrococcus*** | -0.717 |
|  | ***Clostridium*** | 0.810 | ***Pseudoalteromonas*** | -0.821 |
|  | ***Curtobacterium*** | 0.844 | ***Shewanella*** | -0.871 |
|  | ***Lactobacillus*** | 0.889 | ***Sphingopyxis*** | -0.784 |
|  | ***Leptolyngbya*** | 0.821 |  |  |
|  | ***Rhizobium*** | 0.619 |  |  |
|  | ***Xenococcus*** | 0.787 |  |  |
|  |  |  |  |  |
| **unclassified-(derived-from-unclassified-sequences)** |  |  | ***Bacillus*** | -0.896 |
|  |  |  | ***Methylobacterium*** | -0.884 |
|  |  |  | ***Microcoleus*** | -0.950 |
|  |  |  |  |  |
| **Xanthomonas** | ***Asaia*** | 0.798 | ***Rothia*** | -0.666 |
|  |  |  |  |  |
|  |  |  |  |  |
| **Xenococcus** | ***Asaia*** | 0.901 | ***Acidovorax*** | -0.729 |
|  | ***Bifidobacterium*** | 0.672 | ***Micrococcus*** | -0.646 |
|  | ***Candidatus-Aquiluna*** | 0.635 |  |  |
|  | ***Enterococcus*** | 0.782 |  |  |
|  | ***Leptolyngbya*** | 0.923 |  |  |
|  | ***unclassified-(derived-from-Oscillatoriales)*** | 0.787 |  |  |

**Supplementary Table 5. Population size of epiphytic bacteria (Log CFU g^_1^) on phyllosphere of 15 and 30 days aged rice genotypes grown in Mountain zone**

| **Isolate** | **Colony morphology** | **PRR78** | **Pusa 1602** | **Isolate** | **Colony morphology** | **PRR78** | **Pusa 1602** |
| --- | --- | --- | --- | --- | --- | --- | --- |
| 15P1 | Punctiform, red without hallow | 5.91 | 6.00 | 30P1 | Punctiform, red without hallow | 5.83 | 5.83 |
| 15P2 | Punctiform, red with hallow | 6.27 | 6.24 | 30P2 | Punctiform, red with hallow | 5.62 | 5.64 |
| 15P3 | Small dot like, dark red | 6.16 | 5.59 | 30P3 | Small dot like, dark red | 5.68 | 5.48 |
| 15P4 | Small dot like, dark red with hallow | 4.39 | 2.48 | 30P4 | Small dot like, dark red with hallow | 6.40 | 5.67 |
| 15P5 | Small dark red with yellow hallow | 1.47 | 4.01 | 30P5 | Small dark red with yellow hallow | 5.33 | 3.74 |
| 15P6 | Small dark red with while hallow | 5.35 | 4.10 | 30P6 | Small dark red with cream margin | 3.18 | 5.09 |
| 15P7 | Small, pink red with 3D structure inside media | 4.76 | 2.56 | 30P7 | Small, maroon red, round with smooth margin | 2.64 | 0.00 |
| 15P8 | Small dark maroon with white hallow, convex surface | 5.01 | 4.40 | 30P8 | Small, pink red with 3D structure inside media | 1.91 | 5.02 |
| 15P9 | Small point center with a large cream margin | 3.53 | 0.00 | 30P9 | Moderate, orange red, rough with white filiform margin | 2.62 | 3.11 |
| 15P10 | Moderate, red center with white hallow and entire margin | 3.53 | 2.00 | 30P10 | Moderate, white circular with a red dot like center | 2.90 | 0.00 |
| 15P11 | Moderate, translucent white slimy, irregular shape | 5.21 | 2.00 | 30P11 | Moderate, slimy white, oval to round with entire margin | 3.45 | 0.00 |
| 15P12 | Moderate, maroon red, convex slimy surface | 5.79 | 1.43 | 30P12 | Large, creamy white with red center, smooth margin | 1.43 | 1.47 |
| 15P13 | Moderate rough surface oval with irregular margin | 3.88 | 2.97 | 30P13 | Moderate, maroon red, convex slimy surface with irregular margin | 1.47 | 3.05 |
| 15P14 | Moderate maroon red, oval to round with white smooth margin | 2.00 | 2.48 | 30P14 | Large, pinkish red with filiform margin | 5.32 | 4.22 |
| 15P15 | Large, flat surface with irregular white margin | 1.47 | 3.50 | 30P15 | Large, slimy rose red, wavy margin | 0.00 | 2.46 |
| - | - | - | - | 30P16 | Large, yellow, flat surface with irregular margin | 0.00 | 3.94 |
| - | - | - | - | 30P17 | Small 3D light red submerged inside the media | 3.08 | 1.47 |
| - | - | - | - | 30P18 | Small, rose red with rough surface, irregular margin | 1.95 | 2.90 |
| - | - | - | - | 30P19 | Small, dark red, submerged with a disc like structure | 2.93 | 2.52 |
| - | - | - | - | 30P20 | Small, violate red, submerged, flat on the bottom of plate | 1.91 | 3.05 |
| - | - | - | - | 30P21 | Moderate, red center with white background, circular with smooth margin | 3.38 | 1.47 |
| - | - | - | - | 30P22 | Small red center with yellow margin, entire circular | 1.43 | 0.96 |
| - | - | - | - | 30P23 | Large, rose red big center with white background and filiform margin | 3.48 | 2.90 |
|  | **Mean (Cultivar)** | **4.313** | **3.317** |  | **Mean (Cultivar)** | **3.127** | **3.042** |
|  |  | Cultivar | Bacteria |  |  | Cultivar | Bacteria |
|  | *F cal* | 23.85 | 12.58 |  | *F cal* | 0.25 | 15.21 |
|  | *F tab* | 3.88 | 1.73 |  | *F tab* | 3.8 | 1.57 |

CFU/ml= (Number of colonies) X (Dilution factor)/Volume plated in mL

**Supplementary Table 6. Population size of epiphytic bacteria (Log CFU g^-1^) on phyllosphere of rice genotypes grown in Island zone**

| **Isolate** | **Colony morphology** | **PRR78** | **Pusa 1602** | **Isolate** | **Colony morphology** | **PRR78** | **Pusa 1602** |
| --- | --- | --- | --- | --- | --- | --- | --- |
| 15A1 | Punctiform, light red | 5.11 | 6.55 | 30A1 | Punctiform, light red | 6.75 | 6.36 |
| 15A2 | Punctiform, dark red | 6.10 | 6.00 | 30A2 | Punctiform, dark red | 6.04 | 5.90 |
| 15A3 | Punctiform, light red with white hallow | 3.87 | 5.41 | 30A3 | Punctiform, light red with white hallow | 5.71 | 5.77 |
| 15A4 | Moderate, pink center like a dot and white periphery | 2.72 | 4.49 | 30A4 | Small, wine red, | 3.41 | 3.29 |
| 15A5 | Moderate, dark red center like a dot and white periphery | 5.34 | 4.28 | 30A5 | Small, pinkish white disc like 3D structure submerged in media | 3.84 | 3.16 |
| 15A6 | Small orange center with yellow hallow | 0.00 | 3.17 | 30A6 | Small submerged close to bottom of plate, violate red with entire margin | 2.69 | 4.17 |
| 15A7 | Small red center with white hallow | 4.21 | 2.42 | 30A7 | Small submerged close to bottom of plate, pinkish red with filiform margin | 3.47 | 1.47 |
| 15A8 | Moderate, cherry red with double ring structure | 0.00 | 2.49 | 30A8 | Small, dark red, with metallic shine, entire | 5.20 | 4.10 |
| 15A9 | Moderate dark red with raised center | 0.00 | 2.60 | 30A9 | Small, orange yellow round with smooth margin | 5.57 | 4.15 |
| 15A10 | Moderate, blood red center with creamy hallow and entire margin | 3.42 | 2.51 | 30A10 | Small, light yellow round with smooth margin | 4.41 | 2.48 |
| 15A11 | Moderate, wine red with metallic shine, round smooth margin | 5.04 | 0.00 | 30A11 | Small white with pink dot at the center | 4.32 | 0.00 |
| 15A12 | Moderate, opaque white, irregular, with filamentous margin | 1.98 | 4.97 | 30A12 | Moderate, candy red slimy round to oval with entire margin | 1.87 | 0.00 |
| 15A13 | Large, creamy white, filiform border | 2.39 | 3.19 | 30A13 | Moderate, dark wine red, round, with metallic shine and entire margin | 4.15 | 2.95 |
| 15A14 | Large, slimy white with wavy margin | 1.50 | 2.46 | 30A14 | Moderate light pink watery colony with concentric rings | 2.93 | 3.98 |
| 15A15 | Large, light red, oval to round with smooth margin | 2.03 | 2.48 | 30A15 | Moderate, red center with large yellow hallow | 2.63 | 0.00 |
| 15A16 | Small wine red with entire smooth margin | 2.49 | 0.00 | 30A16 | Large, opaque white, rhizoidal margin with red shades at the base | 0.00 | 1.91 |
| 15A17 | Moderate glossy yellow, oval with entire margin | 5.21 | 0.00 | 30A17 | Large, maroon red with creamy white hallow | 0.00 | 2.42 |
| 15A18 | Small, rose red with 3 dimensional structure submerged in the media | 5.75 | 0.00 | 30A18 | Moderate cherry red with yellow white hallow | 0.00 | 3.47 |
| - | - | - | - | 30A19 | Small boat shape dark red with brown hallow | 2.42 | 1.95 |
| - | - | - | - | 30A20 | Moderate, violate red, entire | 4.18 | 1.95 |
| - | - | - | - | 30A21 | Small, slimy colony with irregular margin | 4.64 | 3.50 |
| - | - | - | - | 30A22 | Large creamy white, wavy margin | 2.51 | 3.98 |
|  | **Mean (Cultivar)** | 3.175 | 2.945 |  | **Mean (Cultivar)** | 3.488 | 3.043 |
|  |  | Cultivar | Bacteria |  |  | Cultivar | Bacteria |
|  | *F cal* | 1.54 | 14.14 |  | *F cal* | 7.37 | 17.35 |
|  | *F tab* | 3.87 | 1.66 |  | *F tab* | 3.87 | 1.58 |

CFU/ml= (Number of colonies) X (Dilution factor)/Volume plated in mL

**Supplementary Table 7. Identification of bacterial isolates by 16S rRNA gene sequencing**

| Sequence ID | Organism | Seq. length (bp) | Host | Geographical origin | GenBank Accession |
| --- | --- | --- | --- | --- | --- |
| OsEp_Plm_30P1 | *Acidovorax avenae* | 1433 | PRR78 & Pusa 1602 | Palampur (HP) | MT367817 |
| OsEp_Plm_30P6 | *Acidovorax avenae* | 1396 | PRR78 & Pusa 1602 | Palampur (HP) | MT367820 |
| OsEp_Plm_30P23 | *Acidovorax avenae* | 1378 | PRR78 & Pusa 1602 | Palampur (HP) | MT367833 |
| OsEp_Plm_30P11 | *Acinetobacter baumannii* | 1430 | PRR78 | Palampur (HP) | MT367824 |
| OsEp_Plm_30P17 | *Acinetobacter baumannii* | 1401 | PRR78 & Pusa 1602 | Palampur (HP) | MT367827 |
| OsEp_A&N_30A17 | *Acinetobacter junii* | 1386 | Pusa 1602 | Port Blair (A&N) | MT367859 |
| OsEp_Plm_30P2 | *Acinetobacter soli* | 1419 | PRR78 & Pusa 1602 | Palampur (HP) | MT394056 |
| OsEp_Plm_30P4 | *Acinetobacter soli* | 1429 | PRR78 & Pusa 1602 | Palampur (HP) | MT367819 |
| OsEp_Plm_30P22 | *Acinetobacter soli* | 1417 | PRR78 & Pusa 1602 | Palampur (HP) | MT367832 |
| OsEp_Plm_30P19 | *Agrobacterium larrymoorei* | 1359 | PRR78 & Pusa 1602 | Palampur (HP) | MT367829 |
| OsEp_A&N_30A11 | *Aureimonas phyllosphaerae* | 1390 | PRR78 | Port Blair (A&N) | MT367855 |
| OsEp_Plm_30P7 | *Aureimonas* sp. | 1369 | PRR78 | Palampur (HP) | MT367821 |
| OsEp_Plm_15P1 | *Curtobacterium albidum* | 1391 | PRR78 & Pusa 1602 | Palampur (HP) | MT367807 |
| OsEp_Plm_30P20 | *Curtobacterium albidum* | 1401 | PRR78 & Pusa 1602 | Palampur (HP) | MT367830 |
| OsEp_A&N_30A1 | *Curtobacterium citreum* | 1395 | PRR78 & Pusa 1602 | Port Blair (A&N) | MT367846 |
| OsEp_Plm_15P7 | *Curtobacterium luteum* | 1390 | PRR78 & Pusa 1602 | Palampur (HP) | MT367812 |
| OsEp_Plm_30P9 | *Curtobacterium luteum* | 1393 | PRR78 & Pusa 1602 | Palampur (HP) | MT367822 |
| OsEp_Plm_30P16 | *Enterobacter asburiae* | 1410 | Pusa 1602 | Palampur (HP) | MT367826 |
| OsEp_A&N_30A22 | *Enterobacter asburiae* | 1406 | PRR78 & Pusa 1602 | Port Blair (A&N) | MT367864 |
| OsEp_Plm_30P18 | *Enterobacter cloacae* | 1425 | PRR78 & Pusa 1602 | Palampur (HP) | MT367828 |
| OsEp_A&N_15A7 | *Enterobacter cloacae* | 1409 | PRR78 & Pusa 1602 | Port Blair (A&N) | MT367840 |
| OsEp_A&N_30A20 | *Enterobacter mori* | 1409 | PRR78 & Pusa 1602 | Port Blair (A&N) | MT367862 |
| OsEp_A&N_15A12 | *Enterobacter sichuanensis* | 1404 | PRR78 & Pusa 1602 | Port Blair (A&N) | MT367844 |
| OsEp_A&N_15A5 | *Erwinia tasmaniensis* | 1412 | PRR78 & Pusa 1602 | Port Blair (A&N) | MT367838 |
| OsEp_Plm_15P3 | *Exiguobacterium acetylicum* | 1438 | PRR78 & Pusa 1602 | Palampur (HP) | MT367809 |
| OsEp_Plm_30P14 | *Exiguobacterium indicum* | 1431 | PRR78 & Pusa 1602 | Palampur (HP) | MT367825 |
| OsEp_A&N_30A4 | *Exiguobacterium indicum* | 1413 | PRR78 & Pusa 1602 | Port Blair (A&N) | MT367849 |
| OsEp_A&N_30A6 | *Exiguobacterium indicum* | 1430 | PRR78 & Pusa 1602 | Port Blair (A&N) | MT367851 |
| OsEp_A&N_15A2 | *Microbacterium* sp. | 1387 | PRR78 & Pusa 1602 | Port Blair (A&N) | MT367835 |
| OsEp_A&N_30A2 | *Microbacterium testaceum* | 1409 | PRR78 & Pusa 1602 | Port Blair (A&N) | MT367847 |
| OsEp_A&N_15A1 | *Micrococcus luteus* | 1400 | PRR78 & Pusa 1602 | Port Blair (A&N) | MT367834 |
| OsEp_A&N_15A8 | *Pantoea agglomerans* | 1418 | Pusa 1602 | Port Blair (A&N) | MT367841 |
| OsEp_A&N_30A14 | *Pantoea agglomerans* | 1408 | PRR78 & Pusa 1602 | Port Blair (A&N) | MT367857 |
| OsEp_A&N_30A21 | *Pantoea agglomerans* | 1413 | PRR78 & Pusa 1602 | Port Blair (A&N) | MT367863 |
| OsEp_Plm_15P9 | *Pantoea ananatis* | 1410 | PRR78 | Palampur (HP) | MT367813 |
| OsEp_Plm_30P3 | *Pantoea ananatis* | 1419 | PRR78 & Pusa 1602 | Palampur (HP) | MT367818 |
| OsEp_Plm_30P21 | *Pantoea ananatis* | 1405 | PRR78 & Pusa 1602 | Palampur (HP) | MT367831 |
| OsEp_A&N_15A10 | *Pantoea ananatis* | 1401 | PRR78 & Pusa 1602 | Port Blair (A&N) | MT367843 |
| OsEp_A&N_30A5 | *Pantoea ananatis* | 1402 | PRR78 & Pusa 1602 | Port Blair (A&N) | MT367850 |
| OsEp_A&N_30A8 | *Pantoea ananatis* | 1403 | PRR78 & Pusa 1602 | Port Blair (A&N) | MT367852 |
| OsEp_A&N_30A19 | *Pantoea ananatis* | 1408 | PRR78 & Pusa 1602 | Port Blair (A&N) | MT367861 |
| OsEp_A&N_30A18 | *Pantoea dispersa* | 1412 | Pusa 1602 | Port Blair (A&N) | MT367860 |
| OsEp_Plm_15P14 | *Pantoea eucrina* | 1421 | PRR78 & Pusa 1602 | Palampur (HP) | MT367816 |
| OsEp_Plm_30P10 | *Pantoea eucrina* | 1414 | PRR78 | Palampur (HP) | MT367823 |
| OsEp_A&N_15A4 | *Pantoea eucrina* | 1409 | PRR78 & Pusa 1602 | Port Blair (A&N) | MT367837 |
| OsEp_A&N_15A9 | *Pantoea* sp. | 1402 | Pusa 1602 | Port Blair (A&N) | MT367842 |
| OsEp_A&N_15A15 | *Pantoea* sp. | 1400 | PRR78 & Pusa 1602 | Port Blair (A&N) | MT367845 |
| OsEp_Plm_15P6 | *Pseudomonas oryzihabitans* | 1398 | PRR78 & Pusa 1602 | Palampur (HP) | MT367811 |
| OsEp_Plm_15P12 | *Pseudomonas parafulva* | 1407 | PRR78 & Pusa 1602 | Palampur (HP) | MT367815 |
| OsEp_A&N_15A6 | *Pseudomonas psychrotolerans* | 1383 | Pusa 1602 | Port Blair (A&N) | MT367839 |
| OsEp_A&N_30A13 | *Pseudomonas psychrotolerans* | 1396 | PRR78 & Pusa 1602 | Port Blair (A&N) | MT367856 |
| OsEp_Plm_15P11 | *Pseudomonas putida* | 1401 | PRR78 & Pusa 1602 | Palampur (HP) | MT367814 |
| OsEp_A&N_15A3 | *Sphingomonas paucimobilis* | 1390 | PRR78 & Pusa 1602 | Port Blair (A&N) | MT367836 |
| OsEp_A&N_30A9 | *Sphingomonas paucimobilis* | 1377 | PRR78 & Pusa 1602 | Port Blair (A&N) | MT367853 |
| OsEp_Plm_15P2 | *Sphingomonas pseudosanguinis* | 1389 | PRR78 & Pusa 1602 | Palampur (HP) | MT367808 |
| OsEp_A&N_30A10 | *Sphingomonas pseudosanguinis* | 1378 | PRR78 & Pusa 1602 | Port Blair (A&N) | MT367854 |
| OsEp_Plm_15P5 | *Sphingomonas* sp. | 1378 | PRR78 & Pusa 1602 | Palampur (HP) | MT367810 |
| OsEp_A&N_30A15 | *Sphingomonas* sp. | 1362 | PRR78 | Port Blair (A&N) | MT367858 |
| OsEp_A&N_30A3 | *Sphingomonas yabuuchiae* | 1362 | PRR78 & Pusa 1602 | Port Blair (A&N) | MT367848 |

**Supplementary Table 8. Analysis of nature of BVC mediated mycelial inhibition of *Magnaporthe oryzae***

| **Isolate** | **Bacterial Species** | **Inhibition by BVC (%)** | **Re-growth of *Magnaporthe oryzae* (%)** | **Nature of BVC** |
| --- | --- | --- | --- | --- |
| OsEp-Plm-30P11 | *Acinetobacter baumannii* | 25.7 | 74.3 | Fungistatic |
| OsEp-Plm-30P17 | *Acinetobacter baumannii* | 17.1 | 82.9 | Fungistatic |
| OsEp-Plm-30P4 | *Acinetobacter soli* | 9.5 | 90.5 | Fungistatic |
| OsEp-Plm-30P7 | *Aureimonas* sp. | 11.4 | 88.6 | Fungistatic |
| **OsEp-AN-30A14** | ***Pantoea agglomerans*** | **100.0** | **0.0** | **Fungicidal** |
| **OsEp-Plm-15P9** | ***Pantoea ananatis*** | **100.0** | **0.0** | **Fungicidal** |
| **OsEp-Plm-30P21** | ***Pantoea ananatis*** | **100.0** | **0.0** | **Fungicidal** |
| **OsEp-AN-30A5** | ***Pantoea ananatis*** | **100.0** | **0.0** | **Fungicidal** |
| **OsEp-AN-30A8** | ***Pantoea ananatis*** | **100.0** | **0.0** | **Fungicidal** |
| OsEp-AN-30A18 | *Pantoea dispersa* | 21.9 | 78.1 | Fungistatic |
| OsEp-Plm-15P14 | *Pantoea eucrina* | 31.4 | 68.6 | Fungistatic |
| OsEp-Plm-30P10 | *Pantoea eucrina* | 24.8 | 75.2 | Fungistatic |
| OsEp-AN-15A4 | *Pantoea eucrina* | 58.1 | 41.9 | Fungistatic |
| OsEp-Plm-15P12 | *Pseudomonas parafulva* | 69.5 | 30.5 | Fungistatic |
| OsEp-Plm-15P11 | *Pseudomonas putida* | 5.7 | 94.3 | Fungistatic |
|  | Mock | 0.0 | 100.0 |  |
|  | C.D. | 10.93 | 3.79 |  |
|  | SE(m) | 3.79 | 5.37 |  |
|  | SE(d) | 5.37 | 10.93 |  |
|  | C.V. (%) | 13.56 | 12.75 |  |
|  | F (calc.) | 110.82 | 110.82 |  |
|  | F (tab.) | 1.99 | 1.99 |  |

**Supplementary Table 9. qPCR analysis of transcriptional response of defense genes in rice seedlings upon phyllobacterization**

| ***Gene*** | | **Fold change** | | | |
| --- | --- | --- | --- | --- | --- |
|  |  | **Time line** | | | |
|  | **24 h** | | **48 h** | | **72 h** |
| ***OsPAD4*** | |  | |  |  |
| *Pantoea ananatis* OsEp-Plm-30P3 | | 0.86 | | **1.165** | **1.04** |
| *Aureimonas* sp. OsEp-Plm-30P7 | | 0.56 | | **1.005** | **1.065** |
| *Pantoea eucrina* OsEp-Plm-30P10 | | **1.03** | | **1.075** | 1 |
| *Pantoea ananatis* OsEp-Plm-30P21 | | 0.835 | | 0.77 | 0.71 |
| *Pseudomonas putida* OsEp-Plm-15P11 | | 0.855 | | 0.63 | 1 |
| *Pantoea ananatis* OsEp-AN-30A8 | | **1.54** | | **3.51** | 0.655 |
| ***OsEDS1*** | |  | |  |  |
| *Pantoea ananatis* OsEp-Plm-30P3 | | 0.91 | | 0.845 | **1.085** |
| *Aureimonas sp.* OsEp-Plm-30P7 | | 0.765 | | 0.685 | **1.235** |
| *Pantoea eucrina* OsEp-Plm-30P10 | | 0.825 | | **1.145** | 0.905 |
| *Pantoea ananatis* OsEp-Plm-30P21 | | 0.78 | | 0.6 | **1.165** |
| *Pseudomonas putida* OsEp-Plm-15P11 | | 0.81 | | 0.645 | **1.395** |
| *Pantoea ananatis* OsEp-AN-30A8 | | 1 | | 0.915 | **1.12** |
| ***OsNPR1*** | |  | |  |  |
| *Pantoea ananatis* OsEp-Plm-30P3 | | 0.815 | | 0.78 | **1.18** |
| *Aureimonas sp.* OsEp-Plm-30P7 | | 0.53 | | 0.48 | **1.275** |
| *Pantoea eucrina* OsEp-Plm-30P10 | | 0.785 | | 0.495 | **1.03** |
| *Pantoea ananatis* OsEp-Plm-30P21 | | 0.72 | | 0.455 | **1.31** |
| *Pseudomonas putida* OsEp-Plm-15P11 | | 0.675 | | 0.535 | **1.505** |
| *Pantoea ananatis* OsEp-AN-30A8 | | 0.94 | | 0.805 | 0.9 |
| ***OsPDF2.2*** | |  | |  |  |
| *Pantoea ananatis* OsEp-Plm-30P3 | | 0.51 | | **1.435** | **1.465** |
| *Aureimonas sp.* OsEp-Plm-30P7 | | 0.605 | | 0.845 | **1.1** |
| *Pantoea eucrina* OsEp-Plm-30P10 | | **1.42** | | 0.76 | **1.07** |
| *Pantoea ananatis* OsEp-Plm-30P21 | | 0.545 | | 0.88 | **1.05** |
| *Pseudomonas putida* OsEp-Plm-15P11 | | 0.6 | | 0.745 | **1.095** |
| *Pantoea ananatis* OsEp-AN-30A8 | | 0.58 | | 0.925 | **1.09** |
| ***OsCERK1*** | |  | |  |  |
| *Pantoea ananatis* OsEp-Plm-30P3 | | 0.7 | | 0.94 | 0.665 |
| *Aureimonas sp.* OsEp-Plm-30P7 | | 0.48 | | 0.825 | **1.22** |
| *Pantoea eucrina* OsEp-Plm-30P10 | | 0.615 | | 0.655 | 0.91 |
| *Pantoea ananatis* OsEp-Plm-30P21 | | 0.5 | | 0.595 | 1 |
| *Pseudomonas putida* OsEp-Plm-15P11 | | 0.46 | | **1.055** | **1.43** |
| *Pantoea ananatis* OsEp-AN-30A8 | | **1.06** | | **3.735** | 0.845 |
| ***OsCEBiP*** | |  | |  |  |
| *Pantoea ananatis* OsEp-Plm-30P3 | | **1.15** | | **1.695** | **1.225** |
| *Aureimonas* sp. OsEp-Plm-30P7 | | **2.55** | | **8.235** | **5.075** |
| *Pantoea eucrina* OsEp-Plm-30P10 | | **1.22** | | **1.7** | 0.945 |
| *Pantoea ananatis* OsEp-Plm-30P21 | | **1.36** | | **1.965** | **1.14** |
| *Pseudomonas putida* OsEp-Plm-15P11 | | **1.085** | | **2.425** | **1.705** |
| *Pantoea ananatis* OsEp-AN-30A8 | | **1.415** | | **4.59** | **1.275** |
| ***OsFMO*** | |  | |  |  |
| *Pantoea ananatis* OsEp-Plm-30P3 | | **1.794** | | **1.168** | 0.836 |
| *Aureimonas sp.* OsEp-Plm-30P7 | | **1.350** | | 0.834 | 0.743 |
| *Pantoea eucrina* OsEp-Plm-30P10 | | **1.453** | | 0.637 | 0.616 |
| *Pantoea ananatis* OsEp-Plm-30P21 | | 0.994 | | 0.569 | 0.559 |
| *Pseudomonas putida* OsEp-Plm-15P11 | | **1.298** | | 0.754 | 0.782 |
| *Pantoea ananatis* OsEp-AN-30A8 | | **2.333** | | 0.633 | 0.554 |
| ***OsPR1.1*** | |  | |  |  |
| *Pantoea ananatis* OsEp-Plm-30P3 | | 0.947 | | 0.858 | **1.284** |
| *Aureimonas sp.* OsEp-Plm-30P7 | | **1.252** | | 0.469 | **2.109** |
| *Pantoea eucrina* OsEp-Plm-30P10 | | **1.643** | | 0.521 | **1.149** |
| *Pantoea ananatis* OsEp-Plm-30P21 | | **1.565** | | 0.494 | **1.614** |
| *Pseudomonas putida* OsEp-Plm-15P11 | | **1.797** | | 0.680 | **1.861** |
| *Pantoea ananatis* OsEp-AN-30A8 | | **1.416** | | 0.561 | **1.580** |

***Bold= Up- regulated; Red font= Significant up-regulation**

1. *OsCEBiP* was found induced in all time points by bacterization; significant induction by *Aureimonas sp.* OsEp-Plm-30P7 for all three time points and *Pseudomonas putida* OsEp-Plm-15P11 or  *Pantoea ananatis* OsEp-AN-30A8 48 hour post bacterization
2. *OsPR1.1* was also found induced by 72 hour post bacterization with significant induction by *Aureimonas* sp. OsEp-Plm-30P7
3. *OsNPR1* and *OsPDF2.2* showed induction at 72 hour post inoculation for all the bacterial treatments.
4. Other genes induced were *OsFMO* in *Pantoea ananatis* OsEp-AN-30A8, *OsCERK1* and *OsPAD4* in *Pantoea ananatis* OsEp-AN-30A8
