# Supplementary material for: Deciphering core phyllomicrobiome assemblage on rice genotypes grown in contrasting agroclimatic zones: implications for phyllomicrobiome engineering against blast disease
Source: Environ Microbiome. 2022 May 26;17:28. doi: 10.1186/s40793-022-00421-5 (PMC9134649; doi:10.1186/s40793-022-00421-5)
Supplement: Supplementary file 2 — Additional file 2. Fig. S1. Identification of biomarkers based on the linear discriminant analysis (LDA) and effect size (LEfSe) pipeline; (A) Two genotypes (PRR78 and Pusa1602); (B) Two locations (Palampur and Port Blair). Fig. S2. Network analysis of rice phyllosphere microbiome using SparCC correlation coefficients (Normal group). Fig. S3. Extended error bar plot at various taxonomic hierarchy levels for phyllomicrobiome of rice genotypes, PRR78 and Pusa1602. Fig. 4. Extended error bar plot at various taxonomic hierarchy levels for phyllomicrobiome of rice grown in Palampur, Himachal Pradesh and Port Blair, Andaman Island. Fig. 5. Relative abundance of phyllosphere bacterial communities on rice genotypes grown in two agroclimatic zones of India. Fig. S6. Relative abundance of phyllosphere bacterial communities at genus level on two rice genotypes representing contrasting agroclimatic zones of India. Fig. 7. BOX PCR fingerprinting of cultured bacterial isolates of rice phyllosphere; M: DNA size marker; Lanes: Isolates of bacteria isolated from the phyllosphere of rice leaf. Fig. 8. Amplification of 16S rRNA of bacterial isolates of rice phyllosphere. Fig. 9a. Colonies of cultured Acidovorax species from rice phyllomicrobiome. Fig. S9b. Colonies of cultured Acinetobacter species from rice phyllomicrobiome. Fig. 9c. Colonies of cultured Agrobacterium species from rice phyllomicrobiome. Fig. S9d. Colonies of cultured Aureimonas species from rice phyllomicrobiome. Fig. S9e. Colonies of cultured Curtobacterium species from rice phyllomicrobiome. Fig. 9f. Colonies of cultured Enterobacter species from rice phyllomicrobiome. Fig. S9g. Colonies of cultured Erwinia species from rice phyllomicrobiome. Fig. 9h. Colonies of cultured Exiguobacterium species from rice phyllomicrobiome. Fig. S9i. Colonies of cultured Microbacterium species from rice phyllomicrobiome. Fig. S9j. Colonies of cultured Micrococcus species from rice phyllomicrobiome. Fig. S9k. Colonies of cultured Pan [file 40793_2022_421_MOESM2_ESM.docx]

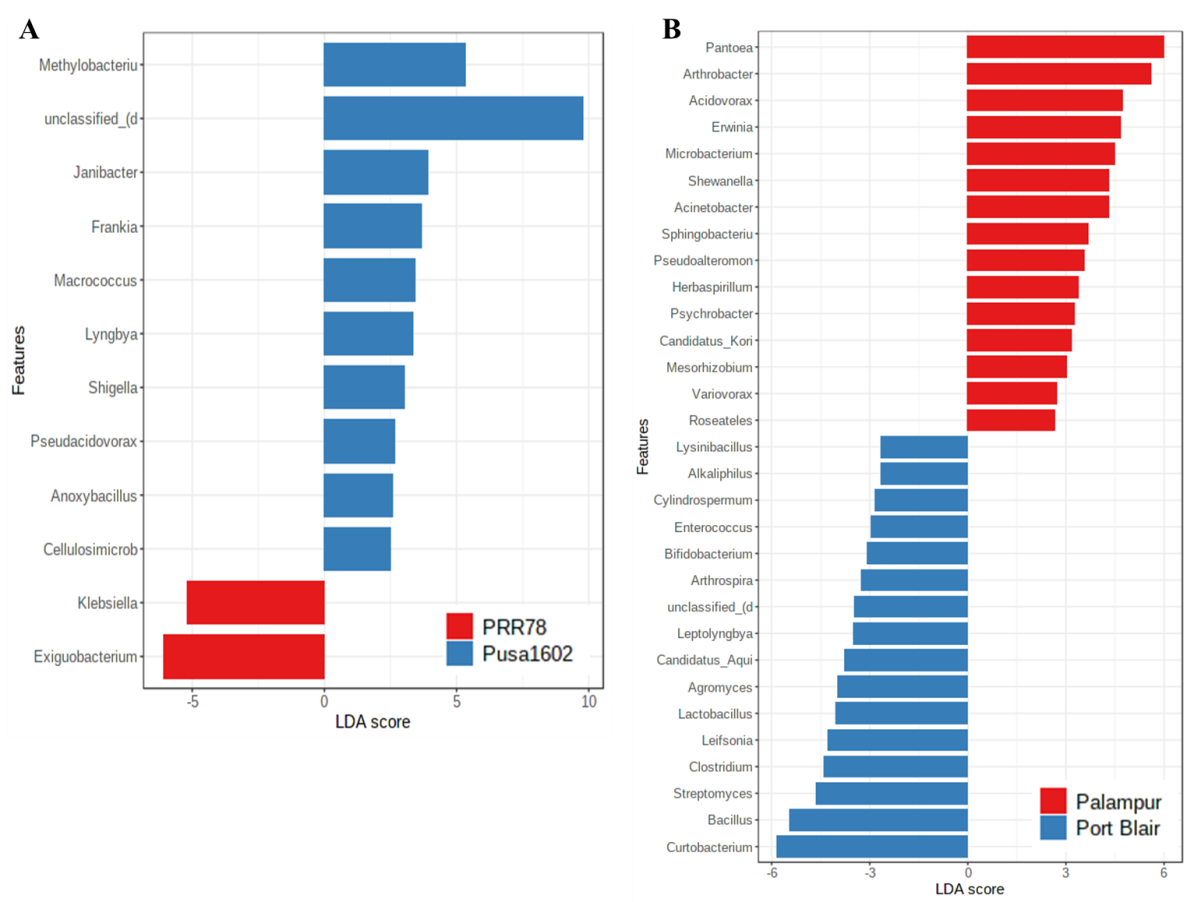


**Supplementary Fig. 1. Identification of biomarkers based on the linear discriminant analysis (LDA) and effect size (LEfSe) pipeline;** (A) Two genotypes i.e. PRR78 and Pusa1602; (B) Two location i.e. Palampur and Port Blair


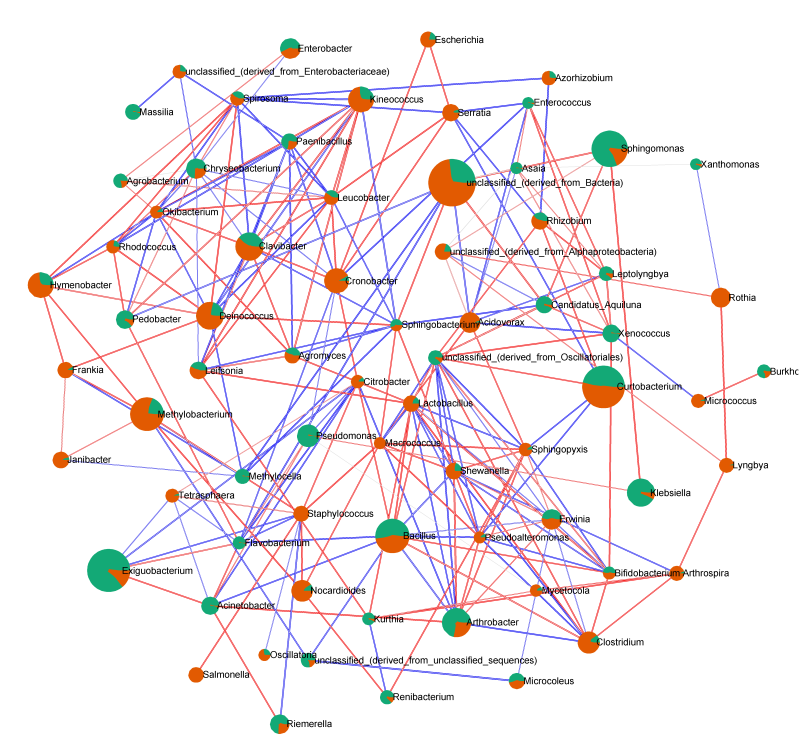
(A)
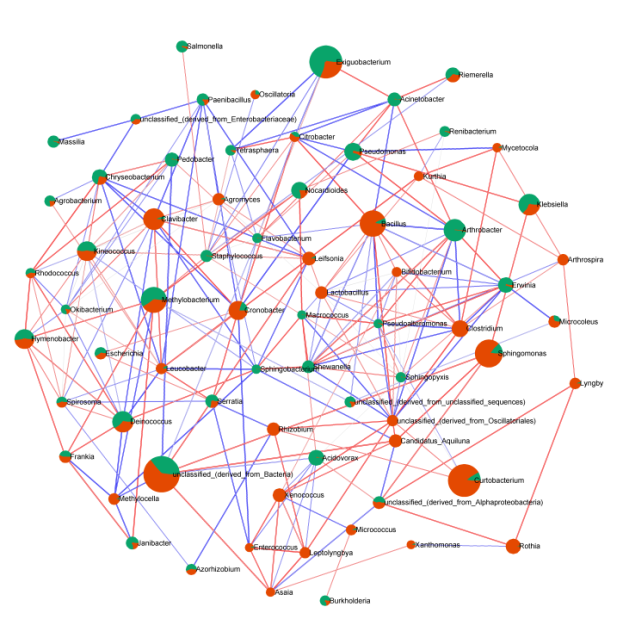
(B)

**Supplementary Fig. 2. Network analysis of rice phyllospheric microbiome using SparCC correlation coefficients (Normal group).** The figure shows networks between abundant sequences at the genus level built from SparCC correlation coefficients. Network plotted with correlation coefficient R^2^>0.6 or <0.6 and P<0.5. The edges were marked with red line which indicates the positive while blue indicates negative correlation. Genera were filtered with <20 read and then normalized using total scaling sum (TSS) method. The nodes represent genera of bacteria; different nodes size indicate the taxa size; (**A) Two genotypes i.e. PRR78 (Green) and Pusa1602 (Orange); (B) Two location i.e. Palampur (Green) and Port Blair (Orange).**

| **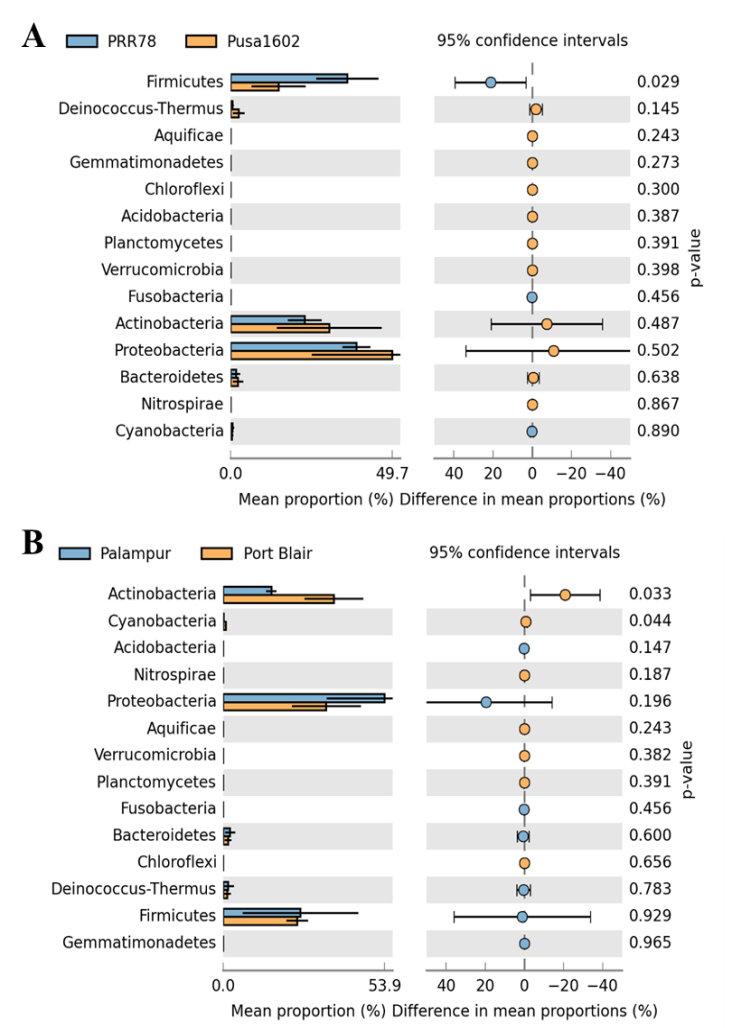** | **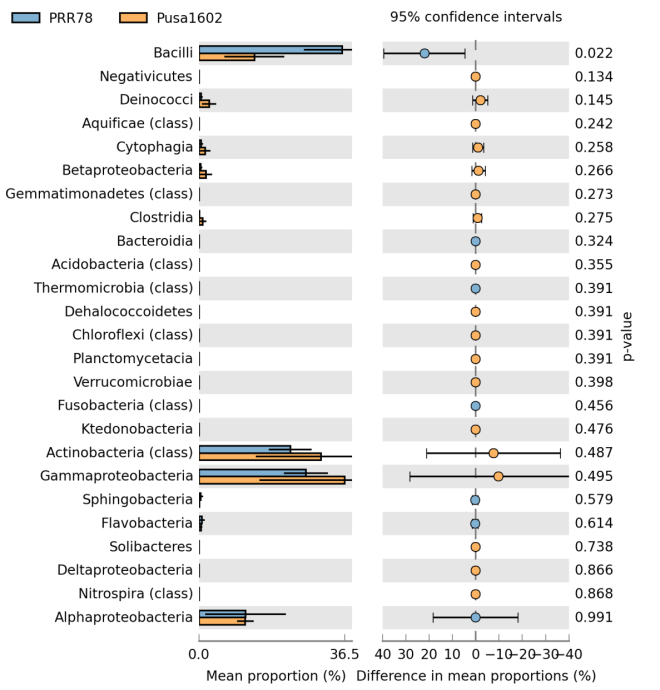** |
| --- | --- |
| **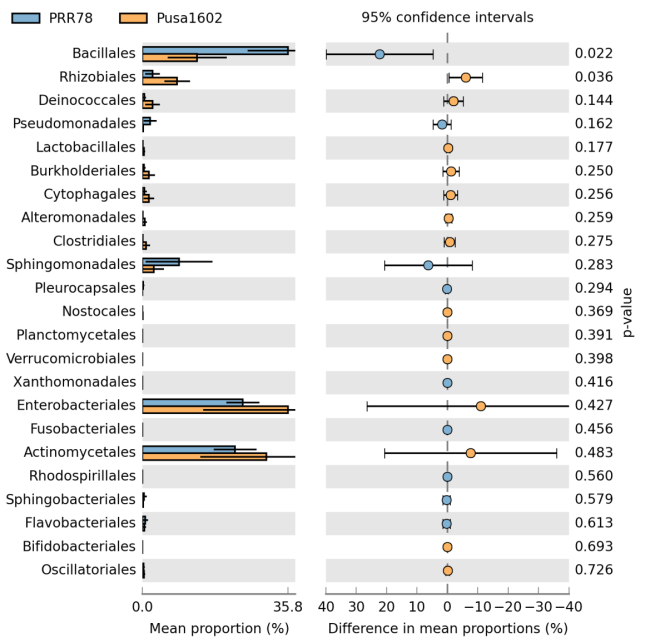** | **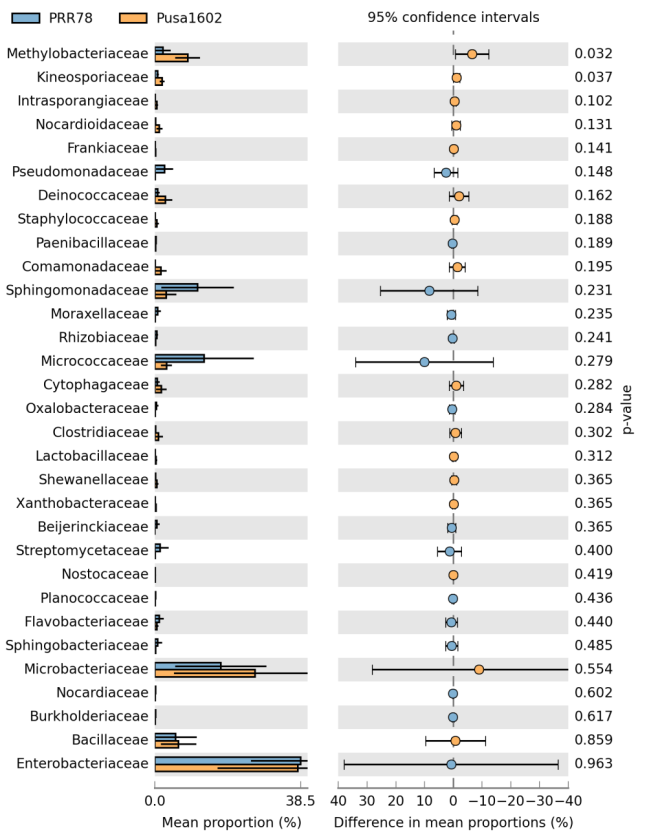** |

**Supplementary Fig. 3. Extended error bar plot at various taxonomic hierarchy of phyllomicrobiome of rice genotypes, PRR78 and Pusa Basmati 1602**

| **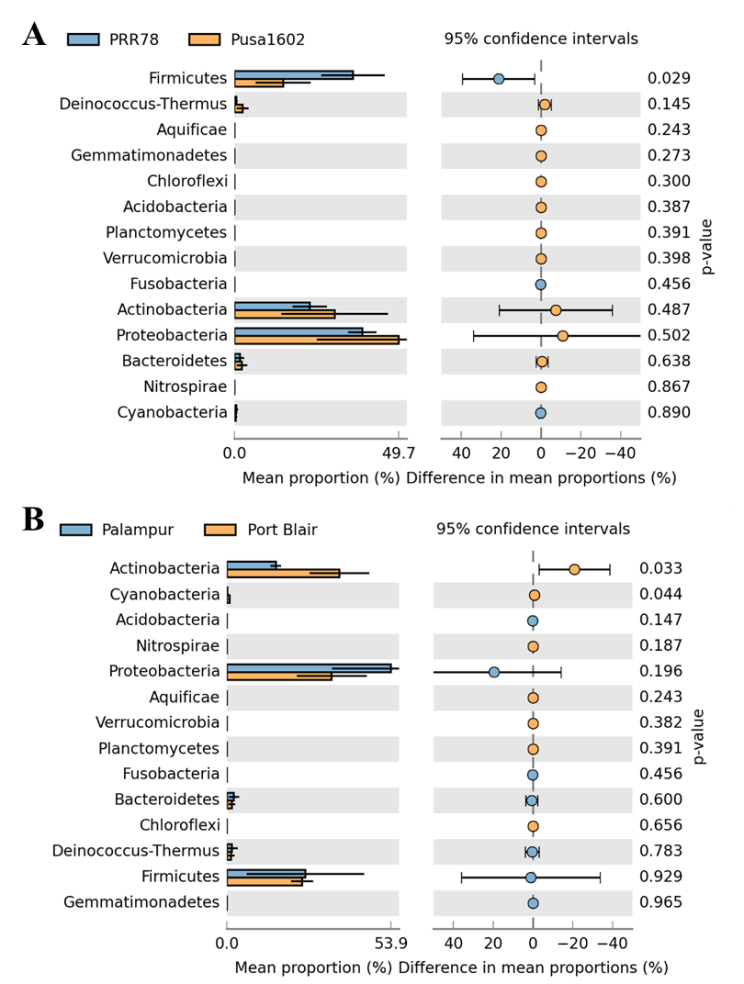** | **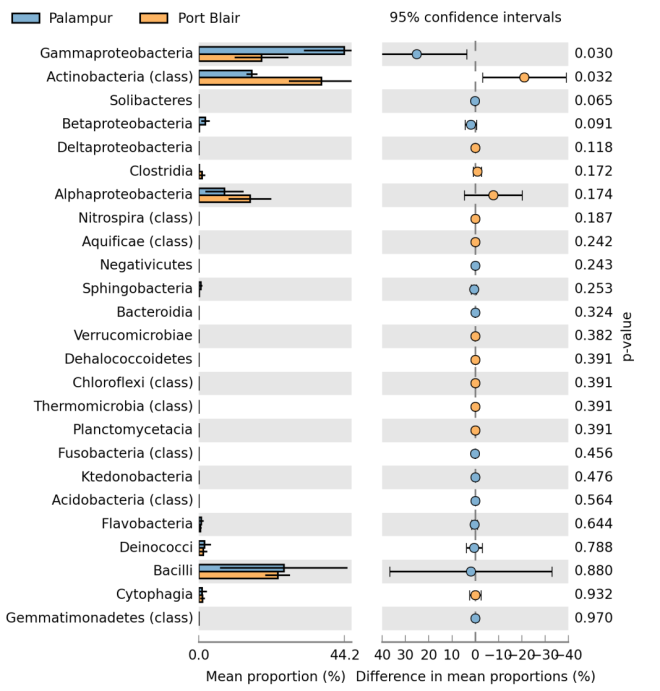** |
| --- | --- |
| **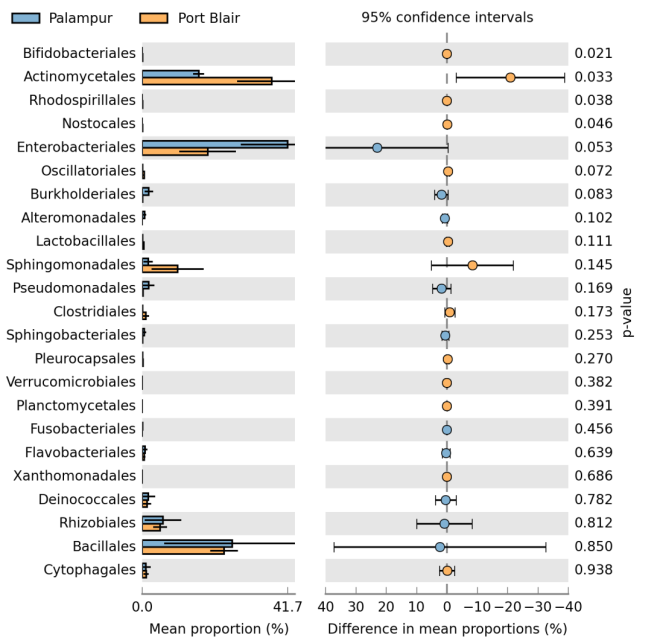** | **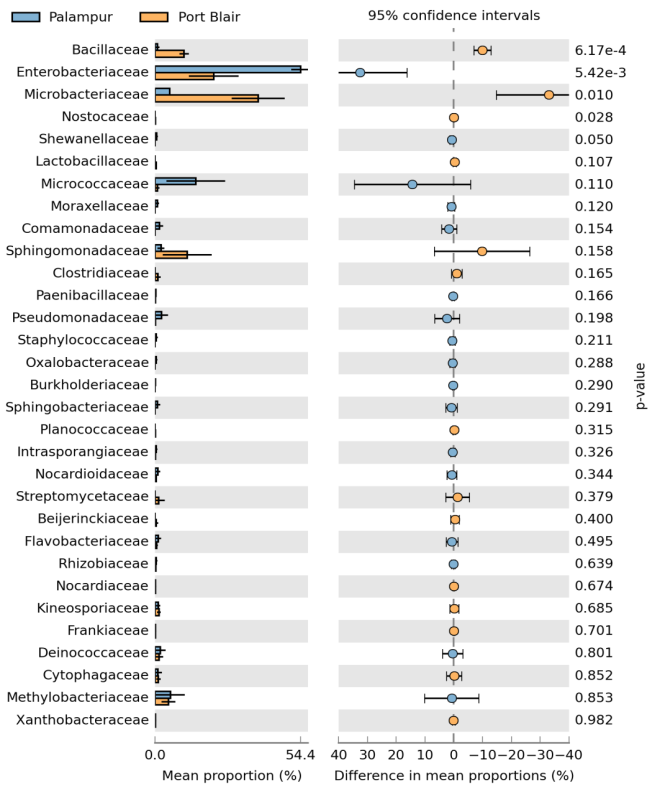** |

**Supplementary Fig. 4. Extended error bar plot at various taxonomic hierarchy level of phyllomicrobiome of rice grown in Palampur, Himachal Pradesh and Port Blair, Andaman Island**

|  |  |
| --- | --- |
|  |  |

**Supplementary Fig. 5. Relative abundance of phyllosphere bacterial communities on rice genotypes grown in two agroclimatic zones of India**

**Supplementary Fig. 6. Relative abundance of phyllosphere bacterial communities at genus level on two rice genotypes representing contrasting agroclimatic zones of India**

| **Marker** | **15P1** | **15P2** | **15P3** | **15P4** | **15P5** | **15P6** | **15P7** | **15P8** | **15P9** | **15P10** | **15P11** | **15P12** | **15P13** | **15P14** | **15P15** | **30P1** | **30P2** | **30P3** | **30P4** | **30P5** | **30P6** | **30P7** | **30P8** | **30P9** | **30P10** | **30P11** | **30P12** | **30P13** | **30P14** | **30P15** | **30P16** | **30P17** | **30P18** | **30P19** | **30P20** | **30P21** | **30P22** | **30P23** | **Marker** |
| --- | --- | --- | --- | --- | --- | --- | --- | --- | --- | --- | --- | --- | --- | --- | --- | --- | --- | --- | --- | --- | --- | --- | --- | --- | --- | --- | --- | --- | --- | --- | --- | --- | --- | --- | --- | --- | --- | --- | --- |

1. **Palampur**

| **Marker** | **15A1** | **115A2** | **15A3** | **15A4** | **15A5** | **15A6** | **15A7** | **15A8** | **15A9** | **15A10** | **15A11** | **15A12** | **15A13** | **15A14** | **15A15** | **15A16** | **15A17** | **15A18** | **30A1** | **30A2** | **30A3** | **30A4** | **30A5** | **30A6** | **30A7** | **30A8** | **30A9** | **30A10** | **30A11** | **30A12** | **30A13** | **30A14** | **30A15** | **30A6** | **30A17** | **30A18** | **30A19** | **30A20** | **30A21** | **30A22** | **Marker** |
| --- | --- | --- | --- | --- | --- | --- | --- | --- | --- | --- | --- | --- | --- | --- | --- | --- | --- | --- | --- | --- | --- | --- | --- | --- | --- | --- | --- | --- | --- | --- | --- | --- | --- | --- | --- | --- | --- | --- | --- | --- | --- |

1. **Andaman & Nicobar Island**

**Supplementary Fig 7. BOX PCR finger printing of cultured bacterial isolates of rice phyllosphere; M: DNA size marker; Lanes: Isolates of bacteria isolated from phyllosphere of rice leaf;**

**Note:** Isolates **15P4, 15P8, 15P9, 15P10, 15P13 and 15P15** shared all amplicons; one of them, 15P9, was considered as representative isolate for further work; **Note:** Isolates **30P3 and 30P5** shared all amplicons; one of them, 30P3, was considered as representative isolate for further work; **Note:** Isolates **30P9 and 30P13** shared all amplicons; one of them, 30P9, was considered as representative isolate for further work; **Note:** Isolates **30P4, 30P8, 30P12 and 30P15** shared all amplicons; one of them, 30P4, was considered as representative isolate for further work

**Note:** Isolates, **15A7 and 15A16** shared all amplicons; one of them, **15A7**, was considered as representative isolate for further work; **Note:** Isolates **15A10, 15A11, 15A17 and 15A18** shared all amplicons; one of them, **15A10**, was considered as representative isolate for further work; **Note:** Isolates **15A12, 15A13 and 15A14** shared all amplicons; one of them, **15A12**, was considered as representative isolate for further work; **Note:** Isolates **30A5, 30A7 and 30A12** shared all amplicons; one of them, **30A5**, was considered as representative isolate for further work; **Note:** Isolates **30A6 and 30A16** shared all amplicons; one of them, **30A6**, was considered as representative isolate for further work

| **Marker** | **15P1** | **15P2** | **15P3** | **15P5** | **15P6** | **15P7** | **15P9** | **15P11** | **15P12** | **15P14** | **30P1** | **30P2** | **30P3** | **30P4** | **30P6** | **30P7** | **30P9** | **30P10** | **30P11** | **30P14** | **30P16** | **30P17** | **30P18** | **30P19** | **30P20** | **30P21** | **30P22** | **30P23** | **Marker** |
| --- | --- | --- | --- | --- | --- | --- | --- | --- | --- | --- | --- | --- | --- | --- | --- | --- | --- | --- | --- | --- | --- | --- | --- | --- | --- | --- | --- | --- | --- |

**B. Palampur**

| **Marker** | **15A1** | **115A2** | **15A3** | **15A4** | **15A5** | **15A6** | **15A7** | **15A8** | **15A9** | **15A10** | **15A12** | **15A15** | **30A1** | **30A2** | **30A3** | **30A4** | **30A5** | **30A6** | **30A8** | **30A9** | **30A10** | **30A11** | **30A13** | **30A14** | **30A15** | **30A17** | **30A18** | **30A19** | **30A20** | **30A21** | **30A22** | **Marker** |
| --- | --- | --- | --- | --- | --- | --- | --- | --- | --- | --- | --- | --- | --- | --- | --- | --- | --- | --- | --- | --- | --- | --- | --- | --- | --- | --- | --- | --- | --- | --- | --- | --- |

**C. Andaman & Nicobar Island**

**Supplementary Fig. 8. Amplification of 16S rRNA gene of bacterial isolates of rice phyllosphere**

**M: DNA size marker; Lane 1-31: Phyllosphere bacteria isolated from phyllosphere of rice leaf**

| **Colonies of Rice epiphytic *Acidovorax*** | | **Close up view of *Acidovorax* colonies** | | **Species identity**  **& Isolate name** |
| --- | --- | --- | --- | --- |
| **Nutrient Agar** | **Nutrient agar +**  **2, 3, 5 triphenyl tetrazolium chloride** | **Nutrient Agar** | **Nutrient agar +**  **2, 3, 5 triphenyl tetrazolium chloride** |  |
| 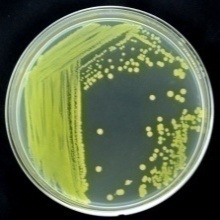 | 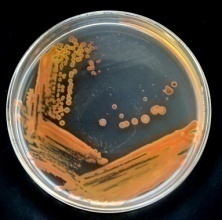 | 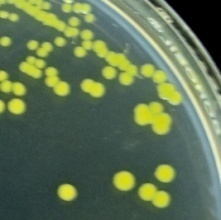 | 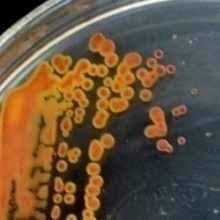 | *Acidovorax avenae*  (OsEp-Plm-30P1) |
| 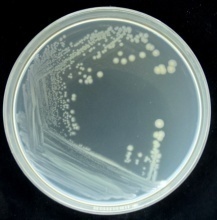 | 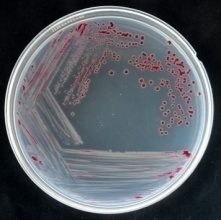 | 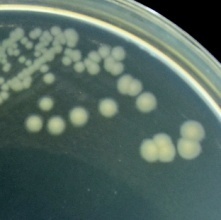 | 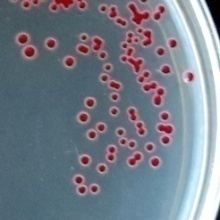 | *Acidovorax avenae*  (OsEp-Plm-30P23) |
| 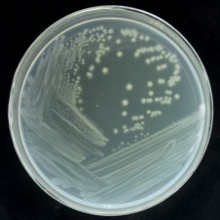 | 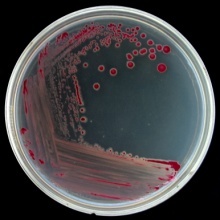 | 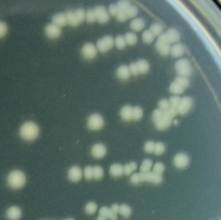 | 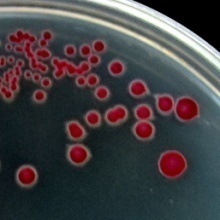 | *Acidovorax avenae*  (OsEp-Plm-30P6) |

**Supplementary Fig. 9a. Colonies of cultured *Acidovorax* species from rice phyllomicrobiome**

| **Colonies of Rice epiphytic *Acinetobacter*** | | **Close up view of *Acinetobacter* colonies** | | **Species identity**  **& Isolate name** |
| --- | --- | --- | --- | --- |
| **Nutrient Agar** | **Nutrient agar +**  **2, 3, 5 triphenyl tetrazolium chloride** | **Nutrient Agar** | **Nutrient agar +**  **2, 3, 5 triphenyl tetrazolium chloride** |  |
| 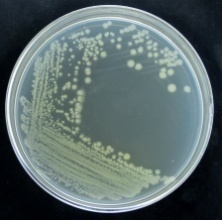 | 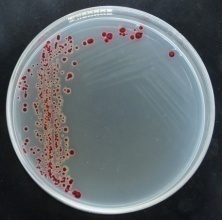 | 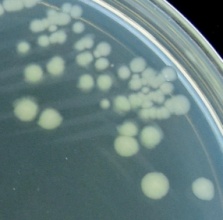 | 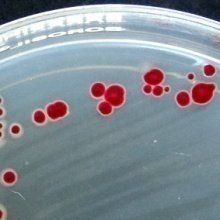 | *Acinetobacter baumannii* (OsEp-Plm-30P11) |
| 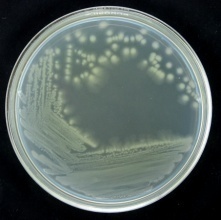 | 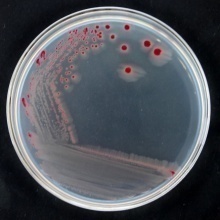 | 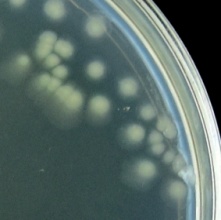 | 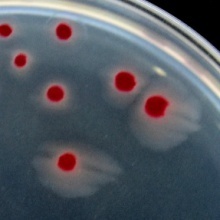 | *Acinetobacter baumannii* (OsEp-Plm-30P17) |
| 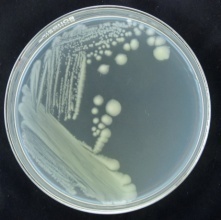 | 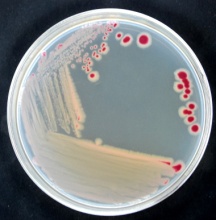 | 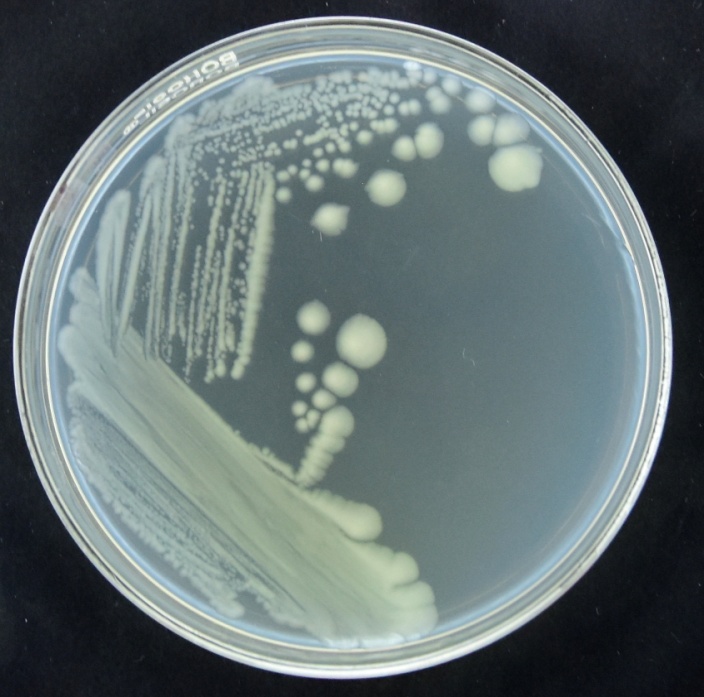 | 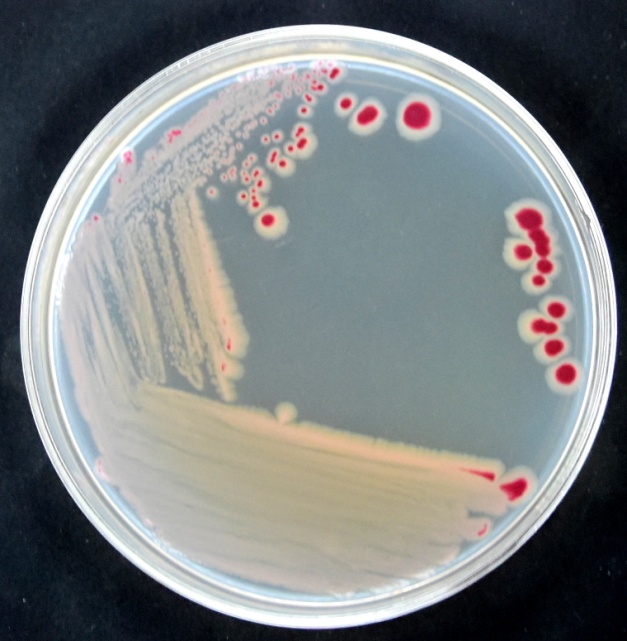 | *Acinetobacter junii* (OsEp-AN-30A17) |
| 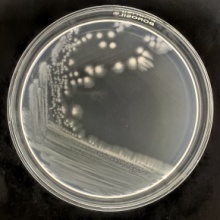 | 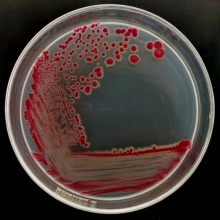 | 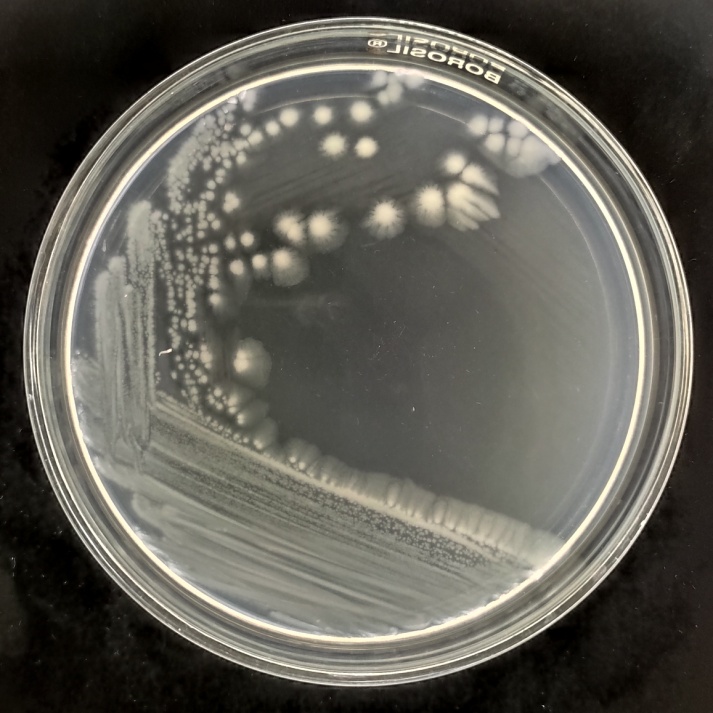 | 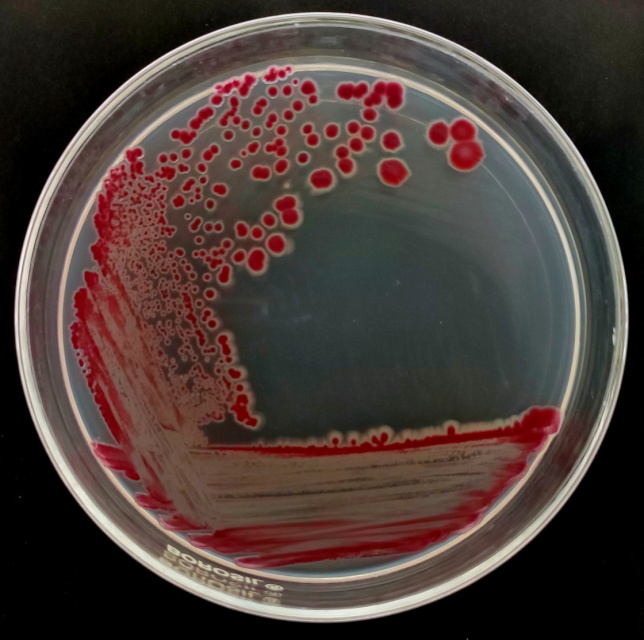 | *Acinetobacter soli* (OsEp-Plm-30P2) |
| 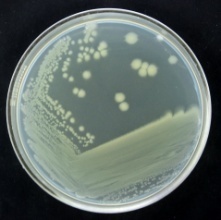 | 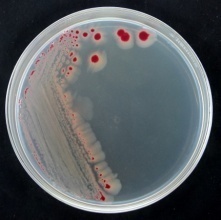 | 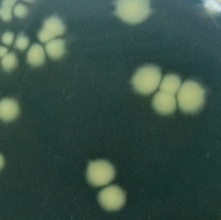 | 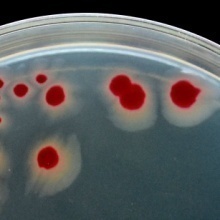 | *Acinetobacter soli* (OsEp-Plm-30P22) |
| 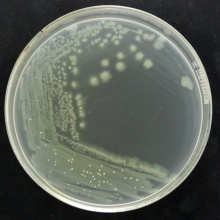 | 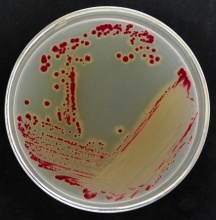 | 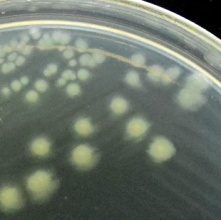 | 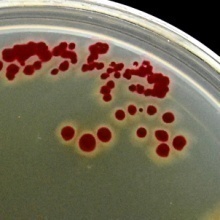 | *Acinetobacter soli* (OsEp-Plm-30P4) |

**Supplementary Fig. 9b. Colonies of cultured *Acinetobacter* species from rice phyllomicrobiome**

| **Colonies of Rice epiphytic** *Agrobacterium* | | **Close up view of** *Agrobacterium* **colonies** | | **Species identity**  **& Isolate name** |
| --- | --- | --- | --- | --- |
| **Nutrient Agar** | **Nutrient agar +**  **2, 3, 5 triphenyl tetrazolium chloride** | **Nutrient Agar** | **Nutrient agar +**  **2, 3, 5 triphenyl tetrazolium chloride** |  |
| 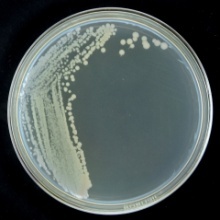 | 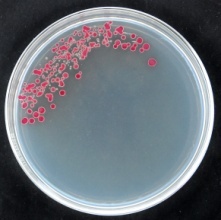 | 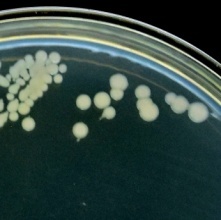 | 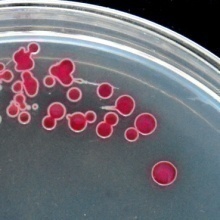 | *Agrobacterium larrymoorei* (OsEp-Plm-30P19) |

**Supplementary Fig. 9c. Colonies of cultured *Agrobacterium* species from rice phyllomicrobiome**

| **Colonies of Rice epiphytic *Aureimonas*** | | **Close up view of *Aureimonas* colonies** | | **Species identity**  **& Isolate name** |
| --- | --- | --- | --- | --- |
| **Nutrient Agar** | **Nutrient agar +**  **2, 3, 5 triphenyl tetrazolium chloride** | **Nutrient Agar** | **Nutrient agar +**  **2, 3, 5 triphenyl tetrazolium chloride** |  |
| 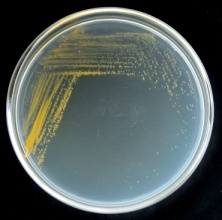 | 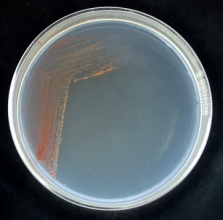 | 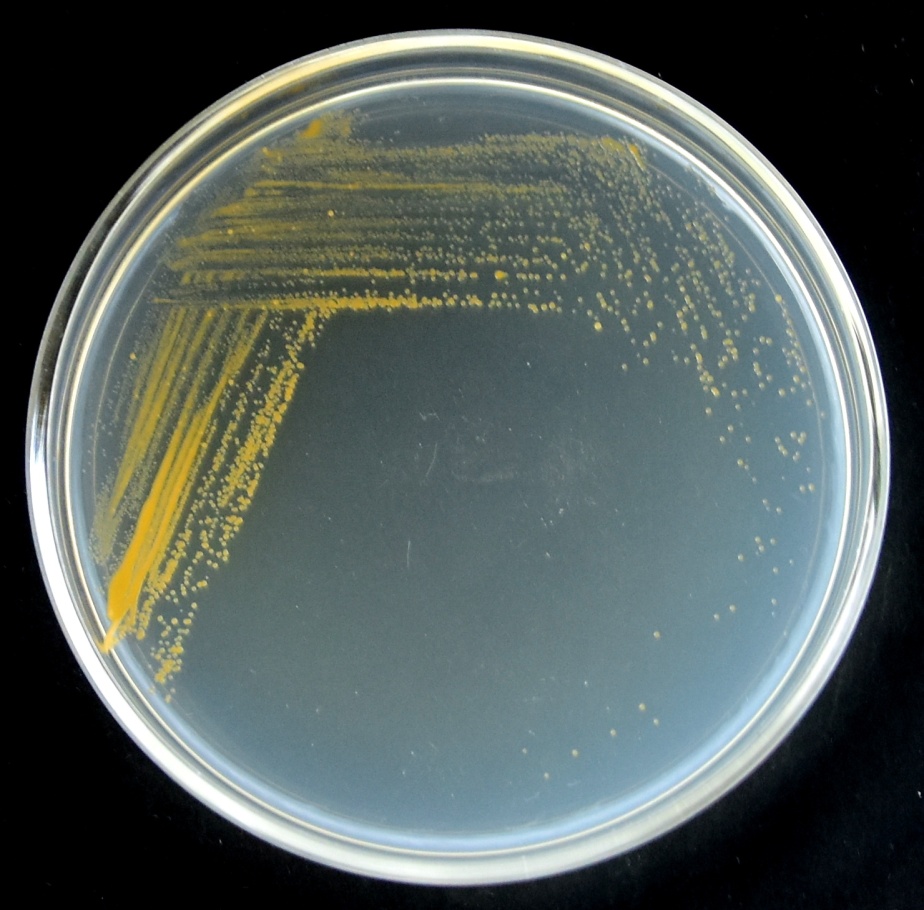 | 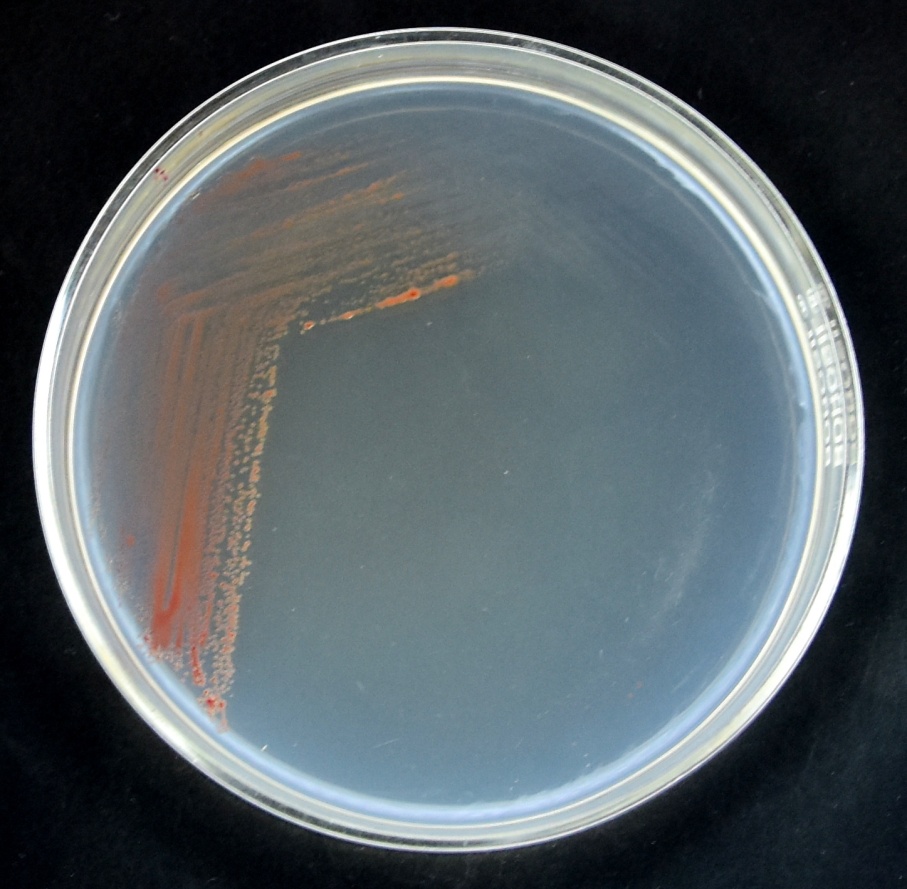 | *Aureimonas phyllosphaerae* (OsEp-AN-30A11) |
| 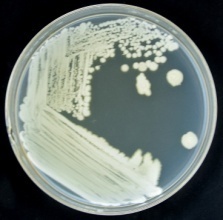 | 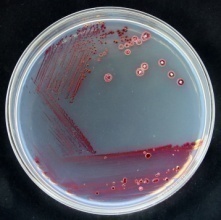 | 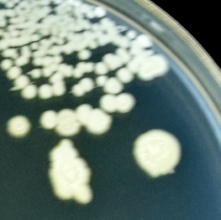 | 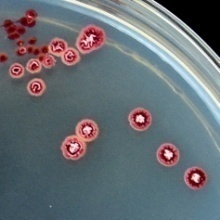 | *Aureimonas* sp. (OsEp-Plm-30P7) |

**Supplementary Fig. 9d. Colonies of cultured *Aureimonas* species from rice phyllomicrobiome**

| **Colonies of Rice epiphytic *Curtobacterium*** | | **Close up view of *Curtobacterium* colonies** | | **Species identity**  **& Isolate name** |
| --- | --- | --- | --- | --- |
| **Nutrient Agar** | **Nutrient agar +**  **2, 3, 5 triphenyl tetrazolium chloride** | **Nutrient Agar** | **Nutrient agar +**  **2, 3, 5 triphenyl tetrazolium chloride** |  |
| **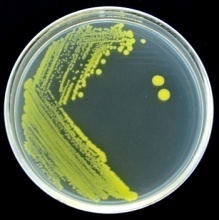** | **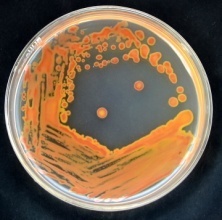** | **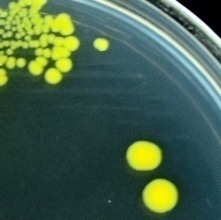** | 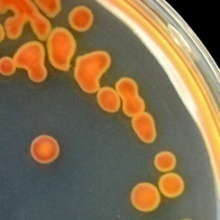 | *Curtobacterium albidum* (OsEp-Plm-15P1) |
| 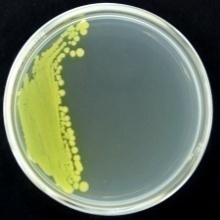 | 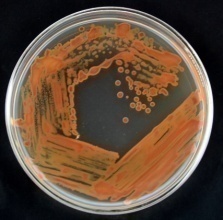 | 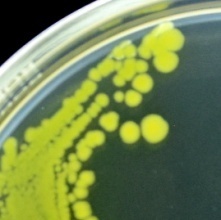 | 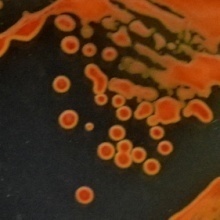 | *Curtobacterium albidum* (OsEp-Plm-30P20) |
| 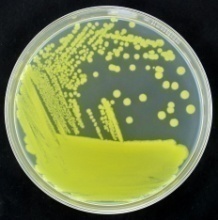 | 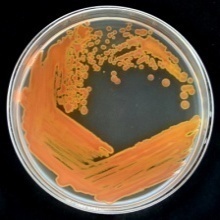 | 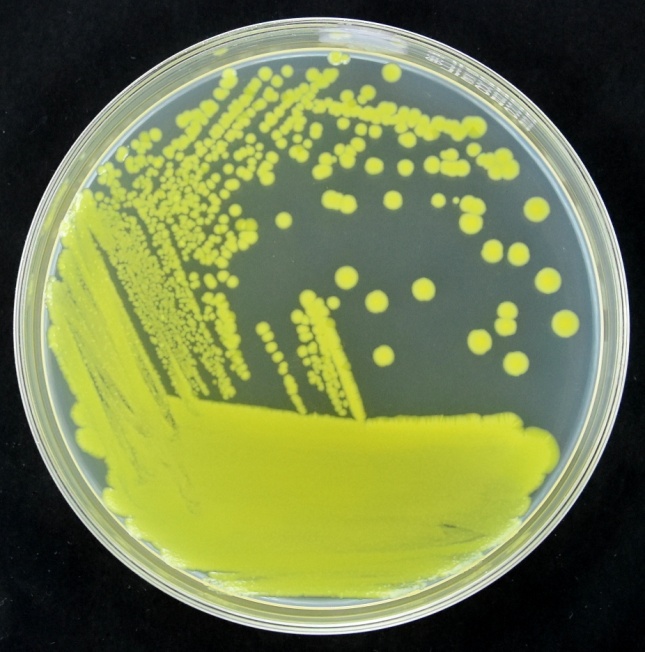 | 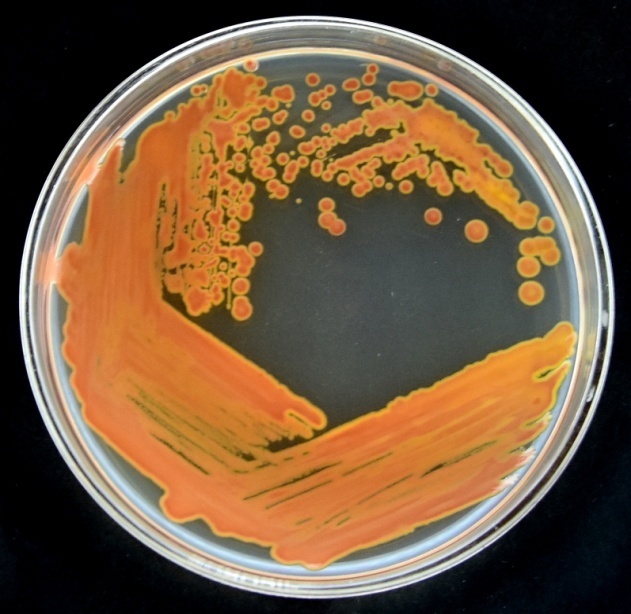 | *Curtobacterium citreum* (OsEp-AN-30A1) |
| **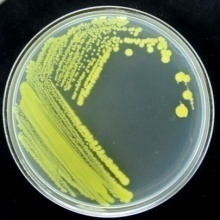** | **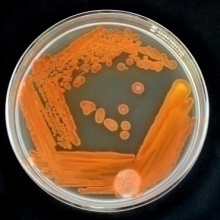** | **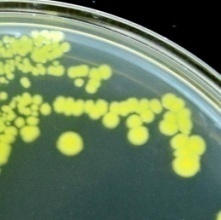** | 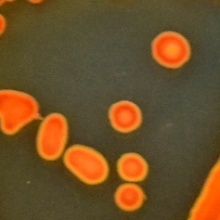 | *Curtobacterium luteum* (OsEp-Plm-15P7) |
| 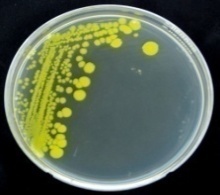 | 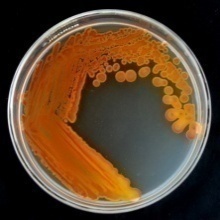 | 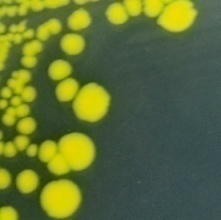 | 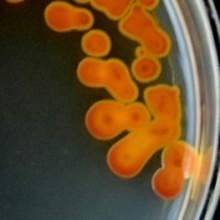 | *Curtobacterium luteum* (OsEp-Plm-30P9) |

**Supplementary Fig. 9e. Colonies of cultured *Curtobacterium* species from rice phyllomicrobiome**

| **Colonies of Rice epiphytic** *Enterobacter* | | **Close up view of** *Enterobacter* **colonies** | | **Species identity**  **& Isolate name** |
| --- | --- | --- | --- | --- |
| **Nutrient Agar** | **Nutrient agar +**  **2, 3, 5 triphenyl tetrazolium chloride** | **Nutrient Agar** | **Nutrient agar +**  **2, 3, 5 triphenyl tetrazolium chloride** |  |
| 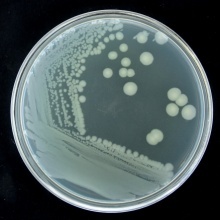 | 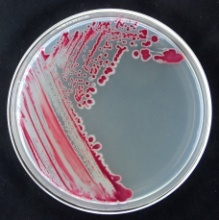 | 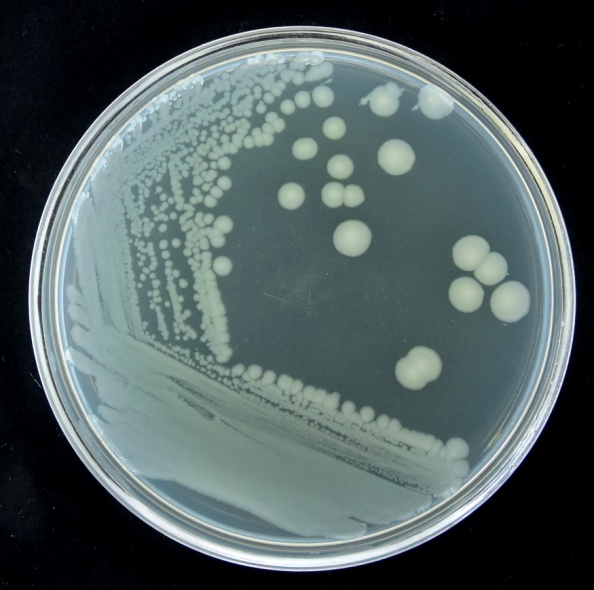 | 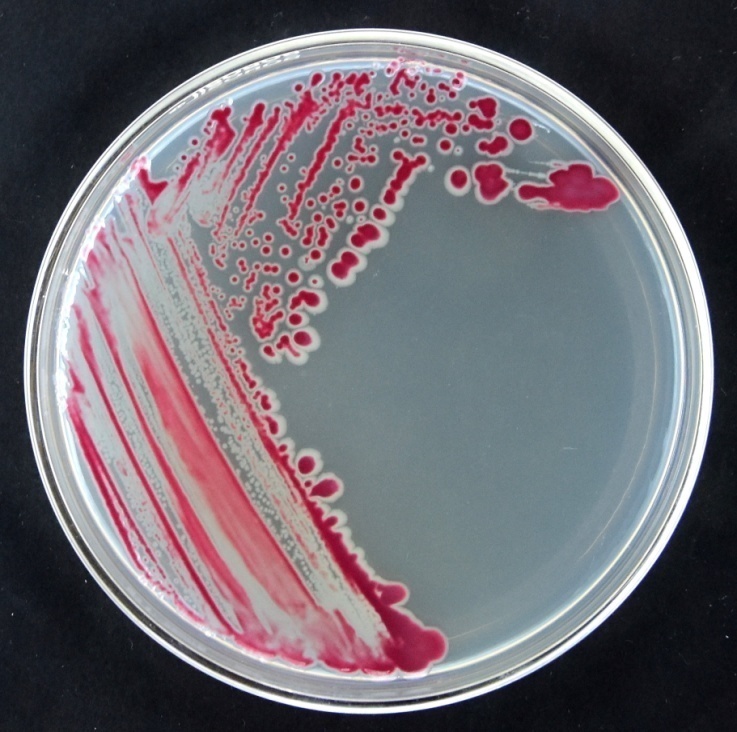 | *Enterobacter asburiae* (OsEp-AN-30A22) |
| 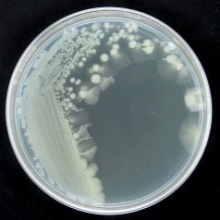 | 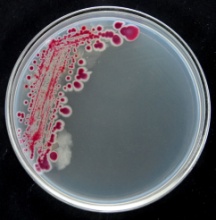 | 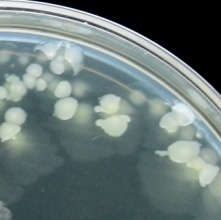 | 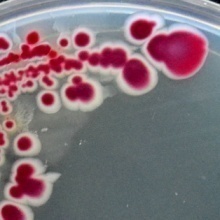 | *Enterobacter asburiae* (OsEp-Plm-30P16) |
| **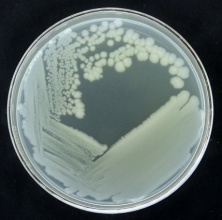** | **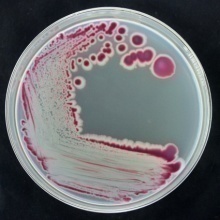** | **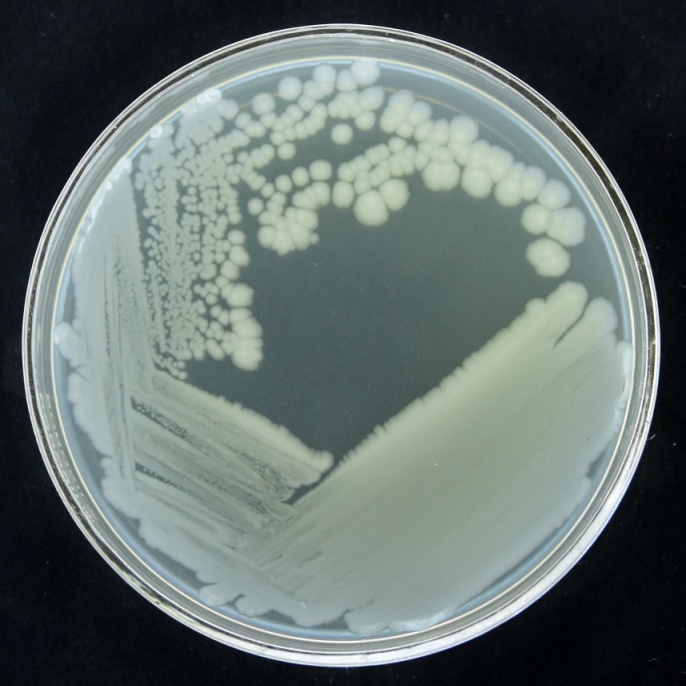** | **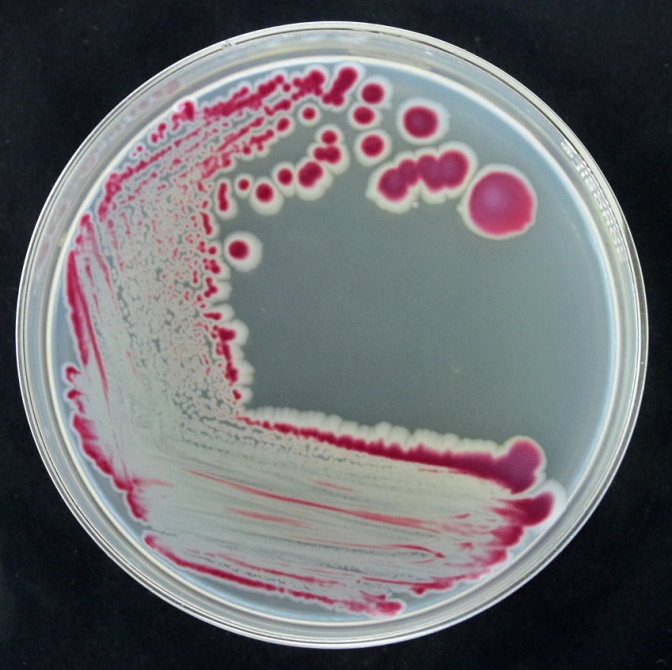** | *Enterobacter cloacae* (OsEp-AN-15A7) |
| 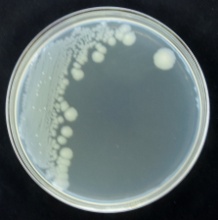 | 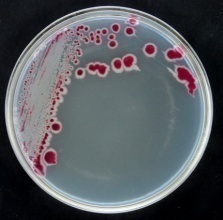 | 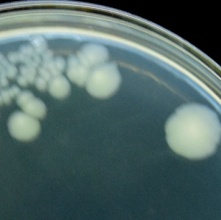 | 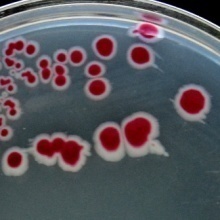 | *Enterobacter cloacae* (OsEp-Plm-30P18) |
| 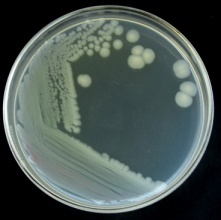 | 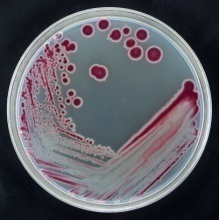 | 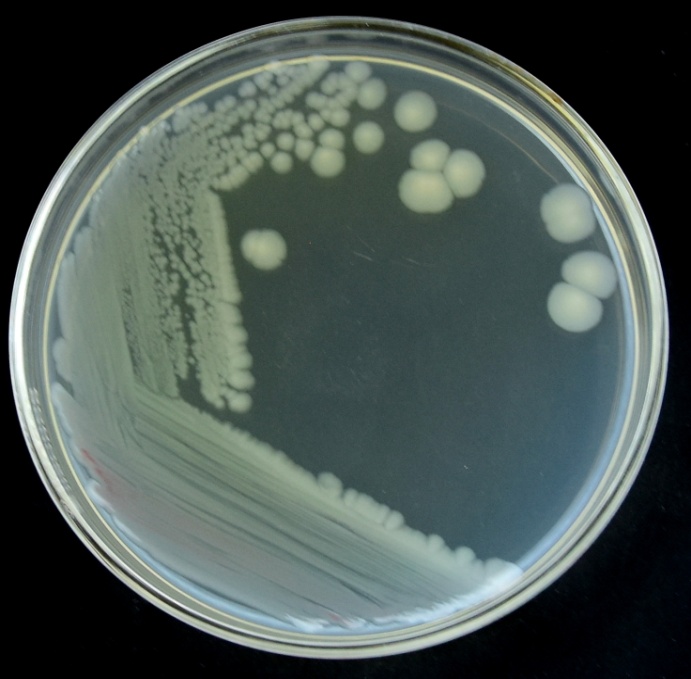 | 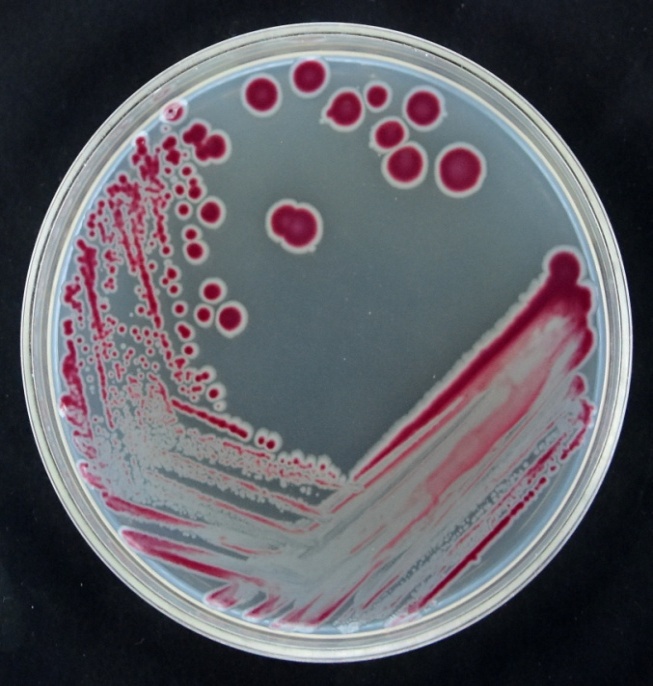 | *Enterobacter mori* (OsEp-AN-30A20) |
| 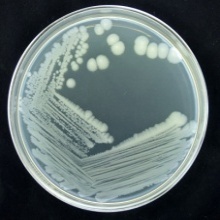 |  |  |  | *Enterobacter sichuanensis* (OsEp-AN-15A12) |

**Supplementary Fig. 9f. Colonies of cultured *Enterobacter* species from rice phyllomicrobiome**

| **Colonies of Rice epiphytic** *Erwinia* | | **Close up view of** *Erwinia* **colonies** | | **Species identity**  **& Isolate name** |
| --- | --- | --- | --- | --- |
| **Nutrient Agar** | **Nutrient agar +**  **2, 3, 5 triphenyl tetrazolium chloride** | **Nutrient Agar** | **Nutrient agar +**  **2, 3, 5 triphenyl tetrazolium chloride** |  |
|  |  |  |  | *Erwinia tasmaniensis* (OsEp-AN-15A5) |

**Supplementary Fig. 9g. Colonies of cultured *Erwinia* species from rice phyllomicrobiome**

| **Colonies of Rice epiphytic** *Exiguobacterium* | | **Close up view of** *Exiguobacterium* **colonies** | | **Species identity**  **& Isolate name** |
| --- | --- | --- | --- | --- |
| **Nutrient Agar** | **Nutrient agar +**  **2, 3, 5 triphenyl tetrazolium chloride** | **Nutrient Agar** | **Nutrient agar +**  **2, 3, 5 triphenyl tetrazolium chloride** |  |
|  |  |  |  | *Exiguobacterium acetylicum* (OsEp-Plm-15P3) |
|  |  |  |  | *Exiguobacterium indicum* (OsEp-AN-30A4) |
|  |  |  |  | *Exiguobacterium indicum* (OsEp-AN-30A6) |
|  |  |  |  | *Exiguobacterium indicum* (OsEp-Plm-30P14) |

**Supplementary Fig. 9h. Colonies of cultured *Exiguobacterium* species from rice phyllomicrobiome**

| **Colonies of Rice epiphytic *Microbacterium*** | | **Close up view of *Microbacterium* colonies** | | **Species identity**  **& Isolate name** |
| --- | --- | --- | --- | --- |
| **Nutrient Agar** | **Nutrient agar +**  **2, 3, 5 triphenyl tetrazolium chloride** | **Nutrient Agar** | **Nutrient agar +**  **2, 3, 5 triphenyl tetrazolium chloride** |  |
|  |  |  |  | *Microbacterium* sp.  (OsEp-AN-15A2) |
|  |  |  |  | *Microbacterium testaceum* (OsEp-AN-30A2) |

**Supplementary Fig. 9i. Colonies of cultured** *Microbacterium* **species from rice phyllomicrobiome**

| **Colonies of Rice epiphytic *Micrococcus*** | | **Close up view of *Micrococcus* colonies** | | **Species identity**  **& Isolate name** |
| --- | --- | --- | --- | --- |
| **Nutrient Agar** | **Nutrient agar +**  **2, 3, 5 triphenyl tetrazolium chloride** | **Nutrient Agar** | **Nutrient agar +**  **2, 3, 5 triphenyl tetrazolium chloride** |  |
|  |  |  |  | *Micrococcus luteus* (OsEp-AN-15A1) |

**Supplementary Fig. 9j. Colonies of cultured** *Micrococcus* **species from rice phyllomicrobiome**

| **Colonies of Rice epiphytic** *Pantoea* | | **Close up view of** *Pantoea* **colonies** | | **Species identity**  **& Isolate name** |
| --- | --- | --- | --- | --- |
| **Nutrient Agar** | **Nutrient agar +**  **2, 3, 5 triphenyl tetrazolium chloride** | **Nutrient Agar** | **Nutrient agar +**  **2, 3, 5 triphenyl tetrazolium chloride** |  |
|  |  |  |  | *Pantoea agglomerans* (OsEp-AN-15A8) |
|  |  |  |  | *Pantoea agglomerans* (OsEp-AN-30A14) |
|  |  |  |  | *Pantoea agglomerans* (OsEp-AN-30A21) |
|  |  |  |  | *Pantoea ananatis* (OsEp-AN-15A10) |
|  |  |  |  | *Pantoea ananatis* (OsEp-AN-30A19) |
|  |  |  |  | *Pantoea ananatis* (OsEp-AN-30A5) |
|  |  |  |  | *Pantoea ananatis* (OsEp-AN-30A8) |
|  |  |  |  | *Pantoea ananatis* (OsEp-Plm-15P9) |
|  |  |  |  | *Pantoea ananatis* (OsEp-Plm-30P21) |
|  |  |  |  | *Pantoea ananatis* (OsEp-Plm-30P3) |
|  |  |  |  | *Pantoea dispersa* (OsEp-AN-30A18) |
|  |  |  |  | *Pantoea eucrina* (OsEp-AN-15A4) |
|  |  |  |  | *Pantoea eucrina* (OsEp-Plm-15P14) |
|  |  |  |  | *Pantoea eucrina* (OsEp-Plm-30P10) |
|  |  |  |  | *Pantoea sp.* (OsEp-AN-15A15) |
|  |  |  |  | *Pantoea sp.* (OsEp-AN-15A9) |

**Supplementary Fig. 9k. Colonies of cultured *Pantoea* species from rice phyllomicrobiome**

| **Colonies of Rice epiphytic** *Pseudomonas* | | **Close up view of** *Pseudomonas* **colonies** | | **Species identity**  **& Isolate name** |
| --- | --- | --- | --- | --- |
| **Nutrient Agar** | **Nutrient agar +**  **2, 3, 5 triphenyl tetrazolium chloride** | **Nutrient Agar** | **Nutrient agar +**  **2, 3, 5 triphenyl tetrazolium chloride** |  |
|  |  |  |  | *Pseudomonas oryzihabitans* (OsEp-Plm-15P6) |
|  |  |  |  | *Pseudomonas parafulva* (OsEp-Plm-15P12) |
|  |  |  |  | *Pseudomonas psychrotolerans* (OsEp-AN-15A6) |
|  |  |  |  | *Pseudomonas psychrotolerans* (OsEp-AN-30A13) |
|  |  |  |  | *Pseudomonas putida* (OsEp-Plm-15P11) |

**Supplementary Fig. 9l. Colonies of cultured** *Pseudomonas* **species from rice phyllomicrobiome**

| **Colonies of Rice epiphytic** *Sphingomonas* | | **Close up view of** *Sphingomonas* **colonies** | | **Species identity**  **& Isolate name** |
| --- | --- | --- | --- | --- |
| **Nutrient Agar** | **Nutrient agar +**  **2, 3, 5 triphenyl tetrazolium chloride** | **Nutrient Agar** | **Nutrient agar +**  **2, 3, 5 triphenyl tetrazolium chloride** |  |
|  |  |  |  | *Sphingomonas paucimobilis* (OsEp-AN-15A3) |
|  |  |  |  | *Sphingomonas paucimobilis* (OsEp-AN-30A9) |
|  |  |  |  | *Sphingomonas pseudosanguinis* (OsEp-AN-30A10) |
|  |  |  |  | *Sphingomonas pseudosanguinis* (OsEp-Plm-15P2) |
|  |  |  |  | *Sphingomonas sp.* (OsEp-AN-30A15) |
|  |  |  |  | *Sphingomonas sp.* (OsEp-Plm-15P5) |
|  |  |  |  | *Sphingomonas yabuuchiae* (OsEp-AN-30A3) |

**Supplementary Fig. 9m. Colonies of cultured *Sphingomonas* species from rice phyllomicrobiome**

| ***Acidovorax*** | ***Acidovorax avenae***  **OsEp-Plm-30P1** | ***Acidovorax avenae***  **OsEp-Plm-30P23** | ***Acidovorax avenae***  **OsEp-Plm-30P6** |
| --- | --- | --- | --- |
|  |  |  |  |
| ***Acinetobacter*** | ***Acinetobacter baumannii***  **OsEp-Plm-30P11** | ***Acinetobacter baumannii***  **OsEp-Plm-30P17** | ***Acinetobacter junii***  **OsEp-AN-30A17** |
|  |  |  |  |
|  | ***Acinetobacter soli***  **OsEp-Plm-30P2** | ***Acinetobacter soli***  **OsEp-Plm-30P22** | ***Acinetobacter soli***  **OsEp-Plm-30P4** |
|  |  |  |  |
| ***Agrobacterium*** | ***Agrobacterium larrymoorei***  **OsEp-Plm-30P19** |  |  |
|  |  |  |  |
| ***Aureimonas*** | ***Aureimonas phyllosphaerae***  **OsEp-AN-30A11** | ***Aureimonas* sp.**  **OsEp-Plm-30P7** |  |
|  |  |  |  |
| ***Curtobacterium*** | ***Curtobacterium albidum* OsEp-Plm-15P1** | ***Curtobacterium albidum* OsEp-Plm-30P20** | ***Curtobacterium citreum***  **OsEp-AN-30A1** |
|  |  |  |  |
|  | ***Curtobacterium luteum***  **OsEp-Plm-15P7** | ***Curtobacterium luteum***  **OsEp-Plm-30P9** |  |
|  |  |  |  |
| ***Enterobacter*** | ***Enterobacter asburiae***  **OsEp-AN-30A22** | ***Enterobacter asburiae***  **OsEp-Plm-30P16** | ***Enterobacter cloacae***  **OsEp-AN-15A7** |
|  |  |  |  |
|  | ***Enterobacter cloacae***  **OsEp-Plm-30P18** | ***Enterobacter mori***  **OsEp-AN-30A20** | ***Enterobacter sichuanensis***  **OsEp-AN-15A12** |
|  |  |  |  |
| ***Erwinia*** | ***Erwinia tasmaniensis***  **OsEp-AN-15A5** |  |  |
|  |  |  |  |
| ***Exiguobacterium*** | ***Exiguobacterium acetylicum***  **OsEp-Plm-15P3** | ***Exiguobacterium indicum***  **OsEp-AN-30A4** | ***Exiguobacterium indicum***  **OsEp-AN-30A6** |
|  |  |  |  |
|  | ***Exiguobacterium indicum***  **OsEp-Plm-30P14** |  |  |
|  |  |  |  |
| ***Microbacterium*** | ***Microbacterium sp.***  **OsEp-AN-15A2** | ***Microbacterium testaceum***  **OsEp-AN-30A2** |  |
|  |  |  |  |
| ***Micrococcus*** | ***Micrococcus luteus***  **OsEp-AN-15A1** |  |  |
|  |  |  |  |
| ***Pantoea*** | ***Pantoea agglomerans***  **OsEp-AN-15A8** | ***Pantoea agglomerans***  **OsEp-AN-30A14** | ***Pantoea agglomerans***  **OsEp-AN-30A21** |
|  |  |  |  |
|  | ***Pantoea ananatis***  **OsEp-AN-15A10** | ***Pantoea ananatis***  **OsEp-AN-30A19** | ***Pantoea ananatis***  **OsEp-AN-30A5** |
|  |  |  |  |
|  | ***Pantoea ananatis***  **OsEp-AN-30A8** | ***Pantoea ananatis***  **OsEp-Plm-15P9** | ***Pantoea ananatis***  **OsEp-Plm-30P21** |
|  |  |  |  |
|  | ***Pantoea ananatis***  **OsEp-Plm-30P3** | ***Pantoea dispersa***  **OsEp-AN-30A18** | ***Pantoea eucrina***  **OsEp-AN-15A4** |
|  |  |  |  |
|  | ***Pantoea eucrina***  **OsEp-Plm-15P14** | ***Pantoea eucrina***  **OsEp-Plm-30P10** | ***Pantoea sp.***  **OsEp-AN-15A15** |
|  |  |  |  |
|  | ***Pantoea sp.***  **OsEp-AN-15A9** |  |  |
|  |  |  |  |
| ***Pseudomonas*** | ***Pseudomonas oryzihabitans***  **OsEp-Plm-15P6** | ***Pseudomonas parafulva***  **OsEp-Plm-15P12** | ***Pseudomonas psychrotolerans***  **OsEp-AN-15A6** |
|  |  |  |  |
|  | ***Pseudomonas psychrotolerans***  **OsEp-AN-30A13** | ***Pseudomonas putida***  **OsEp-Plm-15P11** |  |
|  |  |  |  |
| ***Sphingomonas*** | ***Sphingomonas paucimobilis***  **OsEp-AN-15A3** | ***Sphingomonas paucimobilis***  **OsEp-AN-30A9** | ***Sphingomonas pseudosanguinis***  **OsEp-AN-30A10** |
|  |  |  |  |
|  | ***Sphingomonas pseudosanguinis***  **OsEp-Plm-15P2** | ***Sphingomonas sp.***  **OsEp-AN-30A15** | ***Sphingomonas sp.***  **OsEp-Plm-15P5** |
|  |  |  |  |
|  | ***Sphingomonas yabuuchiae***  **OsEp-AN-30A3** |  |  |
|  |  |  |  |
| ***Mock*** | **Mock** | **Mock** |  |
|  |  |  |  |

**Supplementary Fig. 10. Secreted metabolite mediated *in vitro* antifungal activity of rice phyllosphere bacterial isolates on *Magnaporthe oryzae***

| ***Acidovorax*** | ***Acidovorax avenae***  **OsEp-Plm-30P1** | ***Acidovorax avenae***  **OsEp-Plm-30P23** | ***Acidovorax avenae***  **OsEp-Plm-30P6** |
| --- | --- | --- | --- |
|  |  |  |  |
| ***Acinetobacter*** | ***Acinetobacter baumannii***  **OsEp-Plm-30P11** | ***Acinetobacter baumannii***  **OsEp-Plm-30P17** | ***Acinetobacter junii***  **OsEp-AN-30A17** |
|  |  |  |  |
|  | ***Acinetobacter soli***  **OsEp-Plm-30P2** | ***Acinetobacter soli***  **OsEp-Plm-30P22** | ***Acinetobacter soli***  **OsEp-Plm-30P4** |
|  |  |  |  |
| ***Agrobacterium*** | ***Agrobacterium larrymoorei***  **OsEp-Plm-30P19** |  |  |
|  |  |  |  |
| ***Aureimonas*** | ***Aureimonas phyllosphaerae***  **OsEp-AN-30A11** | ***Aureimonas* sp.**  **OsEp-Plm-30P7** |  |
|  |  |  |  |
| ***Curtobacterium*** | ***Curtobacterium albidum* OsEp-Plm-15P1** | ***Curtobacterium albidum* OsEp-Plm-30P20** | ***Curtobacterium citreum***  **OsEp-AN-30A1** |
|  |  |  |  |
|  | ***Curtobacterium luteum***  **OsEp-Plm-15P7** | ***Curtobacterium luteum***  **OsEp-Plm-30P9** |  |
|  |  |  |  |
| ***Enterobacter*** | ***Enterobacter asburiae***  **OsEp-AN-30A22** | ***Enterobacter asburiae***  **OsEp-Plm-30P16** | ***Enterobacter cloacae***  **OsEp-AN-15A7** |
|  |  |  |  |
|  | ***Enterobacter cloacae***  **OsEp-Plm-30P18** | ***Enterobacter mori***  **OsEp-AN-30A20** | ***Enterobacter sichuanensis***  **OsEp-AN-15A12** |
|  |  |  |  |
| ***Erwinia*** | ***Erwinia tasmaniensis***  **OsEp-AN-15A5** |  |  |
|  |  |  |  |
| ***Exiguobacterium*** | ***Exiguobacterium acetylicum***  **OsEp-Plm-15P3** | ***Exiguobacterium indicum***  **OsEp-AN-30A4** | ***Exiguobacterium indicum***  **OsEp-AN-30A6** |
|  |  |  |  |
|  | ***Exiguobacterium indicum***  **OsEp-Plm-30P14** |  |  |
|  |  |  |  |
| ***Microbacterium*** | ***Microbacterium sp.***  **OsEp-AN-15A2** | ***Microbacterium testaceum***  **OsEp-AN-30A2** |  |
|  |  |  |  |
| ***Micrococcus*** | ***Micrococcus luteus***  **OsEp-AN-15A1** |  |  |
|  |  |  |  |
| ***Pantoea*** | ***Pantoea agglomerans***  **OsEp-AN-15A8** | ***Pantoea agglomerans***  **OsEp-AN-30A14** | ***Pantoea agglomerans***  **OsEp-AN-30A21** |
|  |  |  |  |
|  | ***Pantoea ananatis***  **OsEp-AN-15A10** | ***Pantoea ananatis***  **OsEp-AN-30A19** | ***Pantoea ananatis***  **OsEp-AN-30A5** |
|  |  |  |  |
|  | ***Pantoea ananatis***  **OsEp-AN-30A8** | ***Pantoea ananatis***  **OsEp-Plm-15P9** | ***Pantoea ananatis***  **OsEp-Plm-30P21** |
|  |  |  |  |
|  | ***Pantoea ananatis***  **OsEp-Plm-30P3** | ***Pantoea dispersa***  **OsEp-AN-30A18** | ***Pantoea eucrina***  **OsEp-AN-15A4** |
|  |  |  |  |
|  | ***Pantoea eucrina***  **OsEp-Plm-15P14** | ***Pantoea eucrina***  **OsEp-Plm-30P10** | ***Pantoea sp.***  **OsEp-AN-15A15** |
|  |  |  |  |
|  | ***Pantoea sp.***  **OsEp-AN-15A9** |  |  |
|  |  |  |  |
| ***Pseudomonas*** | ***Pseudomonas oryzihabitans***  **OsEp-Plm-15P6** | ***Pseudomonas parafulva***  **OsEp-Plm-15P12** | ***Pseudomonas psychrotolerans***  **OsEp-AN-15A6** |
|  |  |  |  |
|  | ***Pseudomonas psychrotolerans***  **OsEp-AN-30A13** | ***Pseudomonas putida***  **OsEp-Plm-15P11** |  |
|  |  |  |  |
| ***Sphingomonas*** | ***Sphingomonas paucimobilis***  **OsEp-AN-15A3** | ***Sphingomonas paucimobilis***  **OsEp-AN-30A9** | ***Sphingomonas pseudosanguinis***  **OsEp-AN-30A10** |
|  |  |  |  |
|  | ***Sphingomonas pseudosanguinis***  **OsEp-Plm-15P2** | ***Sphingomonas sp.***  **OsEp-AN-30A15** | ***Sphingomonas sp.***  **OsEp-Plm-15P5** |
|  |  |  |  |
|  | ***Sphingomonas yabuuchiae***  **OsEp-AN-30A3** |  |  |
|  |  |  |  |
| ***Mock*** | ***Mock*** | ***Mock*** |  |
|  |  |  |  |

**Supplementary Fig. 11. Volatile mediated *in vitro* antifungal activity of rice phyllosphere bacterial isolates on *Magnaporthe oryzae***

|  |  |  |
| --- | --- | --- |
| ***Pantoea ananatis***  **OsEp-Plm-15P9** | ***Pseudomonas putida***  **OsEp-Plm-15P11** | ***Pseudomonas parafulva***  **OsEp-Plm-15P12** |
|  |  |  |
| ***Pantoea eucrina***  **OsEp-Plm-15P14** | ***Acinetobacter soli***  **OsEp-Plm-30P4** | ***Aureimonas* sp.**  **OsEp-Plm-30P7** |
|  |  |  |
| ***Pantoea eucrina***  **OsEp-Plm-30P10** | ***Acinetobacter baumannii***  **OsEp-Plm-30P11** | ***Acinetobacter baumannii***  **OsEp-Plm-30P17** |
|  |  |  |
| ***Pantoea ananatis***  **OsEp-Plm-30P21** | ***Pantoea eucrina***  **OsEp-AN-15A4** | ***Pantoea ananatis***  **OsEp-AN-30A5** |
|  |  |  |
| ***Pantoea ananatis***  **OsEp-AN-30A8** | ***Pantoea agglomerans***  **OsEp-AN-30A14** | ***Pantoea dispersa***  **OsEp-AN-30A18** |

**Supplementary Fig. 12. Analysis of nature of BVC mediated growth inhibition of *Magnaporthe oryzae;*** Note: In each image; Top left plate: Mock from the beginning; Right plate: Mock from the day when volatile exposure in removed; Bottom plate: Plate incubated after removal of bacterial volatile exposure

| *Pantoea ananatis* OsEp-Plm-30P3 | *Pantoea ananatis* OsEp-Plm-30P21 | *Aureimonas* sp. OsEp-Plm-30P7 | *Pantoea ananatis* OsEp-AN-30A8 | *Pantoea eucrina* OsEp-Plm-30P10 | *Pseudomonas putida* OsEp-Plm-15P11 | *Pantoea ananatis* OsEp-Plm-15P9 | *Acinetobacter baumannii* OsEp-Plm-30P11 | *Pantoea eucrina* OsEp-AN-15A4 | *Pantoea ananatis* OsEp-AN-15A10 | *Pantoea* sp. OsEp-AN-15A15 |  |
| --- | --- | --- | --- | --- | --- | --- | --- | --- | --- | --- | --- |
| **R** | **R** | **R** | **R** | **R** | **R** | **MR** | **MR** | **MR** | **MR** | **MR** | |

| *Pantoea eucrina* OsEp-Plm-15P14 | *Acinetobacter baumannii* OsEp-Plm-30P17 | *Pantoea agglomerans* OsEp-AN-30A14 | *Pantoea ananatis* OsEp-AN-30A5 | *Pantoea dispersa* OsEp-AN-30A18 | *Pseudomonas parafulva*OsEp-Plm-15P12 | *Exiguobacterium indicum*OsEp-AN-30A4 | *Acinetobacter soli*OsEp-Plm-30P4 | *Erwinia tasmaniensis* OsEp-AN-15A5 | **Control (Mock)** | **Tricyclazole control** |  |
| --- | --- | --- | --- | --- | --- | --- | --- | --- | --- | --- | --- |
| **MR** | **MR** | **MR** | **MR** | **MS** | **MS** | **MS** | **MS** | **MS** | **S** | **R** | |

**Supplementary Fig. 13. Effect of phyllobacterization on rice blast disease incited by *Magnaporthe oryzae***

|  |  |
| --- | --- |
|  |  |
|  |  |
|  |  |

**Supplementary Fig. 14. qPCR based transcriptional analysis of defense genes expression in rice seedlings upon phyllobacterization;** The fold change values calculated for the defense genes expression were imported into the GraphPad Prism program (https://www.graphpad.com/scientific-software/prism) and two way ANOVA was conducted using Bonferroni Post-hoc test for determining the statistical significance at *P<=0.05, **P= 0.001 and ***P= 0.0001.
